# Synthesis and Antibacterial Activity of Novel Phosphonated CF_3_-β-lactams

**DOI:** 10.1021/acsomega.5c01562

**Published:** 2025-04-28

**Authors:** Monika Skibinska, Alicja Warowicka, Benoît Crousse, Tomasz Cytlak

**Affiliations:** † Faculty of Chemistry, Adam Mickiewicz University, Uniwersytetu Poznańskiego 8, 61-614 Poznań, Poland; ‡ Faculty of Biology, Adam Mickiewicz University, Uniwersytetu Poznańskiego 6, 61-614 Poznań, Poland; § BioCIS UMR 8076 CNRS, Building Henri Moissan, Université Paris-Saclay, avenue des sciences, 91400 Orsay, France; ∥ Centre for Advanced Technologies, Adam Mickiewicz University, Uniwersytetu Poznańskiego 10, 61-614 Poznań, Poland

## Abstract

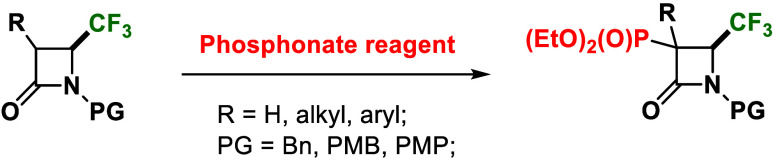

A new series of C-3 phosphonated 4-CF_3_-*β*-lactams was stereoselectively synthesized from corresponding 4-CF_3_-*β*-lactams, applying two different protocols for phosphonate group incorporation. The first method involved the direct incorporation of a phosphonate (V) moiety at the C-3 position although it was limited by steric hindrance. The second approach enabled the incorporation of a less bulky phosphonite (III), which was subsequently oxidized to the corresponding phosphonate (V). The synthesized *β*-lactam ring features both fluorinated and phosphonate substituents, which are known for their biological significance, such as enhancing membrane permeability, improving binding interactions, and inhibiting enzymes. Considering these properties, along with the inherent antibacterial potential of *β*-lactams, we evaluated the antibacterial activity of selected C-3 phosphonated 4-CF_3_-*β*-lactams against four bacterial strains (*Staphylococcus aureus* (*S. aureus*), methicillin-resistant *Staphylococcus aureus* (MRSA), *Neisseria gonorrheae*, *Escherichia coli* (*E. coli*)). Applying the disk diffusion method, MIC measurements, and *β*-lactamase inhibition assays, compounds **11** and **16** emerged as the most promising candidates in this preliminary antibacterial evaluation.

## Introduction

The elemental phosphorus is widespread in the world of living organisms.^1^ Due to the wide range of natural and synthetic compounds containing phosphorus, they are of interest to the agrochemical, medicinal, and bioorganic chemistry industries.^2^ The phosphonate group (R–C-P) could be recognized as isosteric or bioisosteric analogues of the phosphate moiety (R–O-P), commonly found in biologically significant substrates, due to their significant properties, including resistance to phosphatase cleavage.^3,4^ Among them, aminophosphonates have gained special attention because of their valuable utility as enzyme inhibitors, antibiotics, herbicides, and antifungal agents.^5,6^ Moreover, *α*- or *β*-aminophosphonates could be considered structural analogues of the amino acids, as described in the literature.^7−12^

**Figure 1:**
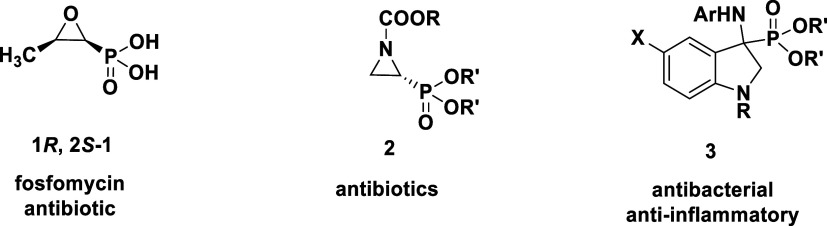
Representative bioactive phosphorus-substituted heterocycles.

Incorporating the phosphonate group into small heterocycles can create new opportunities in medicinal and synthetic chemistry, facilitating the development of bioactive molecules and versatile building blocks.^13,14^ In the literature, there are already documented examples of this type of cooperation (Figure 1).^14−22^

**Figure 2:**
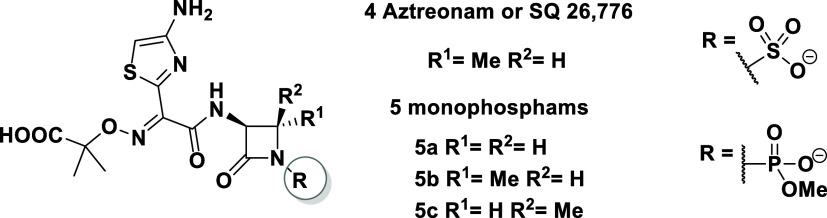
Antibacterial Aztreonam (monobactam) and analogous monophosphams.

Hence, combining the *β*-lactam ring with a phosphonate moiety could be intriguing for the development of potential bioactive compounds or intermediates used in synthesizing complex phosphonated aza-compounds (Figure 2),^23−25^ which have received less attention than their five- or six-membered analogues.^24,26^ This highlights the potential of small heterocyclic rings both as bioactive agents and as synthetic intermediates in medicinal chemistry.^27^

To the best of our knowledge, the limited number of literature reports on phosphonated *β*-lactams has resulted in a lack of biological studies on these compounds. However, based on the known effects of the phosphonate group on amide analogues, it can be assumed that phosphonate substitution in *β*-lactams may enhance stability, improve enzyme binding, broaden antibacterial activity, and reduce resistance.^28−31^ Furthermore, phosphonated *β*-lactams serve as excellent building blocks for the synthesis of more complex biologically active compounds, a strategy referred to as the “*β*-lactam synthon method.”^32,33^ These features make phosphonated *β*-lactams promising for the development of next-generation antibiotics.

Moreover, incorporating a fluorinated substituent into the *β*-lactam structure can impact its stability and potential bioactivity.^34,35^ Additionally, the bulky CF_3_ group is often employed to mimic the side chain of various amino acids involved in ligand interactions of enzyme inhibitors,^36^ as well as modifying the agonist–antagonist nature of ligands.^37^ Furthermore, replacing the carbonyl group (C=O) of peptides with a CF_3_ group can produce a stable and nonbasic amine that retains excellent hydrogen bonding, a technique widely used in designing various enzyme inhibitors.^38−40^ The CF_3_ group is a common substituent in bioactive compounds, often used to modulate drug candidate activity, block random metabolism, and improve pharmacokinetic profiles.^41^ Despite these advantages and the well-established importance of the *β*-lactam ring in medicinal chemistry,^42^ there are currently no known biologically active compounds with the CF_3_ group directly attached to the *β*-lactam ring. Most CF_3_-containing bioactive compounds possess this group on aromatic or heteroaromatic rings.^43,44^ This gap opens new perspectives for the design of novel fluorinated *β*-lactams and the investigation of their antibacterial activity.^45,46^

For all these reasons, fluorinated phosphonates are frequently utilized as building blocks in the synthesis of biologically active compounds, such as fluorinated phosphonate peptide analogues.^10−12,47^ The synergistic effect of combining phosphonate motifs with fluorinated moieties is well-documented in the literature, often resulting in enhanced enzyme inhibition of the parent compounds due to improved membrane permeability and increased binding affinity.^48^

The number of documented synthetic methods concerning the phosphono-*β*-lactams is generally limited, primarily focusing on forming a four-membered ring or modifying the side chains of compounds already possessing a *β*-lactam backbone.^15−17,24,26,49−66^

Therefore, the aforementioned facts provide a promising field to study organophosphorus and organofluorine chemistry further, particularly focusing on the exploration of phosphonated and fluorinated *β*-lactams. Referring to protocols reported in the literature, no examples describe the synthesis of *β*-lactams bearing phosphonate and CF_3_ moieties directly bonded to the ring. It prompted us to investigate the preparation of phosphonated derivatives of 4-CF_3_-*β*-lactam and to evaluate the biological studies of the obtained compounds toward antibacterial activities.

## Results and Discussion

### Chemistry

We previously reported the stereoselective synthesis of C-3 mono- and disubstituted 4-CF_3_-*β*-lactams.^67^ Following this work, we planned to incorporate a phosphonate group at the C-3 position into 3-mono- and 3-unsubstituted 4-CF_3_-*β*-lactams.

Therefore, we performed the reaction of the racemic mixture of *N*-Bn 4-CF_3_-β-lactam **6** with diethyl chlorophosphate according to the analogous protocol referred to in our previous paper, which concerned the generation of enolate ion **7** and its subsequent reaction with various electrophiles (1.5 equiv of LiHMDS at −25 °C).

Initially, the use of 2 equiv of diethyl chlorophosphate under standard reaction conditions, as well as increasing the amount of LiHMDS to 2 equiv, did not yield any product, and only unreacted starting material was observed in the reaction mixtures (Table 1, entry 1). Thus, in the next attempts, we tested an excess of LiHMDS (3 equiv) and other non-nucleophilic organolithium bases (3 equiv) such as LTMP, LDA, and *n*-BuLi at −78 °C for 2 h. Reactions were monitored by ^19^F and ^31^P NMR analyses. Consequently, despite the fact that in all reactions the formation of any product was not observed after 2 h, the reaction mixtures were stirred overnight at room temperature (Table 1, entries 2–5). Finally, only the reaction with an excess of LiHMDS (3 equiv) gave the desired *N*-Bn phosphonated 4-CF_3_-*β*-lactam **8** in 9% yield (Table 1, entry 5). Due to this one promising result with LiHMDS, further tests were carried out to select the best conditions with the intention of increasing substrate conversion. According to the foregoing outcome, the 4 equiv of LiHMDS was used in the following approach. However, in this case, the product **8** was still obtained in unsatisfactory yield (11%) (Table 1, entry 6). For that reason, we decided to increase the amount of diethyl chlorophosphate from 2 to 3 and 6 equiv (Table 1, entries 7–8). Conveniently, this modification has allowed the desired product **8** to be obtained with significantly increased yields (52 and 68%). The two last attempts were based on temperature variations. It is noteworthy that the product was also formed at −25 °C, using 3 equiv of LiHMDS and 6 equiv of phosphonate reagent with a good 70% conversion, while at −10 °C, no reaction was observed (Table 1, entries 9–10). The overall yield of isolated *N*-Bn phosphonated 4-CF_3_-*β*-lactams **8** was 60%.

**Table 1:**
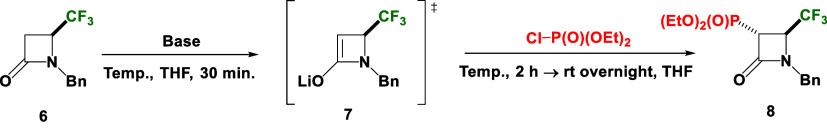
Different Conditions of Synthesis *N*-Bn Phosphonated 3-CF_3_-*β*-lactam **8**

entry	base	Cl–P(O)(OEt)_2_	temp °C	**8**, yield % (^19^F, ^31^P NMR)
1	LiHMDS, 1.5–2 eq.	2 eq.	–25	n.r.^a^
2	LTMP, 3 eq.	2 eq.	–78	n.r.
3	LDA, 3 eq.	2 eq.	–78	n.r.
4	*n*-BuLi, 3 eq.	2 eq.	–78	n.r.
5	LiHMDS, 3 eq.	2 eq.	–78	9%
6	LiHMDS, 4 eq.	2 eq.	–78	11%
7	LiHMDS, 3 eq.	3 eq.	–78	52%
8	LiHMDS, 3 eq.	6 eq.	–78	68%
9	LiHMDS, 3 eq.	6 eq.	–25	70%
10	LiHMDS, 3 eq.	6 eq.	–10	n.r.

a No reaction, starting material was recovered with >85% rate.

**Scheme 1:**
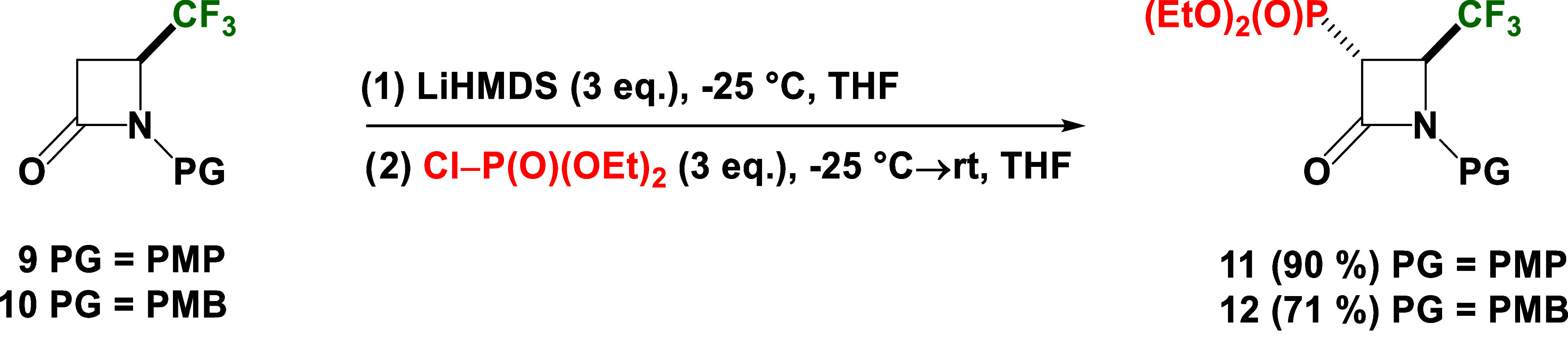
Synthesis of *N*-PMP and *N*-PMB Phosphonated 4-CF_3_-*β*-lactams **11** and **12**

Then, the substitution reactions at the C-3 position were verified with racemic mixtures of *N*-PMP and *N*-PMB 4-CF_3_-*β*-lactams **9** and **10**, respectively. Surprisingly, in these cases, the substitution of the C-3 position proceeded very smoothly with 3 equiv of diethyl chlorophosphate and 3 equiv of LiHMDS, affording the corresponding phosphonated 4-CF_3_-*β*-lactams **11** and **12** in excellent yields, 90 and 71%, respectively (Scheme 1). The reactions were also performed with 2 equiv of LiHMDS but with evidently lower yields. On the other hand, increasing the amount of LiHMDS (4 equiv) and/or diethyl chlorophosphate (6 equiv) did not affect the reaction results.

The relative *trans* configuration of the isolated products **8**, **11**, and **12** was determined based on the ^1^H NMR (the coupling constants between H-3 and H-4 are about 2.5 Hz, which suggests their *trans* arrangement). Similarly, the interpretation of 2D ^1^H–^1^H NOESY NMR analysis of compound **11** proved that there is no observable interaction between H-3 and H-4 (see Supporting Information). This also indicated the relative *trans* configuration of *N*-PMP phosphonated 4-CF_3_-*β*-lactam **11**. Hence, the results of the 2D ^1^H–^1^H NOESY NMR experiment for *N*-Bn and *N*-PMB phosphonated 4-*β*-lactams **9** and **12** were analogous.

The example of the reaction of C-3 substituted nonfluorinated *γ*-lactam (3-methylpyrrolidin-2-one derivative) with diethyl chlorophosphate (V) was undertaken and described in the literature as unfeasible because of the fact that during the reaction, the formed vinyl phosphate anion could not undergo further rearrangement to phosphonate, prevented by the 3-Me group.^68−71^ Despite this literature report, we decided to carry out some experiments with C-3-substituted 4-CF_3_-*β*-lactams.

**Scheme 2:**
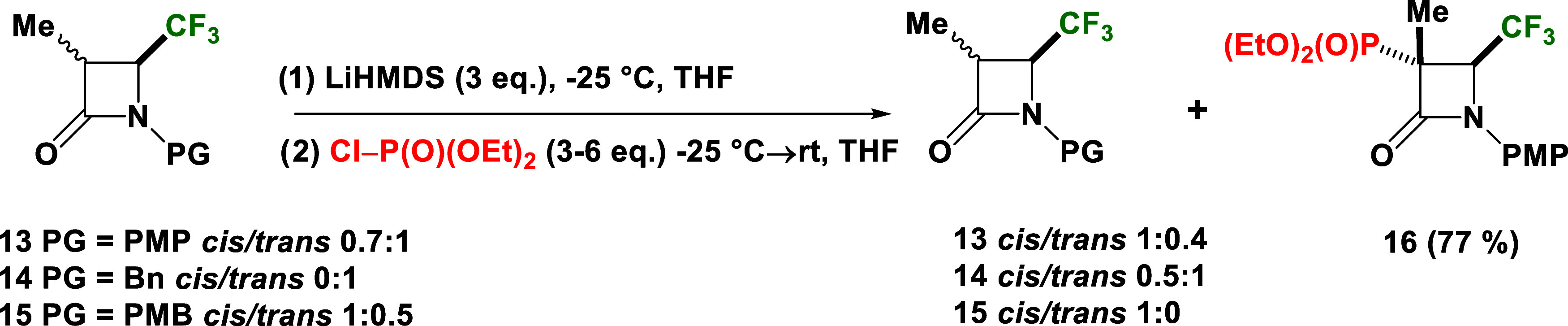
Synthesis of *N*-PMP Phosphonated 3-Me-4-CF_3_-*β*-lactam **16**

In the case of 3-Me-4-CF_3_-*β*-lactam **13**–**15** as a starting material (*cis/trans* mixture), only phosphonated *N*-PMP 3-Me-4-CF_3_-*β*-lactam **16** was obtained with a 77% conversion rate based on ^19^F NMR spectra (Scheme 2), with the presence of the substrate **13** only in *trans* conformation. In the case of *N*-Bn **14** and *N*-PMB **15** substrates, we did not observe the formation of phosphonated products, but only isomerization of the starting mixture was observed, leading to an increase in the amount of the *cis* isomer while the *trans* isomer amount decreases. Increasing the number of diethyl chlorophosphate equivalents from 3 to 6 did not affect the result or quenching of the reaction during the same day.

**Scheme 3:**
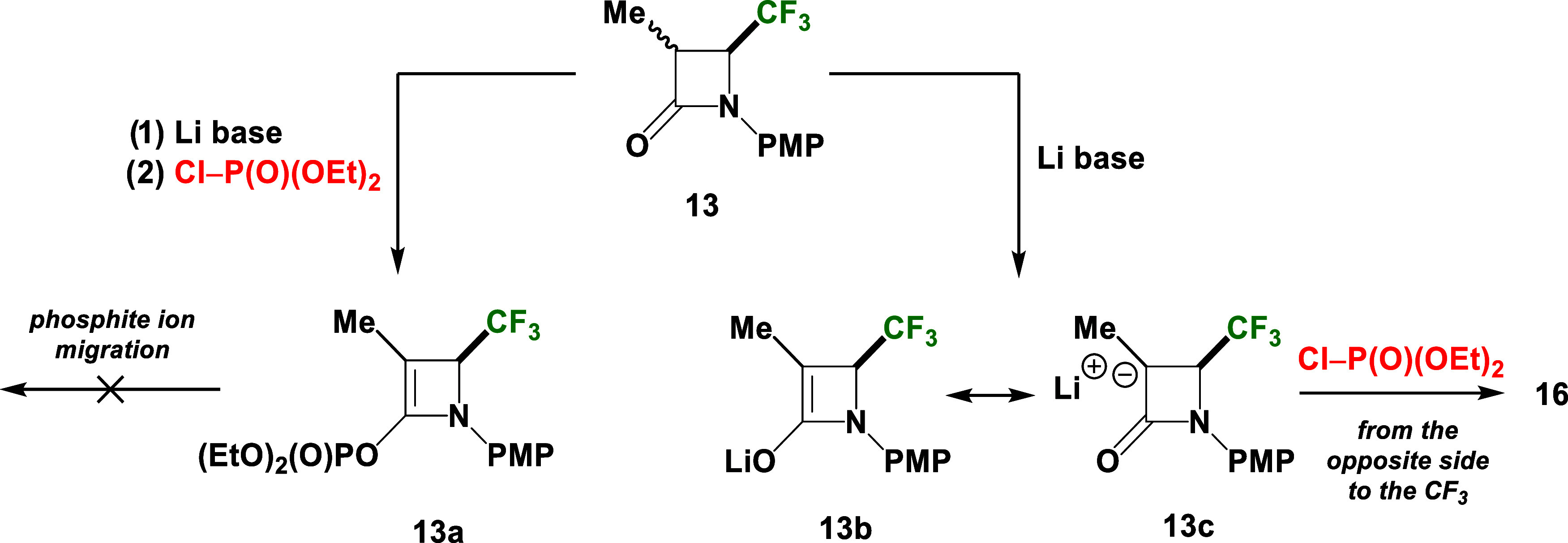
Suggested Reaction Pathway Involes Enolate Ion Intermediates **13b** and **13c**, Formed during the Reaction of **13** with ClP(O)(OEt)_2_ in the Presence of LiHMDS

In contrast to previous literature findings,^71^ it was suggested that the reaction of **13** proceeds via the formation of vinyl phosphate **13a**. This would imply that the presence of a 3-Me substituent should prevent the rearrangement of phosphate **13a** to phosphonate **16** through phosphite ion migration (Scheme 3), thereby reverting the reaction back to substrate **13**. However, the formation of **16** in good yield suggests that, in this case, the reaction proceeds through enolate anion intermediates **13b** and **13c**, stabilized by the strong electron-withdrawing CF_3_ group. The involvement of the enolate ion explains why the addition of the phosphonate moiety to 3-Me-4-CF_3_-*β*-lactam **13** proceeded via a kinetically controlled pathway, occurred from the opposite side to the CF_3_ group, regardless of whether the substrate had a relative *trans* or *cis* configuration, analogous to the results reported in our previous work.

The presence of unreacted substrates **14** and **15** with a predominance of *cis* isomers was due to an isomerization/racemization process. Consequently, the reaction mixture was characterized by the same stereoselectivity.

The relative configuration of the new phosphonated 3-Me-4-CF_3_-*β*-lactam **16**, in which the CF_3_ group is in a *cis* relation to the Me moiety, was established based on 2D ^1^H–^19^F HOESY NMR spectra. In this NMR experiment, the interaction of the CF_3_ group with the Me substituent was observed, indicating their proximity in space (see Supporting Information).

**Scheme 4:**
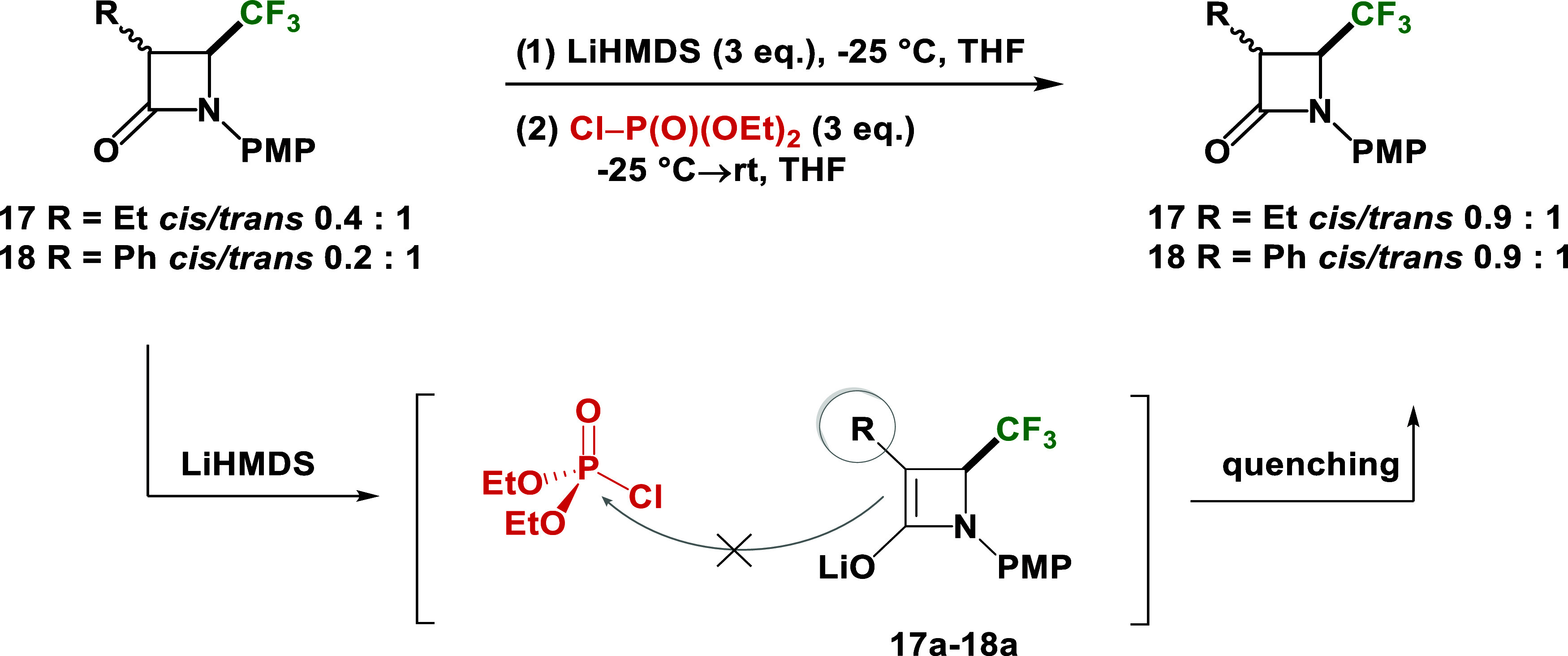
Synthesis Attempts of *N*-PMP Phosphonated 3-Et- and 3-Ph-4-CF_3_-*β*-lactams

This unexpected result, contrary to the literature data,^71^ prompted us to perform this reaction with other C-3 monosubstituted *β*-lactams. Unfortunately, in the remaining cases, phosphonated C-3 monosubstituted-4-CF_3_-*β*-lactams were not obtained. However, in the cases of *cis/trans* mixture of diastereoisomers of 3-Et-4-CF_3_-*β*-lactam **17** and 3-Ph-4-CF_3_-*β*-lactam **18** used in the reactions, the recovered substrates demonstrated an increasing ratio of the *cis* isomer to the *trans* isomer (>85% of recovery by column chromatography in each case). This fact suggests that the enolate ion was generated *in situ* during the reaction, but it did not react with diethyl chlorophosphate. Consequently, during the completion of the reaction, the protonation proceeded kinetically, leading to the formation of the *cis* isomer, which has less steric hindrance (Scheme 4). When we performed the test reaction of 3-Me-4-CF_3_*-β*-lactam **13** (*cis*/*trans* 0.4:1) under the same conditions, but without the presence of any electrophiles, only quenching the reaction with NH_4_Cl, we observed analogous isomerization toward major *cis* isomer (*cis*/*trans* 1:0.6). When the reaction was quenched with MeOD-d4, we observed the same isomerization ratio (*cis*/*trans* 1:0.6) of C-4 deuterated 3-Me-4-CF_3_*-β*-lactam (see Supporting Information). In the case of the other C-3 monosubstituted-4-CF_3_-*β*-lactams (*trans* isomers of 3-Allyl-, 3-Bn-, and 3-CO_2_Et-4-CF_3_-*β*-lactams), reactions occurred with partial decomposition without formation of phosphonated lactams (see Supporting Information). Thus, the addition of phosphonate moiety did not occur, probably due to the steric hindrance of the Et, Ph, Allyl, Bn, and CO_2_Et groups at the C-3 position.

**Scheme 5:**
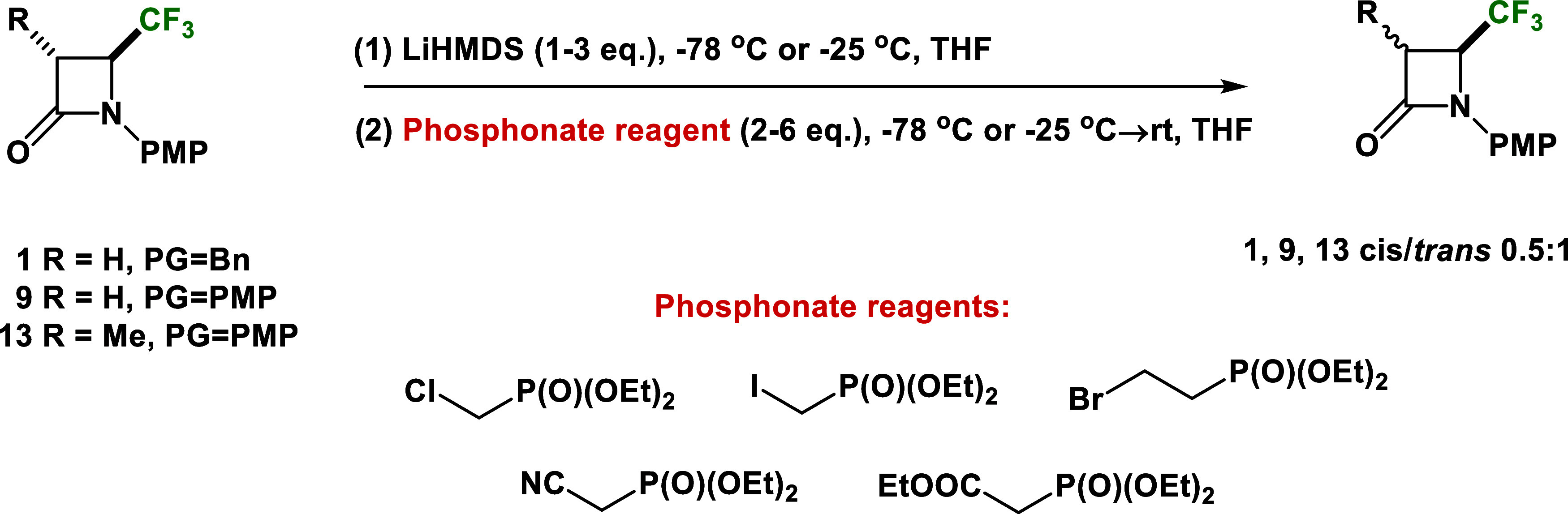
Attempts to the Synthesis of Various Phosphonated 4-CF_3_-*β*-lactams

Subsequently, we tried to introduce diethyl methylphosphonate and diethyl ethylphosphonate at the C-3 position of *N*-Bn and *N*-PMP 4-CF_3_-*β*-lactam rings (Scheme 5). Modifications of conditions, such as the temperature and the number of equivalents of the base or the phosphonated reagent, did not yield the desired phosphonated 4-CF_3_-*β*-lactam derivatives. After these reactions, we observed only unreacted starting materials. Compound **13** was used as the pure *trans* isomer and recovered as the *cis*/*trans* mixture (0.5:1) (>85% of recovery by column chromatography in each case).

**Scheme 6:**
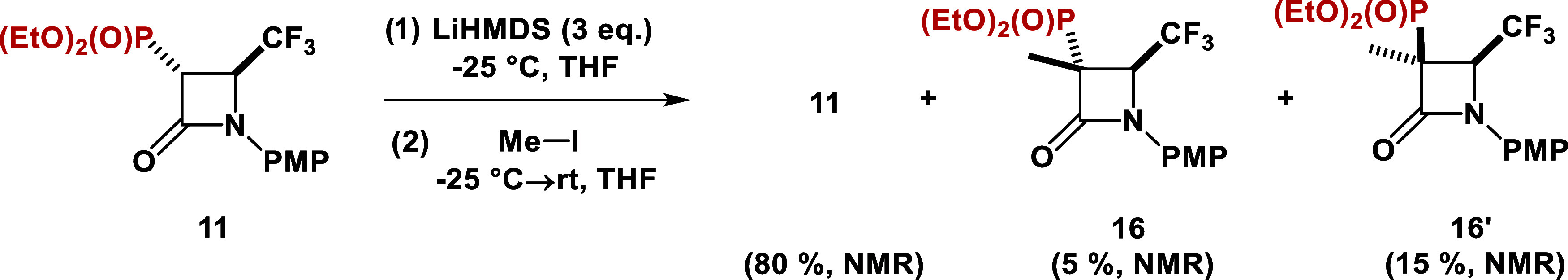
Attempt to C-3 Methylation of 3-Phosphonated 4-CF_3_-*β*-lactam

Also, conversely, the introduction of another substituent, such as alkyl at the C-3 position of 3-phosphonated 4-CF_3_-*β*-lactam **11**, was undertaken (Scheme 6). According to the previously developed C-3 substitution procedure, the intended kinetically favored 3-Me-3-phosphono-4-CF_3_-*β*-lactam **16′** (with the phosphonate moiety *cis* to CF_3_) was obtained in 15% yield (^19^F, ^31^P NMR). Moreover, we observed the formation of **16** (with the phosphonate moiety *trans* to CF_3_) in 5% yield (^19^F, ^31^P NMR) and unreacted substrate **11** at 80% content in the mixture (see Supporting Information). The addition of an electrophile is limited probably due to the bulkiness of the phosphonate and CF_3_ groups.

To exemplify the synthesis of phosphonated 4-CF_3_*-β*-lactams, we decided to try the methodology described by Wiemer et al.^71^ They, therefore, developed the reaction of different lactams with diethyl chlorophosphite (Cl–P(OEt)_2_) in the presence of LDA (as well as LiHMDS) and subsequent oxidation of P(III) to P(V) using hydrogen peroxide (30% in water).

**Scheme 7:**
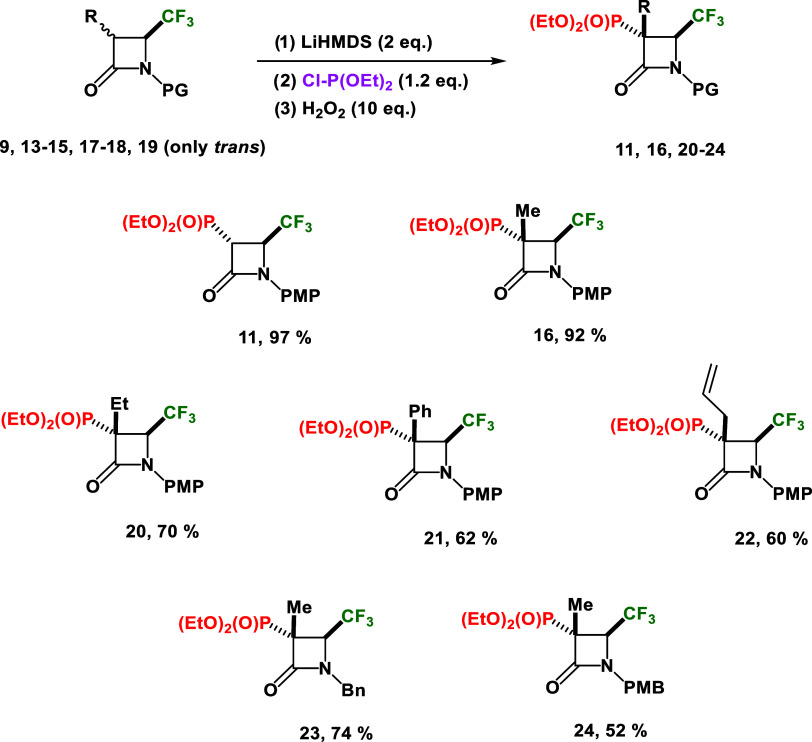
Scope of Different Phosphonated 4-CF_3_-*β*-lactams Synthesized Using Diethyl Chlorophosphite

Thankfully, when conditions were realized on *N*-PMP 4-CF_3_-*β*-lactam **9**, the corresponding *N*-PMP 3-phosphonated *β*-lactams **11** was obtained in excellent yield (97%). These conditions are also very favorable for the 3-Me-4-CF_3_-*β*-lactam **13** and led to *N*-PMP 3-phosphonated *β*-lactams **16** in 92% yield. If we compare these results to the previous conditions (Schemes 1 and 2), the two-step method gives an equivalent result for 4-CF_3_-*β*-lactam **9**. However, the result is considerably improved in the case of 3-Me-4-CF_3_-*β*-lactam **13**. Faced with these very convincing results, we extended the family of C-3-substituted 3-phosphonated *β*-lactams, resulting in products **20–24** (Scheme 7).

The relative *cis* configuration of the CF_3_ group in relation to the Me moiety for **23** and **24** was established based on 2D ^1^H–^19^F HOESY NMR spectra (see Supporting Information). These observations confirmed that the stereochemistry of both previous conditions (diethyl chlorophosphate (V) vs diethyl chlorophosphite (III)) is in agreement. At the same time, we also attempted to synthesize *N*-PMP bisphosphonate at the C-3 position.^71^ Both approaches, the one-pot reaction with 2 equiv of diethyl chlorophosphite and the two-step synthesis with isolated monophosphonate **11**, did not yield the expected bisphosphonate.

### Biology

**Figure 3:**
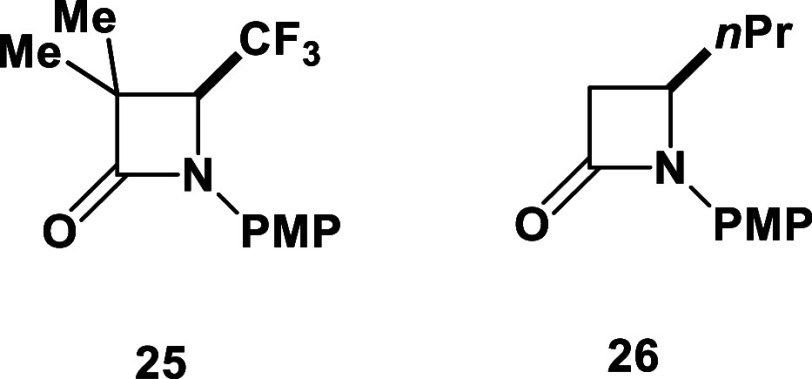
Reference compounds.

The literature describes various main therapeutic strategies for overcoming bacterial resistance to *β*-lactams. The first strategy involves designing antibiotics that imitate *β*-lactams and do not undergo *β*-lactamase-catalyzed hydrolysis. The second strategy is to use a *β*-lactamase inhibitor in combination with a standard *β*-lactam antibiotic.^24,26^ Another strategy is based on the modification of the substituent(s) introduced in the structure of the *β*-lactam ring.^72^ Due to the unique presence of fluorinated and phosphonate substituents in the *β*-lactam ring, this prompted us to investigate their impact on the potential biological activity of the obtained compounds. Therefore, we decided to perform the preliminary antibacterial evaluation of newly synthesized phosphonated 4-CF_3_-*β*-lactams **8**, **11**, **12**, **16**, **and 20–24** against Gram-positive bacteria: *Staphylococcus aureus* (*S. aureus*, ATCC 25923); methicillin-resistant *Staphylococcus aureus* (MRSA, ATCC 43300) and Gram-negative bacterial strain: *Escherichia coli* (*E. coli*, ATCC 25922); *Neisseria gonorrheae* (ATCC 43069). As references for these phosphonated lactams, we selected to evaluate corresponding monosubstituted nonphosphonated *β*-lactam (*N*-PMP 3,3-diMe-4-CF_3_-*β*-lactam **25** and *N*-PMP 3-Ph-4-CF_3_-*β*-lactam **18**) and an example of C-3 unsubstituted nonfluorinated *β*-lactam (4-*n*-Pr-*β*-lactam **26**), previously obtained in our laboratory (Figure 3).^46,67^ The antibacterial activity was evaluated by the disk diffusion assay, and the compounds were also tested by the minimum inhibitory concentration (MIC). The reference antibiotic (rifampicin, cat. no. R3501, Sigma-Aldrich) was used as a positive control for the diffusion assay as well as for the MIC method. Finally, selected compounds were tested toward the *β*-lactamase inhibition activity.

The agar disk diffusion assay results showed that the tested compounds present inhibitory activity against bacteria. Moreover, the studied compounds have different effects against various bacterial strains (Table 2 and Figures 1–4 in SI). Smaller zones of growth inhibition were detected for Gram-positive strains: *S. aureus* and *MRSA*, approximately at the same levels (1–2.5 mm). Furthermore, compounds **8** and **12** showed the same activity toward *S. aureus* and *MRSA* (the zone of growth inhibition was 1.5 mm), whereas other studied compounds (**11**, **16**, **18**, and **23**–**24**) presented a stronger inhibition effect against *S. aureus* than *MRSA*. Notably, we observed a higher activity against Gram-negative strains. Against *E. coli*, the least active was compound **11**, while the most active compound was **21**, followed by the nonphosphonated reference **25** (Table 2). Interestingly, against *N. gonorrheae*, we observed complete inhibition zones (fully transparent) around 2 mm, but we also observed visible, measurable inhibition zones (increasing in transparency) with diameters of 4–8 mm (probably heterogeneity within the bacterial population), where compounds **11** and **21** exhibited the largest zone of inhibition. Compared to nonphosphonated *β*-lactams (**18** and **25**) reference, compound **25** exhibited the best results against *MRSA* and *E. coli* (and against *S. aureus*, comparable to phosphonated *β*-lactams we observed), but we noticed the much lower activity of **25** against *N. gonorrheae*. On the other hand, the other nonphosphonated compound (**18**) exhibited much lower activity against all strains. The effect of the phosphonate substituent is particularly noticeable when we compared the results of **18** to its phosphonated analogue (**21**). Importantly, the nonfluorinated *β*-lactam **26** did not show any activity against *S. aureus*, *MRSA*, and *E. coli* strains, confirming the effect of fluorine on the appearance of activity.

**Figure 4:**
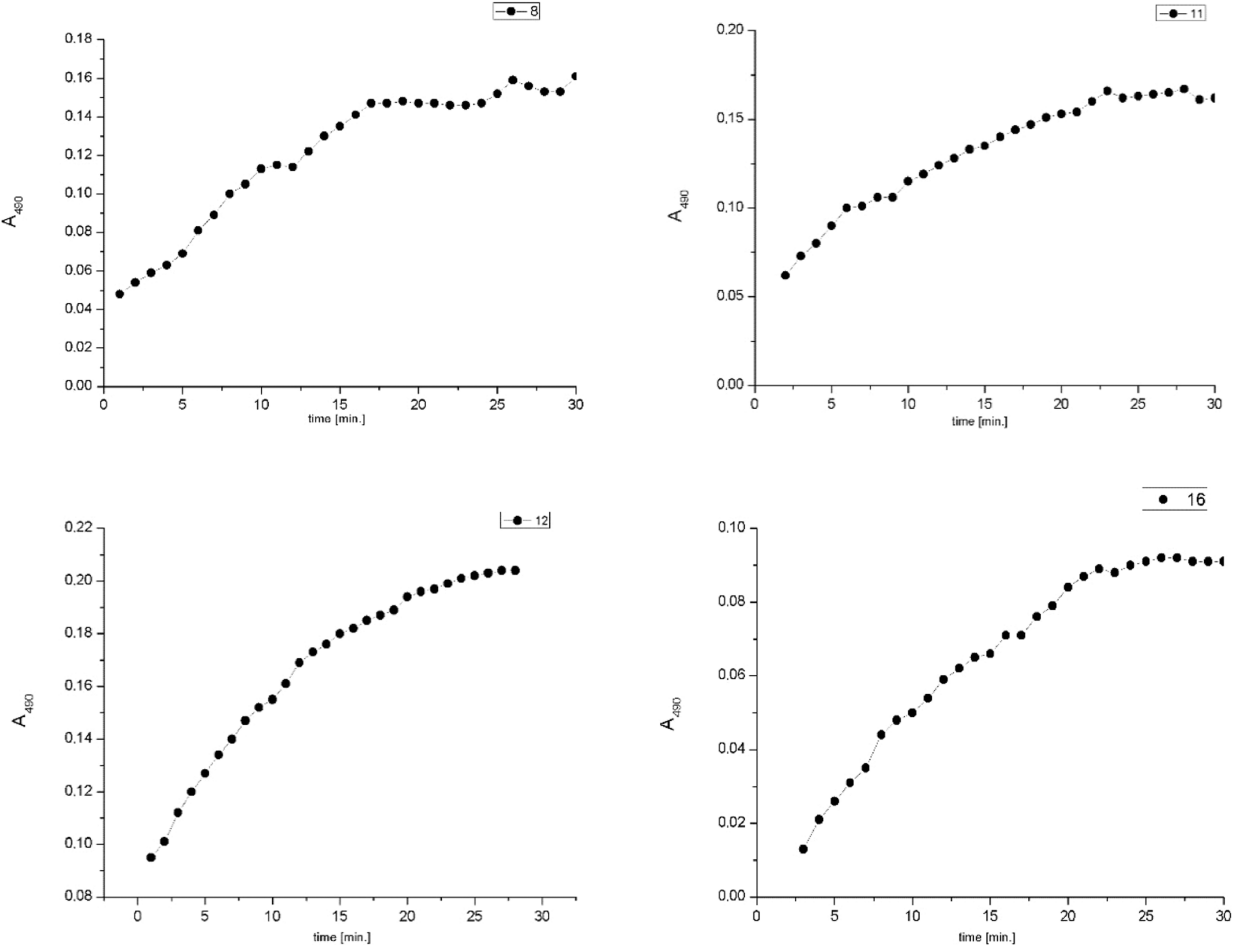
*β*-Lactamase activity in the presence of compounds **8**, **11**, **12**, and **16**. Absorbance at 490 nm (*A*_490_) was measured every minute, for 30 min using a microplate spectrophotometer.

**Table 2:** Inhibition Zone Diameters (mm)

	antibacterial activity (zone diameters in mm)			
compound	S. aureus (ATCC 25923)	*MRSA* (ATCC 43300)	E. coli (ATCC 25922)	N. gonorrheae (ATCC 43069)^a^
**8**	1.5	1.5	2	2 (5)
**11**	2	1	1.5	2 (8)
**12**	1.5	1.5	2	2 (6)
**16**	2.5	1.5	3	2 (6)
**18**	1.5	1	3	1.5 (4)
**20**	1.5	1.5	3	2 (7)
**21**	2.5	1.5	4	2.5 (8)
**22**	1.5	1	3	2 (5)
**23**	2	1.5	2.5	n.t.^b^
**24**	1.5	1	2	n.t.^b^
**25**	2^c^	2^c^	5	1 (4)
**26**^a^	0	0	0	n.t.^b^

a Complete inhibition zones (measurable zones).

b Not tested.

c Images of plates of tested compound are included in SI of ref (58).

The MIC results (Table 3) revealed that among all tested compounds **11** and **16** exhibited considerable antibacterial activity against *S. aureus*. Against *MRSA* and *E. coli*, all tested compounds showed low levels of activity, with the only exception being the activity of the nonphosphonated reference compound (**25**) against *E. coli*. The highest activity against *N. gonorrheae* was achieved for compound **11**, with a good level of activity also shown for compounds **16** and **20**. Comparing the MIC results of 3-phosphonated *β*-lactams to the nonphosphonated reference (**18** and **25**), we observed better activity against *S. aureus* for *N*-PMP 3-phosphonated *β*-lactams, without other substituents bonded to C-3 (**11**) or with a small 3-Me substituent (**16**). Bulkier C-3 substituents decreased the activity level. Against *E. coli*, 3-phosphonated *β*-lactams showed lower activity than the nonphosphonated reference **25**. However, the activity of compound **18** was on the same level as the best phosphonated compounds (**11**, **16**). Conversely, against *N. gonorrheae*, the 3-phosphonated substituent (compared to **18** and **25**), together with the effect of *N*-PMP-protecting group (e.g., **11** vs **8** and **12**), increased the level of activity. In general, we observed higher activity of phosphonated *β*-lactams against *S. aureus*, especially against *N. gonorrheae*, and lower against *E. coli* compared to the nonphosphonated reference *β*-lactam (**25**).

**Table 3:** Antibacterial Activity of Selected/Synthesized Compounds (Minimal Inhibitory Concentration, MIC)

	antibacterial activity (MIC; μg/mL)			
compound	*S. aureus* (ATCC 25923)	*MRSA* (ATCC 43300)	E. coli (ATCC 25922)	N. gonorrheae (ATCC 43069)
**8**	>500	>500	500	125
**11**	16	250	125	16
**12**	500	500	500	125
**16**	31	500	125	63
**18**	125	500	125	250
**20**	125	250	>500	63
**21**^a^	n.t.^b^	n.t.^b^	n.t.^b^	n.t.^b^
**22**	125	500	>500	250
**23**	>500	>500	500	125
**24**	>500	>500	500	125
**25**	125	125	31	500
**26**	>500	>500	>500	500
**rifampicin**	1	2	8	n.t.^c^

a No measurement; when trying to dissolve compound **23** in DMSO, the solution became cloudy.

b Not tested.

c Not tested (according to EUCAST).^73^

**Figure 5:**
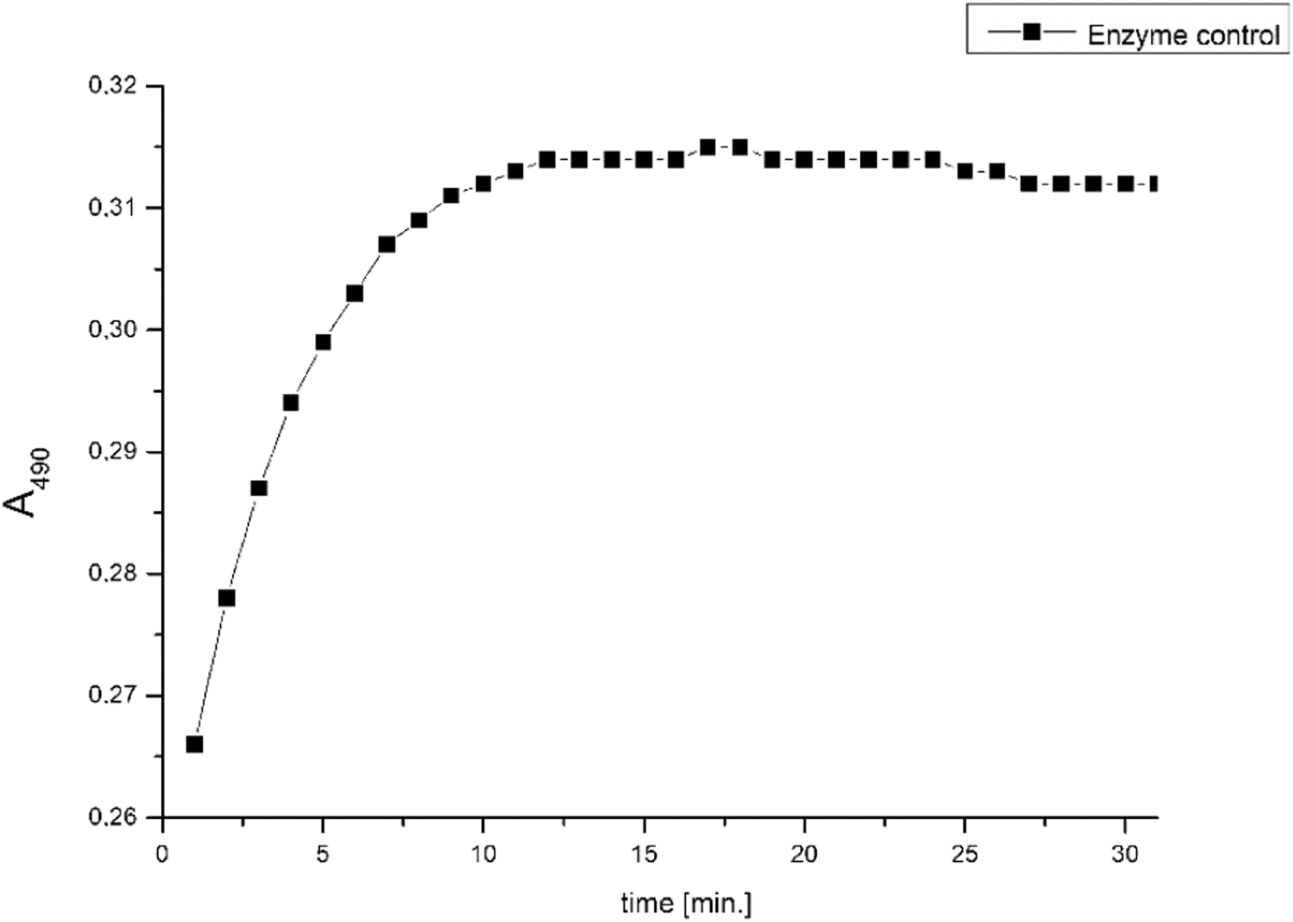
*β*-Lactamase activity in the absence of a potential inhibitor (enzyme control). Absorbance at 490 nm (*A*_490_) was measured every minute, for 30 min using a microplate spectrophotometer.

In order to evaluate if our selected compounds **8**, **11**, **12**, **and 16** can inhibit bacterial *β*-lactamases, a colorimetric β-lactamase Inhibitor screening kit assay was applied. In this convenient assay, the activity of *β*-lactamase was measured spectrophotometrically. The potential inhibitory activity of the compounds was determined by a colorimetric assay. The visual effect of the *β*-lactamase activity (hydrolysis of a chromogen nitrocefin, producing a colored product) was characteristic, pink in color, which indicates the hydrolysis of nitrocefin, a substrate for *β*-lactamase (see Supporting Information). Due to degradation (hydrolysis), nitrocefin changes color from yellow to light pink. Thus, the amount of produced color is directly proportional to the *β*-lactamase ( *β*Lac) enzyme activity. For inhibition efficiency evaluation, the % of relative inhibition was calculated. The absorbance (*A*_490_) was plotted versus time for each sample, and the slope of the plot (*A*_490_/min) was expressed (Figure 4). The % of relative inhibition was determined as follows
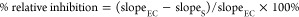
where slope_EC_ is the slope of the enzyme control (without inhibitor, Figure 5), and slope_S_ is the slope of the studied compound (potential inhibitor). Slope = (ABS2 – ABS1)/(T2 – T1) = ΔABS/min.

According to the literature,^74^ the most linear segment of the first part of the graph’s slope, corresponding to the initial reaction rate, was used for the analysis (Table 4).

**Table 4:** Relative Inhibition of Compounds **8**, **11**, **12**, and **16**

compound	slope_EC_	slope_S_	T2-T1 (min)^a^	relative inhibition (%)
**8**	0.0093	0.0068	14–5	27.1
**11**	0.0093	0.0059	14–2	36.8
**12**	0.0093	0.0067	11–1	29.3
**16**	0.0093	0.0048	16–3	48.2

a Part of the graph’s slope, as consecutive absorbance measurement points, which was used for analysis.

The results revealed that compounds **8**, **11**, **12**, and **16** exhibited a *β*-lactamase inhibition potential. Among them, compound **16** demonstrated the highest level of inhibition, with a 48.2% reduction in *β*-lactamase activity. Notably, the presence of compound **12** reduced the *β*-lactamase activity to 29.3%, while compound **8** reduced it to 27.1%. Interestingly, the type of protecting group on the nitrogen influenced the level of inhibition for phosphonated CF_3_-*β*-lactams, with the highest levels observed for *N*-PMP and lower levels for *N*-Bn and *N*-PMB. These observations confirm MIC results indicating the influence of *N*-protecting substituent on the level of activity. The results demonstrate that the selected compounds can suppress bacterial resistance to *β*-lactam antibiotics. Thus, our findings suggest that those compounds may hold potential for development as new *β*-lactam antibiotics and *β*-lactamase inhibitor agents.

## Conclusions

In conclusion, we synthesized the novel phosphonated CF_3_-*β*-lactams through reactions with two different electrophilic phosphorus reagents. The first method involved the direct introduction of the phosphonate (V) moiety at C-3 in the reaction of *β*-lactams with diethyl chlorophosphate (Cl–P(O)(OEt)_2_) under basic conditions. The second method involved the introduction of the phosphonite (III) moiety through the reaction with diethyl phosphorochloridite (Cl–P(OEt)_2_), followed by oxidation to phosphonate (V). Although attempts to obtain 4-CF_3_-*β*-lactams with a longer phosphonated chain at C-3 were unsuccessful, we proceeded to investigate the antibacterial efficacy of phosphonated 4-CF_3_-*β*-lactams, using nonphosphonated 4-CF_3_-*β*-lactam **25** and nonfluorinated 4-*n*Pr-*β*-lactam **26** as references. Our study demonstrates the biological activity of phosphonated lactams. Selected compounds can affect the growth of clinically relevant bacteria. The promising preliminary antibacterial results, obtained using the diffusion disk method and further supported by MIC and *β*-lactamase inhibitor screening assays, identified compounds **11** and **16** as the most promising candidates in antimicrobial evaluation. These findings highlight the potential for further biological studies, including the investigation of antibacterial efficacy in *in vivo* studies.^75^ Moreover, bioinformatic structural analyses, including *in silico* molecular docking and molecular dynamic simulation to explore the interactions between the selected compounds and *β*-lactamase, are the subject of future research.^76,77^

## Supporting Information Available

Biological test figures and copies of ^1^H, ^13^C, ^19^F, ^31^P NMR, 2D NOESY, ^1^H–^1^H, and HOESY ^1^H–^19^F NMR spectra (PDF)
