# Supplementary material for: Synthesis and Antibacterial Activity of Novel Phosphonated CF3-β-lactams
Source: ACS Omega. 2025 Apr 28;10(17):18062–72. doi: 10.1021/acsomega.5c01562 (PMC12060044; doi:10.1021/acsomega.5c01562)
Supplement: Supplementary file 1 — ao5c01562_si_001.pdf [file ao5c01562_si_001.pdf]

Supporting Information

Synthesis and antibacterial activity of the novel phosphonated *β*-lactams

Monika Skibinska[1,3], Alicja Warowicka[2], Benoît Crousse*[3], Tomasz Cytlak*[1,4]

(1) Faculty of Chemistry, Adam Mickiewicz University, Uniwersytetu Poznańskiego 8, 61-614 Poznań, Poland,
(2) Faculty of Biology, Adam Mickiewicz University, Uniwersytetu Poznańskiego 6, 61-614 Poznań, Poland,
(3) BioCIS UMR 8076 CNRS, Univ. Paris-Saclay, Building Henri Moissan, 17 avenue des sciences, 91400 Orsay, France
(4) Center for Advanced Technologies, Adam Mickiewicz University, Uniwersytetu Poznańskiego 10, 61-614 Poznań, Poland

Corresponding authors: benoit.crousse@universite-paris-saclay.fr and cytlak@amu.edu.pl

Table of Contents

1. **Material and Methods……………………………………………………………………..S2**
2. **Disc diffusion assay figures……………………………….……………………………S16**
3. **Visual effect of evaluation of *β*-lactamase inhibition……………….…………………….S17**
4. **^{1}H, ^{13}C, ^{19}F, ^{31}P Spectra of phosphonated CF_3-*β*-lactams………….………………….…….S18**
5. **^{1}H, ^{13}C, ^{19}F Spectra of new 4-CF_3-*β*-lactams…………….…………………………..….S37**
6. **Examples of 2D NOESY ^{1}H-^{1}H and HOESY ^{1}H-^{19}F NMR spectra……………..…….….…....S42**
7. **Reactions of 3-Allyl, 3-Bn, 3-CO_2Et-4-CF_3-PMP (19-21) lactams with ClP(O)(OEt)$_2$……......S44**
8. **Cis/Trans isomerization process investigation based on NMR analysis of the crude reaction mixtures…………………………………………………………………………………S46**
9. **Attempt to C-3 methylation of 3-phosphonated 4-CF_3-*β*-lactam……………….……………...S52**
10. **References……………………………………………………………………….…S55**

1. MATERIALS AND METHODS

Experimental section

Antibacterial studies

To determine the antibacterial properties of synthetized compounds, bacterial strains *Staphylococcus aureus* (*S. aureus*, ATCC 25923), methicillin – resistant *Staphylococcus aureus* (MRSA, ATCC 43300), *Escherichia coli* (*E. coli*, ATCC 25922), *Neisseria gonorrhoeae* (ATCC 43069) were subjected to biological tests. All bacteria species bacterial strains were purchased from the Polish Collection of Microorganisms (Wrocław, Poland). Evaluation of bacteriostatic activities of compounds were carried out by the disc diffusion method and by the *β*-lactamase inhibition screening assay.

Disc diffusion assay

To evaluate the antibacterial activity of compounds, the disc diffusion assay was used. Compounds were dissolved in chloroform. Sterile paper discs (BioMaxima) were impregnated with compound, and the loaded filter paper discs were placed at precise locations on the prepared agar plates (TSA). The TSA plates containing filter paper discs loaded with various compounds were incubated at 37 °C for 24 h. After incubation, the inhibition zones were measured in millimeters (mean ± SD, n = 2). The results are presented in Table 2. The diameters of the zones were compared with those of standard antibiotics. Antibiotic discs (BioMaxima) used as a positive controls: TMP (Trimethoprin), RA (Rifampicin) and SXT (Sulphamethoxonazole/Trimethoprin).

Minimal Inhibitory Concentrations (MIC)

Synthesized compounds were tested for their antibacterial activity using the broth micro dilution method. Studied compounds were dissolved in DMSO to prepare the stock solutions. Compounds were evaluated in various concentrations ranging from 1000 μg/mL to 7,8 μg/mL were screened for measuring the minimal inhibitory concentration (MIC value). Bacteria strains were subcultured overnight at 37 °C in a Tripsin Soy Broth (TSB) with gentle shaking. After the incubation period, bacteria strains were suspended in TSB liquid medium to achieve a turbidity equivalent to a 0,5 McFarland standard. Then, this bacteria culture was diluted to 5 x 10^5 CFU/mL in fresh liquid TSB. Wells of sterile microtriter plates were filled with 100 μl of bacterial subculture. For measuring the MIC, studied compounds were serially diluted in microtriter plates starting from the

first well of plate until the last well (the lowest concentration). Wells without compounds (only broth was added to 100 µl of bacterial subculture) and bacteria treated with reference antibiotic (rifampicin) were served as a controls. Next, plates were covered with Parafilm membrane and incubated at 37°C for 10 - 12 h. MIC value was defined as the lowest concentration of compound at which the growth of bacteria is completely inhibited. The absorbance was adjusted/measured at 600 nm (OD 600 nm) by using a spectrophotometer (BioTek Synergy H1 Hybrid Plate Reader). MIC were performed according to reccomendation of EUCAST (European Committee on Antimicrobial Susceptibility Testing.

Evaluation of β-lactamase inhibition

In order to evaluate if our designed compounds **8**, **11**, **12**, **16** can inhibit bacterial β-lactamases, colorimetric *β*-lactamase Inhibitor Screening Kit (Merck, cat. no. MAK222) assay was applied following described protocol. In this convenient assay the activity of β-lactamase was measured spectrophotometrically. Briefly, equal amounts of each studied compound (125 µg in 20 µl of DMSO) were added separately to the wells of flat-bottom clear microplate for colorimetric assay (Greiner Bio-One). Then, 50 µl of Inhibition Reaction Mix (48 µl of β-lactamase assay buffer and 2 µl of β-lactamase) was added to each well. After incubation time (10 min., 25°C, in dark) 30 µl of Enzymatic Reaction Mix (1 µl of nitrocefin, a chromogenic substrate for β-lactamase enzyme and 29 µl of β-lactamase assay buffer) was added and mixed well. The final reaction volume in each well was 100 µl. Hydrolysis of nitrocefin by β-lactamase produces coloured product (light pink). Immediately after the addition of nitrocefin, the absorbance value was determined/recorded at 490 nm using a microplate reading spectrophotometer in kinetic mode (Synergy H1 Hybrid Reader, BioTek Instruments, Inc.). The measurement was recorded every minute for 30 minutes at room temperature. The amount of produced color is directly proportional to the beta-lactamase (β-lactamase, βLac) enzyme activity. The reaction plate was photographed. For the inhibition assay, samples blank (omitting of β-lactamase in Inhibition Reaction Mix), enzyme control (uninhibited β-lactamase, β-lactamase assay buffer in place of sample inhibitor) and inhibitor control (clavulanic acid, an inhibitor of *β*-lactamase, in *β*-lactamase assay buffer) were added. All reactions were performed in triplicate.

General Methods: ^{1}H NMR, ^{13}C NMR, ^{19}F NMR and ^{31}P NMR spectra were performed on Bruker ASCEND 600 (600 MHz) Bruker ASCEND 400 (400 MHz) Bruker ASCEND 300 (300 MHz) spectrometers. Structural assignments were made with additional information from gCOSY, gHSQC, and gHMBC experiments. All 2D and 1D selective NMR spectra were recorded on Bruker ASCEND 600 (600 MHz) spectrometer. Chemical shifts of ^{1}H NMR were expressed in parts per million downfield from tetramethylsilane (TMS) as an internal standard ($\delta = 0$) in $CDCl_3$. Chemical shifts of ^{13}C NMR were expressed in parts per million downfield and upfield from $CDCl_3$ as an internal standard ($\delta = 77.0$). Chemical shifts of ^{19}F NMR were expressed in parts per million upfield from $CFCl_3$ as an internal standard ($\delta = 0$) in $CDCl_3$. All d.r. were evaluated on the basis of ^{19}F NMR reaction mixture. High-resolution mass spectra were recorded by electron spray (MS-ESI) techniques using QToF Impact HD Bruker spectrometer. Reagent grade chemicals were used. THF was dried by refluxing with sodium metal-benzophenone (THF) and distilled under argon atmosphere. All moisture sensitive reactions were carried out under argon atmosphere using ovendried glassware. Reaction temperatures below 0°C were performed using a cooling bath (liquid N_2/*n*-hexane or liquid N_2/*i*-PrOH). TLC was performed on Merck Kieselgel 60-F254 with EtOAc/*n*-hexane and MeOH/$CHCl_3$ as developing systems, and products were detected by inspection under UV light (254 nm) and with a solution of potassium permanganate. Merck Kieselgel 60 (0.063-0.200 μm), Merck Kieselgel 60 (0.040-0.063 μm), Merck Kieselgel 60 (0.015-0.004 μm), were used for column chromatography.

General procedure synthesis of 4-CF_3-*β*-lactams, substrates for phophonates synthesis

In a two-neck flask activated zinc (5 eq.) and the corresponding *α*-bromoester (3.5 eq.) has been placed. Then, an appropriate CF_3-imine (1 eq.) in dry THF were added under argon atmosphere. The suspension was slowly warmed until 50°C then to reflux. After 1 h, the mixture was cooled to room temperature and quenched with saturated NH_4Cl aq., then extracted with Et_2O (4 × 15 mL). The organic layer was dried using anhydrous $MgSO_4$ or Na_2SO_4 and filtered. Then, the solvent was concentrated under vacuum and the crude mixture was purified by column chromatography with silica gel (*n*-hexane/ethyl acetate 80:20 to 70:30 – depending on C-3 substituent of *β*-lactam).

Racemic mixture of (*S*)-1-benzyl-4-(trifluoromethyl)azetidin-2-one (rac 6)

The *N*-Bn 4-CF_3-*β*-lactam **6** was obtained as pale yellow oil (1.17 g, 68%). To the reaction *N*-Bn CF_3-imine (1.40 g, 7.49 mmol, 1 eq.), ethyl *α*-bromoacetate (2.91 mL, 26.20 mmol, 3.5 eq.) and zinc (2.43 g, 37.45 mmol, 5 eq.) were used, following the general procedure. The crude product

was purified *via* column chromatography, using mixture of *n*-hexane/ethyl acetate 80:20, v/v. The NMR data were in good agreement.[1]

Diagnostic signals: ^{1}H NMR (600 MHz, CDCl3): δ = 7.39 – 7.07 (m, 2H, Ar), 4.75 (d, *J* = 15.1 Hz, 1H, C*H*HPh), 3.92 (d, *J* = 15.1 Hz, 1H, CH*H*Ph), 3.74 (ddp, *J* = 8.4, 5.7, 2.9 Hz, 1H, C*H*CF_3), 3.04 (dd, *J* = 14.9, 5.1 Hz, 1H, C*H*HCHCF_3), 2.94 (dd, *J* = 14.9, 2.2 Hz, 1H, CH*H*CHCF_3) ppm. ^{19}F NMR (565 MHz, $CDCl_3$): δ = −75.30 (d, J = 5.7 Hz) ppm.

Racemic mixture of (*S*)-1-(4-methoxyphenyl)-4-(trifluoromethyl)azetidin-2-one (rac 9)

The *N*-PMP 4-CF_3-*β*-lactam **9** was obtained as pale yellow oil (1.53 g, 65%). To the reaction *N*-PMP CF_3-imine (1.95 g, 9.61 mmol, 1 eq.), ethyl *α*-bromoacetate (3.73 mL, 33.62 mmol, 3.5 eq.) and zinc (3.12 g, 48.05 mmol, 5 eq.) were used, following the general procedure. The crude product was purified *via* column chromatography, using mixture of *n*-hexane/ethyl acetate 80:20, v/v. The NMR data were in good agreement.[2]

Diagnostic signals: ^{1}H NMR (600 MHz, $CDCl_3$): δ = 7.37 – 7.26 (m, 2H, Ar), 6.91 – 6.81 (m, 2H, Ar), 4.48 (ddq, *J* = 8.2, 5.3, 2.7 Hz, C*H*CF_3), 3.76 (s, 3H, OCH_3), 3.28 (dd, *J* = 15.4, 5.6 Hz, 1H, C*H*HCHCF_3), 3.11 (dd, *J* = 15.4, 2.5 Hz, 1H, CH*H*CHCF_3) ppm. ^{19}F NMR (565 MHz, $CDCl_3$): δ = −74.25 (d, J = 5.5 Hz) ppm.

Racemic mixture of (*S*)-1-(4-methoxybenzyl)-4-(trifluoromethyl)azetidin-2-one (rac 10)

The *N*-PMB 4-CF_3-β-lactam **10** was obtained as pale yellow oil (1.00 g, 71%). To the reaction *N*-PMB CF_3-imine (1.18 g, 5.44 mmol), ethyl α-bromoacetate (2.11 mL, 19.04 mmol, 3.5 eq.) and zinc (1.77 g, 27.20 mmol, 5 eq.) were used, following the general procedure. The crude product was purified *via* column chromatography, using mixture of *n*-hexane/ethyl acetate 80:20, v/v. The NMR data were in good agreement.[3]

Diagnostic signals: ^{1}H NMR (600 MHz, $CDCl_3$): δ = 7.18 (d, *J* = 8.5 Hz, 2H, Ar), 6.88 (d, *J* = 8.6 Hz, 2H, Ar), 4.77 (d, *J* = 15.0 Hz, 1H, C*H*HAr), 3.92 (d, *J* = 15.0 Hz, 1H, CH*H*Ar), 3.84 – 3.76 (m, 4H, C*H*CF_3, OCH_3), 3.08 (dd, *J* =14.9, 5.3 Hz, 1H, C*H*HCHCF_3), 3.02 (dd, *J* = 14.9, 1.4 Hz, 1H, CH*H*CHCF_3) ppm. ^{19}F NMR (565 MHz, $CDCl_3$): δ = −75.29 (d, J = 5.8 Hz) ppm.

Racemic mixture of (3*S*,4*S*)-1-(4-methoxyphenyl)-3-methyl-4-(trifluoromethyl)azetidin-2-one (rac *cis*-13) and racemic mixture of (3*R*,4*S*)-1-(4-methoxyphenyl)-3-methyl-4-(trifluoromethyl)azetidin-2-one (rac *trans*-13)

The *cis/trans* *N*-PMP 4-CF_3-*β*-lactams **13** (0.7:1 ratio) were obtained as pale yellow oil (1.02 g, 74%). To the reaction *N*-PMP CF_3-imine (1.08 g, 5.32 mmol), ethyl *α*-bromopropanoate (2.42 mL, 18.62 mmol, 3.5 eq.) and zinc (1.73 g, 26.60 mmol, 5 eq.) were used, following the general procedure. The crude product was purified *via* column chromatography, using mixture of *n*-hexane/ethyl acetate 75:25, v/v. The NMR data were in good agreement.[3, 4]

Diagnostic signals of rac *cis*-13: ^{1}H NMR (600 MHz, $CDCl_3$): δ = 7.35 (d, J = 8.9 Hz, 2H, Ar), 6.88 (d, J = 9.0 Hz, 2H, Ar), 4.51 (q, J = 6.2 Hz, 1H, $CHCF_3$), 3.66 (p, J = 7.5 Hz, 1H, $CHCHCF_3$), 3.79 (s, OCH_3), 1.44 (d, J = 7.5 Hz, 3H, $CHCH_3$) ppm. ^{19}F NMR (565 MHz, $CDCl_3$): δ = −68.17 (d, J = 5.1 Hz) ppm.

Diagnostic signals of rac *trans*-13: ^{1}H NMR (600 MHz, $CDCl_3$): δ = 7.35 (d, *J* = 8.8 Hz, 2H, Ar), 6.89 (d, *J* = 9.0 Hz, 2H, Ar), 4.11 (m, $C\mathit{H}CF_3$), 3.42 (m, 1H, $C\mathit{H}CHCF_3$), 3.79 (s, $OC\mathit{H}_3$), 1.47 (d, *J* = 7.4 Hz, 3H, $CHC\mathit{H}_3$) ppm. ^{19}F NMR (565 MHz, $CDCl_3$): δ = −73.40 (d, *J* = 5.0 Hz) ppm.

Racemic mixture of (3*S*,4*S*)-1-(4-benzyl)-3-methyl-4-(trifluoromethyl)azetidin-2-one (rac *cis*-14) and racemic mixture of (3*R*,4*S*)-1-(4-benzyl)-3-methyl-4-(trifluoromethyl)azetidin-2-one (rac *trans*-14)

The *cis/trans N*-Bn 4-CF_3-*β*-lactams **14** (0.2:1 ratio). were obtained as yellow oil (391 mg, 51%). To the reaction *N*-Bn CF_3-imine (508 mg, 2.72 mmol), ethyl *α*-bromopropanoate (1.24 mL, 9.52 mmol, 3.5 eq.) and zinc (884 mg, 13.64 mmol, 5 eq.) were used following the general procedure. The crude product was purified *via* column chromatography, using mixture of *n*-hexane/ethyl acetate 75:25, v/v. The NMR data were in good agreement for **rac *trans*-14**.[1]

Rac *cis*-14: ^{1}H NMR (600 MHz, $CDCl_3$) δ = 7.37 – 7.24 (m, 5H, Ph), 4.84 (d, *J* = 15.1 Hz, 1H, C*H*HPh), 3.97 (d, *J* = 15.1 Hz, 1H, CH*H*Ph), 3.88 – 3.80 (m, 1H, $C\mathit{H}CF_3$), 3.48 – 3.42 (m, 1H, $C\mathit{H}CH_3$), 1.33 (dd, *J* = 7.6, 1.5 Hz, 3H, $C\mathit{H}_3$) ppm. ^{13}C NMR (151 MHz, $CDCl_3$) δ = 169.4 (s, N*C*=O), 134.8, 129.1, 128.6, 128.2 (4 x s, Ar), 124.7 (q, *J* = 280.4 Hz, $\mathit{C}F_3$), 54.0 (q, *J* = 32.8 Hz, $\mathit{C}HCF_3$), 47.4 (s, $\mathit{C}HCH_3$), 45.4 (s, $\mathit{C}H_2Ph$), 9.0 (s, $\mathit{C}H_3$) ppm. ^{19}F NMR (565 MHz, $CDCl_3$) δ = −69.25 (d, *J* = 6.7 Hz) ppm. HRMS (ESI) *m/z:* $[M + Na]^+$ Calcd for $C_{12}H_{12}F_3NO_2Na^+$ 282.0717; Found: 282.0725.

Diagnostic signals of rac *trans*-14: ^{1}H NMR (600 MHz, $CDCl_3$) δ = 7.40 – 7.27 (m, 5H, Ph), 4.87 (d, *J* = 15.1 Hz, 1H, C*H*HPh), 3.97 (d, *J* = 15.1 Hz, 1H, CH*H*Ph), 3.42 (dq, *J* = 6.0, 2.0 Hz, 1H, $C\mathit{H}CF_3$), 3.36 – 3.28 (m, 1H, $C\mathit{H}CH_3$), 1.33 (d, *J* = 7.4 Hz, 3H, $C\mathit{H}_3$) ppm. ^{19}F NMR (565 MHz, $CDCl_3$) δ = −74.59 (d, *J* = 5.9 Hz) ppm.

Racemic mixture of (3*S*,4*S*)-1-(4-methoxybenzyl)-3-methyl-4-(trifluoromethyl)azetidin-2-one (rac *cis*-15) and racemic mixture of (3*R*,4*S*)-1-(4-methoxybenzyl)-3-methyl-4-(trifluoromethyl)azetidin-2-one (rac *trans*-15)

The *cis/trans N*-PMB 4-CF_3-*β*-lactams **15** (0.5:1 ratio) were obtained as yellow oil (772 mg, 46%). To the reaction *N*-PMB CF_3-imine (543 mg, 2.5 mmol), ethyl *α*-bromopropanoate (1.14 mL, 8.75 mmol, 3.5 eq.) and zinc (810 mg, 12.5 mmol, 5 eq.) were used, following the general procedure. The crude product was purified *via* column chromatography, using mixture of *n*-hexane/ethyl acetate 75:25, v/v. The NMR data were in good agreement for **rac *cis*-15**.[4]

Diagnostic signals of rac *cis*-15: ^{1}H NMR (600 MHz, $CDCl_3$) δ = 7.17 (d, *J* = 8.5 Hz, 2H, Ar), 6.88 (d, *J* = 8.5 Hz, 2H, Ar), 4.78 (d, *J* = 15.0 Hz, 1H, C*H*HPh), 3.89 (d, *J* = 15.0 Hz, 1H, CH*H*Ph), 3.80 (m, 4H, OCH_3, C*H*CF_3), 3.43 (dq, *J* = 7.6, 5.5 Hz, 1H, C*H*CH_3), 1.34 (dd, *J* = 7.7, 1.3 Hz, 3H, CH_3) ppm. ^{19}F NMR (565 MHz, $CDCl_3$) δ = −69.24 (d, *J* = 6.7 Hz) ppm.

Rac *trans*-15: ^{1}H NMR (600 MHz, $CDCl_3$) δ = 7.17 (d, *J* = 8.5 Hz, 2H, Ar), 6.88 (d, *J* = 8.5 Hz, 2H, Ar), 4.79 (d, *J* = 15.0 Hz, 1H, C*H*HPh), 3.87 (d, *J* = 14.9 Hz, 1H, CH*H*Ph), 3.80 (s, 3H, OCH_3), 3.37 (dq, *J* = 6.0, 2.1 Hz, 1H, C*H*CF_3), 3.30 (dq, *J* = 7.4 Hz, 1H, C*H*CH_3), 1.29 (d, *J* = 7.4 Hz, 3H, CH_3) ppm. ^{13}C NMR (151 MHz, $CDCl_3$) δ = 169.2 (s, N*C*=O), 160.0 (s, Ar), 129.9 (s, Ar), 127.0 (s, Ar), 124.6 (q, *J* = 279.4 Hz, *C*F_3), 114.5 (s, Ar), 57.1 (q, *J* = 34.1 Hz, *C*HCF_3), 55.4 (s, O*C*H_3), 46.9 (br d, *J* = 1.3 Hz *C*HCH_3), 44.9 (s, *C*H_2Ph), 12.4 (s, *C*H_3) ppm. ^{19}F NMR (565 MHz, $CDCl_3$) δ = −74.58 (d, *J* = 5.3 Hz) ppm. HRMS (ESI) *m/z:* $[M + Na]^+$ Calcd for $C_{13}H_{14}F_3NO_2Na^+$ 296.0874; Found: 296.0877.

Racemic mixture of (3*S*,4*S*)-1-(4-methoxyphenyl)-3-ethyl-4-(trifluoromethyl)azetidin-2-one (rac *cis*-17) and racemic mixture of (3*R*,4*S*)-1-(4-methoxyphenyl)-3-ethyl-4-(trifluoromethyl)azetidin-2-one (rac *trans*-17)

The *cis/trans* *N*-PMP 4-CF_3-*β*-lactams **17** (0.4:1 ratio) were obtained as yellow oil (1.31 g, 70%). To the reaction *N*-PMP CF_3-imine (1.39 g, 6.85 mmol), ethyl *α*-bromobutanoate (3.54 mL, 23.97 mmol, 3.5 eq.) and zinc (2.22 g, 34.25 mmol, 5 eq.) were used, following the general procedure. The crude product was purified *via* column chromatography, using mixture of *n*-hexane/ethyl acetate 75:25, v/v. The NMR data were in good agreement.[5]

Diagnostic signals of rac *cis*-17: ^{1}H NMR (600 MHz, $CDCl_3$): δ = 7.35 (d, *J* = 7.9 Hz, 2H, Ar), 6.88 (d, *J* = 7.8 Hz 2H, Ar), 4.52 (m, 1H, C*H*CF_3), 3.79 (s, 3H, OCH_3), 3.47 (q, *J* = 5.6 Hz 1H, C*H*CHCF_3), 1.99 (dp, *J* =13.6, 6.3 Hz, 1H, CH*H*CH_3), 1.82 (m, 1H, C*H*HCH_3), 1.18 (t, *J* = 7.1 Hz, 3H, CH2C*H*$_3$) ppm. ^{19}F NMR (565 MHz, $CDCl_3$): δ = −68.31 (d, *J* = 5.2 Hz) ppm.

Diagnostic signals of rac *trans*-17: ^{1}H NMR (600 MHz, $CDCl_3$): δ = 7.38 – 7.33 (m, 2H, Ar), 6.91 – 6.86 (m, 2H, Ar), 4.15 (q, *J* = 5.4 Hz, 1H, C*H*CF_3), 3.79 (s, 3H, OCH_3), 3.33 (ddd, *J* = 8.4, 6.0, 2.2 Hz 1H, C*H*CHCF_3), 1.96 (dp, *J* = 14.1, 7.2 Hz, 1H, C*H*HCH_3), 1.80 (dp, *J* =15.0, 7.5 Hz, 1H, CH*H*CH_3), 1.10 (t, *J* = 7.4 Hz, 3H, CH_2CH_3) ppm. ^{19}F NMR (565 MHz, $CDCl_3$): δ = −73.13 (d, *J* = 5.4 Hz) ppm.

Racemic mixture of (3*S*,4*S*)-1-(4-methoxyphenyl)-3-phenyl-4-(trifluoromethyl)azetidin-2-one (rac *cis*-18) and racemic mixture of (3*R*,4*S*)-1-(4-methoxyphenyl)-3-phenyl-4-(trifluoromethyl)azetidin-2-one (rac *trans*-18)

The *cis/trans* *N*-PMP 4-CF_3-*β*-lactams **18** (0.2:1 ratio) were obtained as yellow oil (1.38 g, 77%). To the reaction *N*-PMP CF_3-imine (1.13 g, 5.57 mmol), ethyl *α*-bromo-*α*-phenylacetate (3.39 mL, 19.48 mmol, 3.5 eq.) and zinc (1.81 g, 27.85 mmol, 5 eq.) were used following the general procedure. The crude product was purified *via* column chromatography, using mixture of *n*-hexane/ethyl acetate 75:25, v/v. The NMR data were in good agreement.[6]

Racemic mixture of (3*R*,4*S*)-3-allyl-1-(4-methoxyphenyl)-4-(trifluoromethyl) azetidin-2-one (rac 27)

The racemic mixture of 3-allyl-4-CF_3-*β*-lactam **27** was obtained as pale yellow oil (37 mg, 26%) in the mixture together with 3,3-diallyl-4-CF_3-*β*-lactam. To the reaction *N*-PMP 4-CF_3-*β*-lactam (121 mg, 0.49 mmol, 1 eq.), allyl bromide (51 µL, 0.59 mmol, 1.2 eq.) and LiHMDS (0.73 mL, 0.73 mmol, 1.5 eq.) were used, following the general procedure. The crude product was purified *via* column chromatography, using mixture of *n*-hexane/ethyl acetate 80:20, v/v. The NMR data were in good agreement.[3]

Diagnostic signals: ^{1}H NMR (600 MHz, $CDCl_3$): δ = 7.34 (d, *J* = 7.34 Hz, 2H, Ar), 6.89 (d, *J* = 8.9 Hz, 2H, Ar), 5.83 – 5.76 (m, 1H, *H*C = CH_2), 5.23 (d, *J* = 17.0 Hz, 1H, HC = C*H*H), 5.18 (d, *J* = 10.2 Hz, 1H, HC = CH*H*), 4.20 (qd, *J* = 5.4, 1.6 Hz, 1H, C*H*CF_3), 3.80 (s, 3H, OCH_3), 3.47 (m, 1H, C*H*CHCF_3), 2.65 (dt, *J* = 12.8, 6.0 Hz, 1H, C*H*HCH = CH_2), 2.54 (dt, *J* = 15.0, 7.6 Hz, 1H, CH*H*CH = CH_2) ppm. ^{19}F NMR (565 MHz, $CDCl_3$): δ = −73.29 (d, *J* = 4.9 Hz) ppm.

Racemic mixture of (3R,4S)-3-benzyl-1-(4-methoxyphenyl)-4-(trifluoromethyl)azetidin-2-one (rac 28)

The racemic mixture of 3-Bn-4-CF_3-*β*-lactam **28** was obtained as pale yellow oil (113 mg, 56%). To the reaction *N*-PMP 4-CF_3-*β*-lactam (148 mg, 0.60 mmol, 1 eq.), benzyl bromide (64 µL, 0.54 mmol, 0.9 eq.) and LiHMDS (0.90 mL, 0.90 mmol, 1.5 eq.) were used, following the general procedure. The crude product was purified *via* column chromatography, using mixture of *n*-hexane/ethyl acetate 80:20, v/v.

^{1}H NMR (400 MHz, $CDCl_3$): δ = 7.28 – 7.13 (m, 7H, CH_2Ph, Ar) 6.80 – 6.74 (m, 2H, Ar), 4.05 (qd, *J* = 5.5, 2.2 Hz, 1H, C*H*CF_3), 3.71 (s, 3H, OCH_3), 3.62 – 3.58 (m, 1H, C*H*CHCF_3), 3.13 (dd, *J* = 13.5, 6.8 Hz, 1H, C*H*HPh), 3.02 (dd, *J* = 14.7, 7.6 Hz, 1H, CH*H*Ph) ppm. ^{13}C NMR (101 MHz, $CDCl_3$): δ = 165.5 (s, N*C* = O), 157.3 (s, Ar), 136.4 (s, CH_2Ph), 129.7 (s, CH_2Ph), 129.1 (s, CH_2Ph), 129.0 (s, CH_2Ph), 127.4 (s, Ar), 124.3 (q, *J* = 280.8 Hz, *C*F_3), 120.0 (m, Ar), 114.5 (s, Ar), 56.2 (q, *J* = 34.5 Hz, *C*HCF_3), 55.6 (s, OCH_3), 53.0 (s, *C*CHCF_3), 33.6 (s, *C*H_2Ph) ppm. ^{19}F NMR (376 MHz, $CDCl_3$): δ = −73.05 (d, *J* = 5.4 Hz) ppm. HRMS (ESI) *m/z:* $[M + Na]^+$ Calcd for $C_{18}H_{16}F_3NO_2Na^+$ 358.1025; Found: 358.1027.

Racemic mixture of ethyl (3R,4S)-1-(4-methoxyphenyl)-2-oxo-4- (trifluoromethyl)azetidine-3-carboxylate (rac 29)

The racemic mixture of ethyl 3-carboxylate-4-CF_3-*β*-lactam **29** was obtained as pale yellow oil (75 mg, 47%). To the reaction *N*-PMP 4-CF_3-*β*-lactam (123 mg, 0.50 mmol, 1 eq.), ethyl chloroformate (57 µL, 0.60 mmol, 1.2 eq.) and LiHMDS (0.75 mL, 0.75 mmol, 1.5 eq.) were uszed, following the general procedure. The crude product was purified *via* column chromatography, using mixture of *n*-hexane/ethyl acetate 80:20, v/v. The NMR data were in good agreement.[6]

General procedure synthesis of phosphonated 4-CF_3-*β*-lactams with diethyl chlorophosphate (Cl-P(O)$(OEt)_2$)

In a round-bottom flask, dry THF (2 mL) was cooled to −25°C, then LiHMDS (3 eq., 1 M in THF) was added dropwise under an argon atmosphere. Next, the solution of the corresponding *β*-lactam (1 eq.) in dry THF (1 mL) was added. The suspension was stirred at the same temperature for 30 min. and then diethyl chlorophosphate (6 eq.) was added dropwise. Then, the reaction mixture was stirred at the same temperature for 2 h and left overnight at room temperature. Afterwards, the reaction mixture was cooled to 0°C and carefully quenched by dropwise addition of saturated NH_4Cl aq. (1 mL), and extracted with Et_2O (2 × 5 mL). The organic layers were washed with brine, dried over anhydrous $MgSO_4$ or Na_2SO_4, filtrated, and concentrated under reduced pressure. The crude product(s) was/were purified using column chromatography (*n*-hexane/ethyl acetate or cyclohexane/ethyl acetate).

Racemic mixture of diethyl ((3R,4R)-1-benzyl-2-oxo-4-(trifluoromethyl)azetidin-3-yl)phosphonate (*rac* 8)

The racemic mixture of **8** was obtained as a pale yellow oil (74 mg, 67%). To the reaction *N*-Bn-4-CF_3-*β*-lactam (69 mg, 0.3 mmol, 1 eq.), diethyl chlorophosphate (0.26 mL, 1.81 mmol, 6 eq.) and LiHMDS (0.9 mL, 0.9 mmol, 3 eq.) were used, following the general procedure. The crude product was purified *via* column chromatography, using mixture of *n*-hexane/ethyl acetate 75:25, v/v.

^{1}H NMR (600 MHz, $CDCl_3$): δ = 7.39 – 7.30 (m, 5H, Ph), 4.90 (d, *J* = 15.3 Hz, 1H, C*H*HPh), 4.22 – 4.12 (m, 4H, 2 × OC$H_2$$CH_3$), 4.04 – 4.00 (m, 2H, C*H*CF_3, CH*H*Ph), 3.67 (dd, *J* = 15.4, 2.2 Hz, 1H, C*H*CHCF_3), 1.31 (t, *J* = 7.0 Hz, 6H, 2 × OCH_2CH_3) ppm. ^{13}C NMR (151 MHz, $CDCl_3$): δ = 160.7 (d, *J* = 6.5 Hz, N*C* = O), 133.9, 129.1, 128.6, 128.5 (4 × s, Ar), 123.9 (dq, *J* = 280.0, 3.7 Hz, *C*F_3), 63.5 (d, *J* = 6.2 Hz, O*C*$H_2$$CH_3$), 63.3 (d, *J* = 6.6 Hz, O*C*$H_2$$CH_3$), 51.7 (m, *C*H$CF_3$), 49.3 (d, *J* = 149.1 Hz, *C*HCHCF_3), 46.2 (m, *C*H_2Ph), 16.5 (d, *J* = 1.4 Hz, OCH_2*C*H_3), 16.4 (d, *J* = 1.5 Hz, OCH_2*C*H_3) ppm. ^{19}F NMR (565 MHz, $CDCl_3$): δ = −74.80 (d, *J* = 5.5 Hz) ppm. ^{19}F {/^{1}H} NMR (565 MHz, $CDCl_3$): δ = −74.80 (s) ppm. ^{31}P {/^{1}H} NMR (565 MHz, $CDCl_3$): δ = 16.22 (s) ppm. HRMS (ESI) *m/z:* $[M + Na]^+$ Calcd $C_{15}H_{19}F_3NO_4PNa^+$ 388.0901; Found: 388.0902.

Racemic mixture of diethyl ((3R,4R)-1-(4-methoxyphenyl)-2-oxo-4-(trifluoromethyl)azetidin-3-yl)phosphonate (*rac* 11)

The racemic mixture of **11** was obtained as a pale yellow oil (112 mg, 90%). To the reaction *N*-PMP-4-CF_3-*β*-lactam (80 mg, 0.33 mmol, 1 eq.), diethyl chlorophosphate (0.142 mL, 0.99 mmol, 3 eq.) and LiHMDS (0.99 mL, 0.99 mmol, 3 eq.) were used, following the general procedure. The

crude product was purified *via* column chromatography, using mixture of *n*-hexane/ethyl acetate 55:45, v/v.

^{1}H NMR (600 MHz, $CDCl_3$): δ = 7.34 (d, *J* = 7.9 Hz, 2H, Ar), 6.89 (d, *J* = 7.6 Hz, 2H, Ar), 4.69 – 4.66 (m, 1H, C*H*CF_3), 4.32 – 4.19 (m, 4H, 2 × OCH_2CH_3), 3.79 – 3.75 (m, 4H, C*H*CHCF_3, OCH_3), 1.37 – 1.33 (m, 6H, 2 × OCH_2CH_3) ppm. ^{13}C NMR (151 MHz, $CDCl_3$): δ = 157.9 (d, *J* = 6.2 Hz, N*C* = O), 157.6, 129.6 (2 × s, Ar), 123.8 (dq, *J* = 280.6, 3.7 Hz, *C*F_3), 120.1, 114.6 (2 × s, Ar), 63.9 (d, *J* = 6.2 Hz, OCH_2CH_3), 63.4 (d, *J* = 6.6 Hz, OCH_2CH_3), 55.6 (s, OCH_3), 53.3 (qd, *J* = 35.8, 2.1 Hz, *C*HCF_3), 49.3 (d, *J* = 148.1 Hz, *C*HCHCF_3), 16.5 (d, *J* = 3.3 Hz, O$CH_2$$CH_3$), 16.4 (d, *J* = 3.2 Hz, O$CH_2$$CH_3$) ppm. ^{19}F NMR (565 MHz, $CDCl_3$): δ = −73.71 (d, *J* = 5.1 Hz) ppm. ^{19}F {/^{1}H} NMR (565 MHz, $CDCl_3$): δ = −73.71 (s) ppm. ^{31}P {/^{1}H} NMR (243 MHz, $CDCl_3$): δ = 15.84 (s) ppm. HRMS (ESI) *m/z:* $[M + Na]^+$ Calcd $C_{15}H_{19}F_3NO_5PNa^+$ 404.0851; Found: 404.0861.

Racemic mixture of diethyl ((3R,4R)-1-(4-methoxybenzyl)-2-oxo-4-(trifluoromethyl)azetidin-3-yl)phosphonate (*rac* 12)

The racemic mixture of **12** was obtained as a pale yellow oil (82 mg, 71%). To the reaction *N*-PMB-4-CF_3-*β*-lactam (79 mg, 0.3 mmol, 1 eq.), diethyl chlorophosphate (0.13 mL, 0.9 mmol, 3 eq.) and LiHMDS (0.9 mL, 0.9 mmol, 3 eq.) were used, following the general procedure. The crude product was purified *via* column chromatography, using mixture of *n*-hexane/ethyl acetate 65:35, v/v.

^{1}H NMR (400 MHz, $CDCl_3$): δ = 7.22 (d, *J* = 8.5 Hz, 2H, Ar), 6.89 (d, *J* = 8.5 Hz, 2H, Ar), 4.84 (d, *J* = 15.1 Hz, 1H, C*H*HPh), 4.22 – 4.11 (m, 4H, 2 × OCH_2CH_3), 4.02 – 3.92 (m, 2H, C*H*CF_3, CH*H*Ph), 3.80 (s, 3H, OCH_3), 3.65 (dd, *J* = 15.5, 2.5 Hz, 1H, C*H*CHCF_3), 1.33 – 1.29 (m, 6H, 2 × OCH_2CH_3) ppm. ^{13}C NMR (151 MHz, $CDCl_3$): δ = 160.6 (d, *J* = 6.4 Hz, N*C* = O), 159.7, 130.0, (2 × s, Ar), 126.7 (s, Ar), 123.9 (dq, *J* = 280.1, 3.8 Hz, *C*F_3), 114.5 (s, Ar), 63.5 (d, *J* = 6.2 Hz, OCH_2CH_3), 63.2 (d, *J* = 6.5 Hz, OCH_2CH_3), 55.4 (s, OCH_3), 51.5 (dq, *J* = 35.5, 1.6 Hz, *C*HCF_3), 49.1 (d, *J* = 148.8 Hz, *C*HCHCF_3), 45.6 (d, *J* = 1.3 Hz, *C*H_2Ph), 16.5 (d, *J* = 2.5 Hz, O$CH_2$$CH_3$), 16.4 (d, *J* = 2.6 Hz, O$CH_2$$CH_3$) ppm. ^{19}F NMR (376 MHz, $CDCl_3$): δ = −74.81 (d, *J* = 5.5 Hz) ppm. ^{19}F {/^{1}H} NMR (376 MHz, $CDCl_3$): δ = −74.81 (s) ppm. ^{31}P {/^{1}H} NMR (243 MHz, $CDCl_3$): δ = 16.34 (s) ppm. HRMS (ESI) *m/z:* $[M + Na]^+$ Calcd $C_{16}H_{21}F_3NO_5PNa^+$ 418.1007; Found: 418.1023.

Racemic mixture of diethyl ((3R,4R)-1-(4-methoxyphenyl)-3-methyl-2-oxo-4-(trifluoromethyl) azetidin-3-yl)phosphonate (*rac* 16)

The racemic mixture of **16** was obtained as a pale yellow oil (83 mg, 77%). To the reaction the mixture of *cis/trans* *N*-PMP-3-Me-4-CF_3-*β*-lactam (74 mg, 0.28 mmol, 1 eq.), diethyl chlorophosphate (0.123 mL, 0.86 mmol, 3 eq.) and LiHMDS (0.86 mL, 0.86 mmol, 3 eq.) were

used, following the general procedure. The crude product was purified *via* column chromatography, using mixture of *n*-hexane/ethyl acetate 60:40, v/v.

^{1}H NMR (600 MHz, $CDCl_3$): δ = 7.33 (d, *J* = 8.9 Hz, 2H, Ar), 6.87 (d, *J* = 8.9 Hz, 2H, Ar), 4.77 – 4.73 (m, 1H, C*H*CF_3), 4.28 – 4.16 (m, 4H, 2 × OC$H_2$$CH_3$), 3.78 (s, 3H, OC$H_3$), 1.67 (d, *J* = 16.9 Hz, 3H, CCH_3) 1.34 – 1.29 (m, 6H, 2 × OCH_2CH_3) ppm. ^{13}C NMR (151 MHz, $CDCl_3$): δ = 162.9 (d, *J* = 5.8 Hz, N*C* = O), 157.5 (s, Ar), 129.19 (d, *J* = 1.32 Hz, Ar), 123.8 (dq, *J* = 281.4, 4.3 Hz, *C*F_3), 120.6 (s, Ar), 114.4 (s, Ar), 63.8 (d, *J* = 6.5 Hz, O*C*$H_2$$CH_3$), 63.4 (d, *J* = 6.9 Hz, O*C*$H_2$$CH_3$), 57.4 – 56.0 (m, *C*H$CF_3$, *C*CH$CF_3$), 55.6 (s, O*C*$H_3$), 16.5 – 16.4 (m, 2 × O$CH_2$*C*$H_3$), 11.3 (s, C*C*$H_3$) ppm. ^{19}F NMR (565 MHz, $CDCl_3$): δ = −67.82 (d, *J* = 5.2 Hz) ppm. ^{19}F {/^{1}H} NMR (565 MHz, $CDCl_3$): δ = −67.82 (s) ppm. ^{31}P {/^{1}H} NMR (243 MHz, $CDCl_3$): δ = 20.78 (s) ppm. HRMS (ESI) *m/z:* $[M + Na]^+$ Calcd $C_{16}H_{21}F_3NO_5PNa^+$ 418.1007; Found: 418.1009.

Procedure for the C-3 methylation of 3-phosphonated 4-CF_3-*β*-lactam

In a round-bottom flask, dry THF (2 mL) was cooled to −25°C, and then LiHMDS (0.4 mL, 0.4 mmol, 3 eq., 1 M in THF) was added dropwise under an argon atmosphere. Next, the solution of the corresponding β-lactam **11** (45 mg, 0.14 mmol, 1 eq.) in dry THF (1 mL) was added. The suspension was stirred at the same temperature for 30 min. and then the CH_3I (8.4 μL, 0.21 mmol, 1.5 eq.) was added dropwise. Then, the reaction mixture was stirred at the same temperature for 2 h and left overnight at room temperature. Afterwards, the reaction mixture was cooled to 0°C and carefully quenched by dropwise addition of saturated NH_4Cl aq. (1 mL), and extracted with Et_2O (2×5 mL). The organic layers were washed with brine, dried over anhydrous $MgSO_4$ or Na_2SO_4, filtrated, and concentrated under reduced pressure. The crude product(s) were purified using column chromatography (n-hexane/ethyl acetate), but without receiving sufficiently pure product **16'**.

Racemic mixture of diethyl ((3S,4R)-1-(4-methoxyphenyl)-3-methyl-2-oxo-4-(trifluoromethyl) azetidin-3-yl)phosphonate (*rac* 16')

Diagnostic signals of rac 16': ^{19}F NMR (377 MHz, $CDCl_3$): δ = −65.91 (d, *J* = 5.4 Hz) ppm. ^{19}F {/^{1}H} NMR (377 MHz, $CDCl_3$): δ = −65.91 (s) ppm. ^{31}P {/^{1}H} NMR (162 MHz, $CDCl_3$): δ = 16.52 (s) ppm. GCMS *m/z:* 395.2

General procedure synthesis of phosphonated 4-CF_3-*β*-lactams with diethyl phosphorochloridite (Cl-P$(OEt)_2$)

In a round-bottom flask, dry THF (2 mL) was cooled to −25°C, then LiHMDS (3 eq., 1 M in THF) was added dropwise under an argon atmosphere. Next, the solution of the corresponding *β*-lactam

(1 eq.) in dry THF (1 mL) was added. The suspension was stirred at the same temperature for 1 h, then the reaction mixture was allowed to warm to -5 °C over the course of 30 min and then diethyl phosphorochloridite (1.2 eq.) was added dropwise. The resulting mixture again was cooled to -25 °C and stirred at the same temperature for 2 h and allowed to warm to room temperature over the course of 1 h Afterwards, the reaction mixture was cooled to -5 °C and then quenched by slow addition of hydrogen peroxide (30%, 10 eq.), and the resulting mixture was stirred vigorously at this temperature for 10 min. The resulting mixture was extracted with Et_2O (3 × 5 mL) and then the organic layers were washed with brine, dried over anhydrous $MgSO_4$ or Na_2SO_4, filtrated, and concentrated under reduced pressure. The crude product(s) was/were purified using column chromatography (*n*-hexane/ethyl acetate or cyclohexane/ethyl acetate).

Racemic mixture of diethyl ((3R,4R)-1-(4-methoxyphenyl)-2-oxo-4-(trifluoromethyl)azetidin-3-yl)phosphonate (*rac* 11)

The racemic mixture of **11** was obtained as a pale yellow oil (74 mg, 97%). To the reaction *N*-PMP-4-CF_3-*β*-lactam (50 mg, 0.2 mmol, 1 eq.), diethyl phosphorochloridite (36 μL, 0.24 mmol, 1.2 eq.), hydrogen peroxide (30%, 0.204 mL, 2 mmol, 10 eq.) and LiHMDS (0.4 mL, 0.4 mmol, 2 eq.) were used, following the general procedure. The crude product was purified *via* column chromatography, using mixture of *n*-hexane/ethyl acetate 55:45, v/v.

The NMR and HRMS data were in good agreement with above reported data (first methodology)

Racemic mixture of diethyl ((3R,4R)-1-(4-methoxyphenyl)-3-methyl-2-oxo-4-(trifluoromethyl) azetidin-3-yl)phosphonate (*rac* 16)

The racemic mixture of **16** was obtained as a pale yellow oil (72 mg, 92%). To the reaction the mixture of *cis/trans* *N*-PMP-3-Me-4-CF_3-*β*-lactam (55 mg, 0.2 mmol, 1 eq.), diethyl phosphorochloridite (36 μL, 0.24 mmol, 1.2 eq.), hydrogen peroxide (30%, 0.204 mL, 2 mmol, 10 eq.) and LiHMDS (0.4 mL, 0.4 mmol, 2 eq.) were used, following the general procedure. The crude product was purified *via* column chromatography, using mixture of *n*-hexane/ethyl acetate 60:40, v/v.

The NMR and HRMS data were in good agreement with above reported data (first methodology)

Racemic mixture of diethyl ((3R,4R)-3-ethyl-1-(4-methoxyphenyl)-2-oxo-4-(trifluoromethyl)azetidin-3-yl)phosphonate (*rac* 20*)*

The racemic mixture of **20** was obtained as a pale yellow oil (56 mg, 68%). To the reaction the mixture of *cis/trans* *N*-PMP-3-Et-4-CF_3-*β*-lactam (55 mg, 0.2 mmol, 1 eq.), diethyl phosphorochloridite (36 μL, 0.24 mmol, 1.2 eq.), hydrogen peroxide (30%, 0.204 mL, 2 mmol, 10 eq.) and LiHMDS (0.4 mL, 0.4 mmol, 2 eq.) were used, following the general procedure. The

crude product was purified *via* column chromatography, using mixture of *n*-hexane/ethyl acetate 45:55, v/v.

^{1}H NMR (600 MHz, $CDCl_3$): δ = 7.36 – 7.31 (m, 2H, Ar), 6.92 – 6.86 (m, 2H, Ar), 4.80 (dq, *J* = 9.2, 6.5 Hz, 1H, C*H*CF_3), 4.29 – 4.15 (m, 4H, 2 × OCH_2CH_3), 3.80 (s, 3H, OCH_3), 2.30 – 2.20 (m, 1H, CC*H*HCH_3), 2.19 – 2.09 (m, 1H, CC*H*HCH_3), 1.35 – 1.29 (m, 9H, 2 × OCH_2CH_3, CCH_2CH_3) ppm. ^{13}C NMR (151 MHz, $CDCl_3$): δ = 162.6 (d, *J* = 5.7 Hz, N*C* = O), 157.5 (s, Ar), 129.2 (d, *J* = 1.8 Hz, Ar), 123.9 (dq, *J* = 281.7, 4.0 Hz, *C*F_3), 120.8, 114.4 (2 × s, Ar), 63.6 (d, *J* = 6.6 Hz, O*C*H_2CH_3), 63.3 (d, *J* = 6.9 Hz, O*C*H_2CH_3), 57.7 (q, *J* = 33.8 Hz, *C*HCF_3), 55.5 (s, *C*CHCF_3), 20.3 (s, CCH_2*C*H_3), 16.5 – 16.3 (m, OCH_2*C*H_3), 10.1 (s, CCH_2*C*H_3) ppm. ^{19}F NMR (565 MHz, $CDCl_3$): δ = −66.95 (d, *J* = 6.5 Hz). ^{19}F {/^{1}H} NMR (565 MHz, $CDCl_3$): δ = −66.95 (s) ppm. ^{31}P {/^{1}H} NMR (243 MHz, $CDCl_3$): δ = 20.76 (s). HRMS (ESI) *m/z:* $[M + Na]^+$ Calcd $C_{17}H_{23}F_3NO_5PNa^+$ 432.1164; Found: 432.1170.

Racemic mixture of diethyl ((3R,4R)-1-(4-methoxyphenyl)-2-oxo-3-phenyl-4-(trifluoromethyl)azetidin-3-yl)phosphonate (rac 21)

The racemic mixture of **21** was obtained as a pale yellow oil (57 mg, 62%). To the reaction the mixture of *cis/trans* *N*-PMP-3-Ph-4-CF_3-*β*-lactam (64 mg, 0.2 mmol, 1 eq.), diethyl phosphorochloridite (36 μL, 0.24 mmol, 1.2 eq.), hydrogen peroxide (30%, 0.204 mL, 2 mmol, 10 eq.) and LiHMDS (0.4 mL, 0.4 mmol, 2 eq.) were used, following the general procedure. The crude product was purified *via* column chromatography, using mixture of *n*-hexane/ethyl acetate 50:50, v/v.

^{1}H NMR (600 MHz, $CDCl_3$): δ = 7.80 (d, *J* = 7.2 Hz, 2H, Ph), 7.41 – 7.37 (m, 5H, Ar, Ph), 6.93 – 6.90 (m, 2H, Ar), 5.00 (dq, *J* = 11.3, 5.6 Hz, 1H, C*H*CF_3), 4.26 – 4.19 (m, 1H, OC*H*HCH_3), 4.17 – 4.10 (m, 1H, OC*H*HCH_3), 4.01 – 3.94 (m, 1H, OCH*H*CH_3), 3.80 (s, 3H, OCH_3), 3.77 – 3.73 (m, 1H, OCH*H*CH_3), 1.20 (t, *J* = 7.1 Hz, 3H, OCH_2CH_3), 1.06 (t, *J* = 7.1 Hz, 3H, OCH_2CH_3) ppm. ^{13}C NMR (151 MHz, $CDCl_3$): δ = 161.8 (d, *J* = 7.9 Hz, N*C* = O), 157.9 (s, Ar), 129.2 (d, *J* = 1.0 Hz, Ar), 129.0 (d, *J* = 2.5 Hz, Ph), 128.9 (d, *J* = 5.9 Hz, Ph), 128.7 (d, *J* = 1.9 Hz, Ph), 128.2 (d, *J* = 4.6 Hz, Ph), 123.3 (dq, *J* = 281.4, 4.2 Hz, *C*F_3), 121.3, 114.6 (2 × s, Ar), 65.1 (d, *J* = 7.3 Hz, O*C*H_2CH_3), 64.8 (d, *J* = 137.3 Hz, *C*CHCF_3), 63.9 (d, *J* = 7.4 Hz, O*C*H_2CH_3), 59.0 (q, *J* = 33.0 Hz, *C*HCF_3), 55.6 (s, O*C*H_3), 16.2 (d, *J* = 6.3 Hz, OCH_2*C*H_3), 16.0 (d, *J* = 5.7 Hz, OCH_2*C*H_3) ppm. ^{19}F NMR (565 MHz, $CDCl_3$): δ = −68.32 (d, *J* = 5.5 Hz). ^{19}F {/^{1}H} NMR (565 MHz, $CDCl_3$): δ = −68.32 (s) ppm. ^{31}P {/^{1}H} NMR (243 MHz, $CDCl_3$): δ = 17.25 (s) ppm. HRMS (ESI) *m/z:* $[M + Na]^+$ Calcd $C_{21}H_{23}F_3NO_5PNa^+$ 480.1164; Found: 480.1175.

Racemic mixture of diethyl ((3R,4R)-3-allyl-1-(4-methoxyphenyl)-2-oxo-4-(trifluoromethyl)azetidin-3-yl)phosphonate (*rac* 22)

The racemic mixture of **22** was obtained as a pale yellow oil (51 mg, 60%). To the reaction the *trans N*-PMP-3-allyl-4-CF_3-*β*-lactam (58 mg, 0.2 mmol, 1 eq.), diethyl phosphorochloridite (36 μL, 0.24 mmol, 1.2 eq.), hydrogen peroxide (30%, 0.204 mL, 2 mmol, 10 eq.) and LiHMDS (0.4 mL, 0.4 mmol, 2 eq.) were used, following the general procedure. The crude product was purified *via* column chromatography, using mixture of *n*-hexane/ethyl acetate 40:60, v/v.

^{1}H NMR (600 MHz, $CDCl_3$): δ = 7.35 – 7.32 (m, 2H, Ar), 6.90 – 6.87 (m, 2H, Ar), 6.09 (ddt, *J* = 17.0, 10.0, 7.0 Hz, 1H, C*H*=CH_2), 5.23 – 5.16 (m, 2H, CH=CH_2), 4.81 (dq, *J* = 9.2, 6.5 Hz, 1H, C*H*CF_3), 4.26 – 4.14 (m, 4H, 2 × OC$H_2$$CH_3$), 3.79 (s, 3H, OC$H_3$), 3.00 – 2.84 (m, 2H, C$H_2$CH=$CH_2$), 1.32 and 1.30 (2 × t, *J* = 7.0 Hz, 6H, 2 × OCH_2CH_3) ppm. ^{13}C NMR (101 MHz, $CDCl_3$): δ = 162.2 (d, *J* = 5.7 Hz, N*C* = O), 157.7 (s, Ar), 132.0 (d, *J* = 3.4 Hz, *C*H=CH_2) 129.2 (d, *J* = 1.9 Hz, Ar), 123.9 (dq, *J* = 282.0 Hz *C*F_3, Ar), 120.9 (s, Ar), 118.7 (s, CH=*C*H_2), 114.6 (s, Ar), 63.9 (d, *J* = 6.6 Hz, O*C*$H_2$$CH_3$), 63.6 (d, *J* = 7.0 Hz, O*C*$H_2$$CH_3$), 60.3 (d, *J* = 141.6 Hz, *C*CHCF_3), 57.6 (q, *J* = 34.2 Hz, *C*HCF_3), 55.6 (s, O*C*H_3), 31.4 (br t, *J* = 2.0 Hz, *C*CH=CH_2), 16.5 (br s, OCH_2*C*H_3), 16.4 (br s, OCH_2*C*H_3) ppm. ^{19}F NMR (565 MHz, $CDCl_3$): δ = −66.71 (d, *J* = 6.4 Hz). ^{19}F {/^{1}H} NMR (565 MHz, $CDCl_3$): δ = −66.71 (s) ppm. ^{31}P {/^{1}H} NMR (565 MHz, $CDCl_3$): δ = 19.91 (s) ppm. HRMS (ESI) *m/z:* $[M + Na]^+$ Calcd $C_{18}H_{23}F_3NO_5PNa^+$ 444.1164; Found: 444.1174.

Racemic mixture of diethyl ((3*R*,4*R*)-1-benzyl-3-methyl-2-oxo-4-(trifluoromethyl)azetidin-3-yl)phosphonate (*rac* 23)

The racemic mixture of **23** was obtained as a pale yellow oil (18 mg, 74%). To the reaction the mixture of *cis/trans N*-Bn-3-Me-4-CF_3-*β*-lactam (15 mg, 0.06 mmol, 1 eq.), diethyl phosphorochloridite (11 μL, 0.072 mmol, 1.2 eq.), hydrogen peroxide (30%, 61 μL, 0.6 mmol, 10 eq.) and LiHMDS (0.12 mL, 0.12 mmol, 2 eq.) were used, following the general procedure. The crude product was purified *via* column chromatography, using mixture of *n*-hexane/ethyl acetate 75:25, v/v.

^{1}H NMR (600 MHz, $CDCl_3$): δ = 7.38 – 7.30 (m, 5H, Ph), 4.88 (d, *J* = 15.3 Hz, 2H, C*H*HPh), 4.21 – 4.09 (m, 5H, 2 × OC$H_2$$CH_3$, C*H*$CF_3$), 4.02 (d, *J* = 15.3 Hz, 2H, CH*H*Ph), 1.60 (dd, *J* = 17.0, 1.6 Hz, 3H, CCH_3) 1.30 (2 × t, *J* = 7.1 Hz, 6H, 2 × OCH_2CH_3) ppm. ^{13}C NMR (151 MHz, $CDCl_3$): δ = 165.3 (d, *J* = 5.8 Hz, N*C* = O), 134.0, 129.0, 128.7, 128.3 (4 x s, Ph), 123.9 (dq, *J* = 281.2, 4.0 Hz, *C*F_3), 63.7 (d, *J* = 6.5 Hz, O*C*$H_2$$CH_3$), 63.2 (d, *J* = 7.1 Hz, O*C*$H_2$$CH_3$), 56.5 (d, *J* = 144.7 Hz, *C*CHCF_3), 55.5 (q, *J* = 33.7 Hz, *C*HCF_3), 45.9 (d, *J* = 2.0 Hz *C*H_2Ph), 16.5 (d, *J* = 2.5 Hz, OCH_2*C*H_3), 16.4 (d, *J* = 2.4 Hz, OCH_2*C*H_3), 11.2 (br t, *J* = 2.4 Hz, C*C*H_3) ppm. ^{19}F NMR (565 MHz, $CDCl_3$): δ = −68.63 (d, *J* = 6.7 Hz). ^{19}F {/^{1}H} NMR (565 MHz, $CDCl_3$): δ = −68.63 (s) ppm. ^{31}P {/^{1}H} NMR (243 MHz, $CDCl_3$): δ = 21.14 (s). HRMS (ESI) *m/z:* $[M + Na]^+$ Calcd $C_{16}H_{21}F_3NO_4PNa^+$ 402.1058; Found: 402.1060.

Racemic mixture of diethyl ((3R,4R)-1-(4-methoxybenzyl)-3-methyl-2-oxo-4-(trifluoromethyl)azetidin-3-yl)phosphonate (*rac* 24)

The racemic mixture of **24** was obtained as a pale yellow oil (10 mg, 52%). To the reaction the mixture of *cis/trans* *N*-Bn-3-Me-4-CF_3-*β*-lactam (13 mg, 0.05 mmol, 1 eq.), diethyl phosphorochloridite (9 μL, 0.06 mmol, 1.2 eq.), hydrogen peroxide (30%, 51 μL, 0.5 mmol, 10 eq.) and LiHMDS (0.1 mL, 0.1 mmol, 2 eq.) were used, following the general procedure. The crude product was purified *via* column chromatography, using mixture of *n*-hexane/ethyl acetate 65:35, v/v.

^{1}H NMR (600 MHz, $CDCl_3$): δ = 7.26 – 7.25 (m, 2H, Ar), 6.89 (d, *J* = 8.6 Hz, 2H, Ar), 4.83 (d, *J* = 15.2 Hz, 2H, C*H*HPh), 4.20 – 4.09 (m, 5H, 2 × OC$H_2$$CH_3$, C*H*$CF_3$), 3.95 (d, *J* = 15.0 Hz, 2H, CH*H*Ph), 3.80 (s, 3H, OCH_3), 1.59 (dd, *J* = 17.0, 1.6 Hz, 3H, CCH_3) 1.32 (2 × t, *J* = 6.8 Hz, 6H, 2 × OCH_2CH_3) ppm. ^{13}C NMR (151 MHz, $CDCl_3$): δ = 165.3 (d, *J* = 5.9 Hz, N*C* = O), 159.6, 130.1, 125.9 (3 × s, Ar), 124.0 (dq, *J* = 281.0, 4.2 Hz, *C*F_3), 114.3 (s, Ar), 63.7 (d, *J* = 6.5 Hz, O*C*$H_2$$CH_3$), 63.2 (d, *J* = 7.0 Hz, O*C*$H_2$$CH_3$), 56.5 (d, *J* = 144.6 Hz, *C*HCHCF_3), 55.6 – 55.0 (m, *C*HCF_3, O*C*H_3), 45.3 (d, *J* = 2.0 Hz *C*H_2Ph), 16.5 (d, *J* = 2.7 Hz, OCH_2*C*H_3), 16.4 (d, *J* = 3.1 Hz, OCH_2*C*H_3), 11.2 (br s, C*C*H_3) ppm.^{19}F NMR (565 MHz, $CDCl_3$): δ = −68.64 (d, *J* = 6.7 Hz). ^{19}F {/^{1}H} NMR (565 MHz, $CDCl_3$): δ = −68.64 (s) ppm. ^{31}P {/^{1}H} NMR (243 MHz, $CDCl_3$): δ = 21.24 (s). HRMS (ESI) *m/z:* $[M + Na]^+$ Calcd $C_{17}H_{23}F_3NO_5PNa^+$ 432.1163; Found: 432.1168.

2. Disc diffusion assay figures

A B C

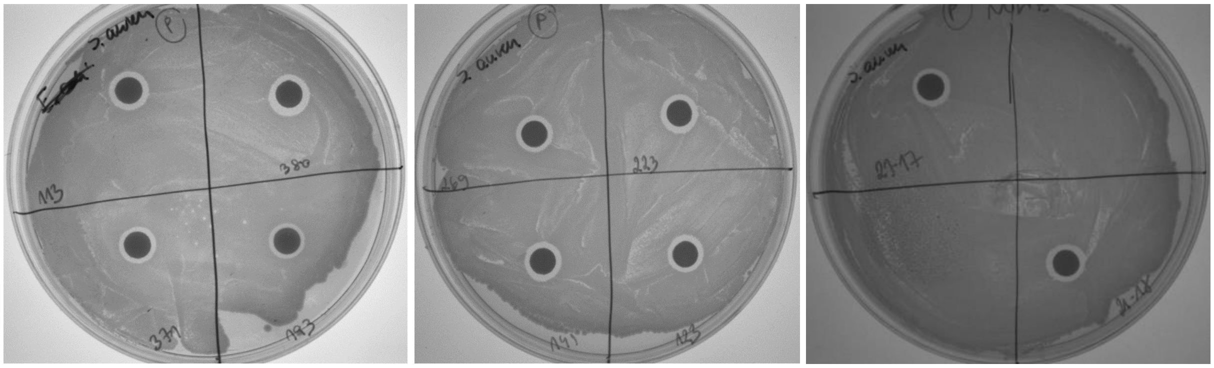

Figure S1. Antimicrobial activities of studied compounds:**8**, **11**, **12**, **16**, **18**, **20-24**, evaluated by the disk diffusion assay. Inhibition zones against *S. aureus* (ATCC 25923)

A B C

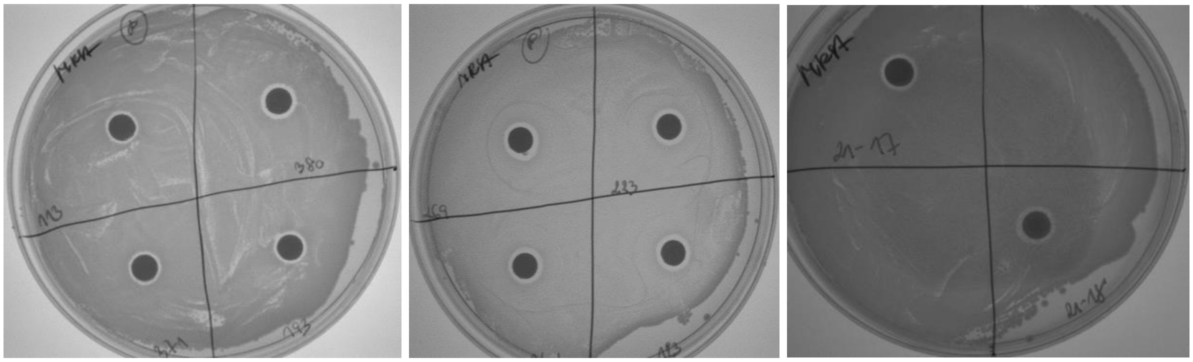

Figure S2. Antimicrobial activities of studied compounds: **8**, **11**, **12**, **16**, **18**, **20-24**, evaluated by the disk diffusion assay. Inhibition zones against D. MRSA (ATCC 43300)

A B C

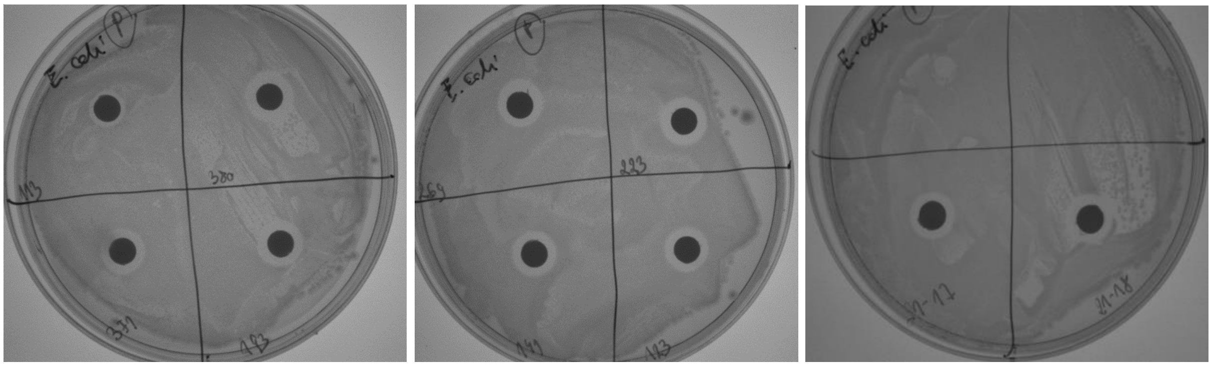

Figure S3. Antimicrobial activities of studied compounds: **8**, **11**, **12**, **16**, **18**, **20-24**, evaluated by the disk diffusion assay. Inhibition zones against *E. coli* (ATCC 25922)

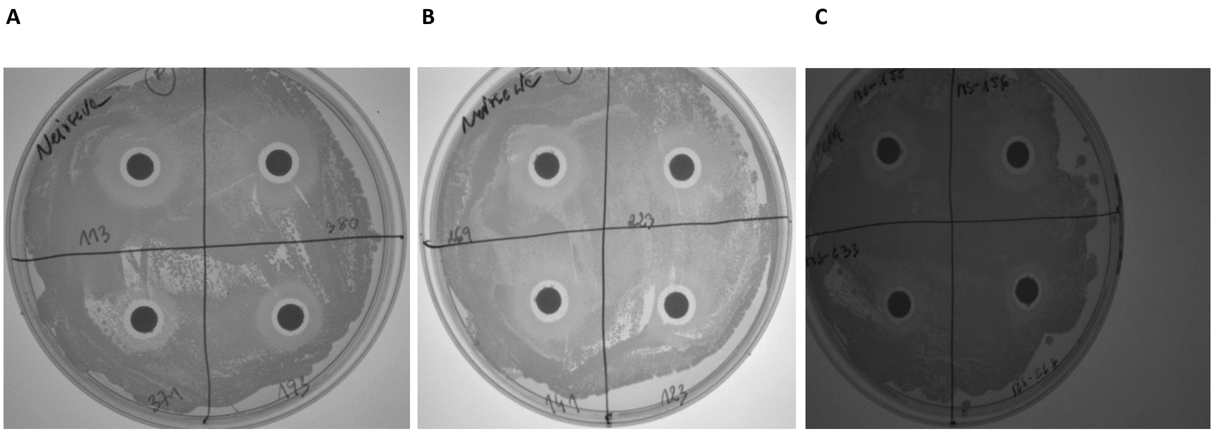

Figure S4. Antimicrobial activities of studied compounds: **8**, **11**, **12**, **16**, **18**, **20-22** and **25** (C, left bottom) evaluated by the disk diffusion assay. Inhibition zones against *N. gonorrhoeae* (ATCC 43069)

3. Visual effect of evaluation of β-lactamase inhibition

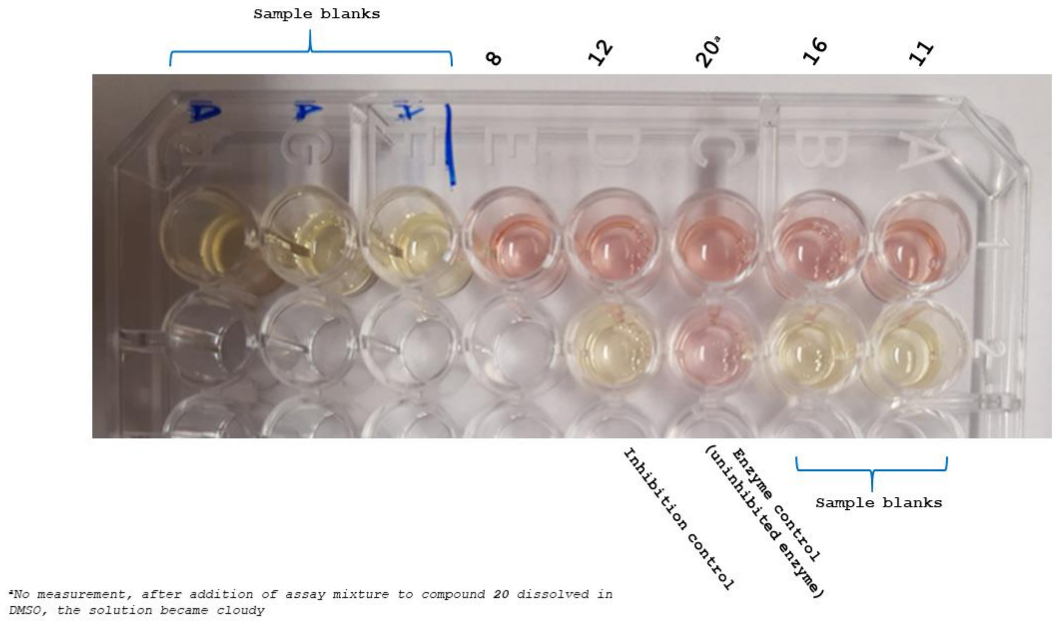

Figure S5. The visual effect of *β*-lactamase activity (hydrolysis a chromogen nitrocefin, producing a coloured product). Pink colour in well indicates hydrolysis of nitrocefin, a substrat for *β*-lactamase. Due to degradation (hydrolysis) nitrocefin changes colour from yellow to light pink

4. ^{1}H, ^{13}C, ^{19}F and ^{31}P NMR Spectra of phosphonated 4-CF_3-*β*-lactams

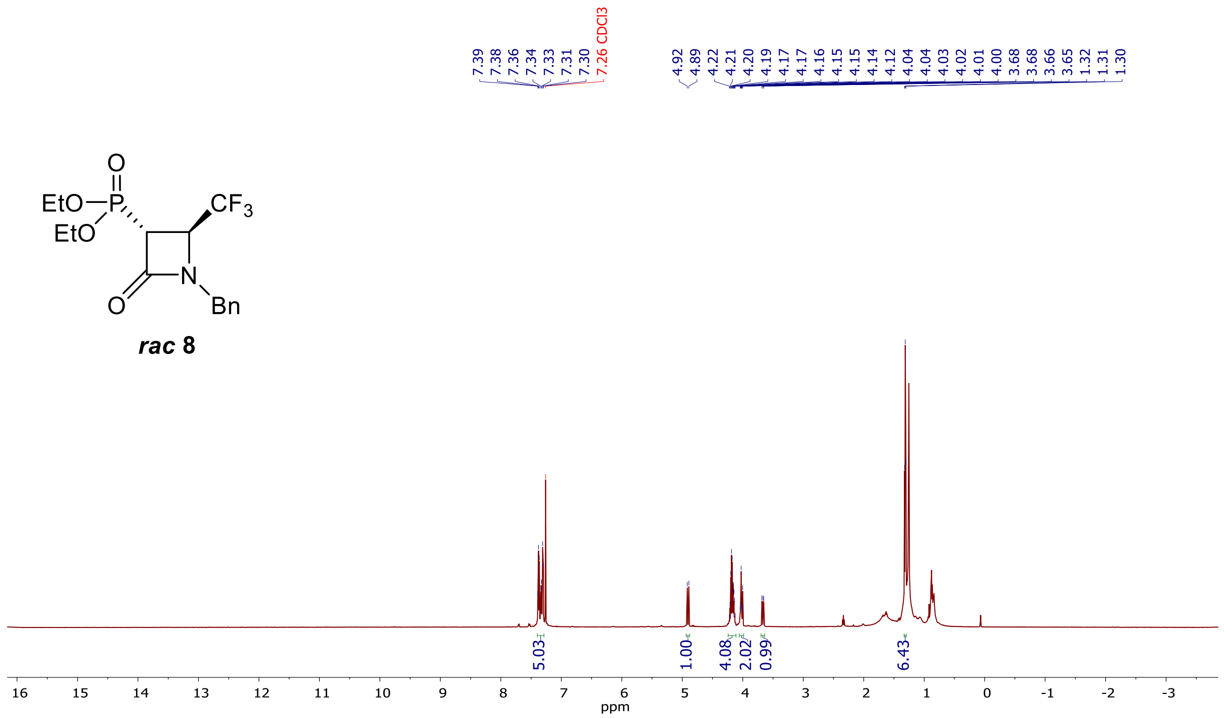

Figure S6. Spectrum ^{1}H NMR ($CDCl_3$, 600 MHz) of *rac* **8**

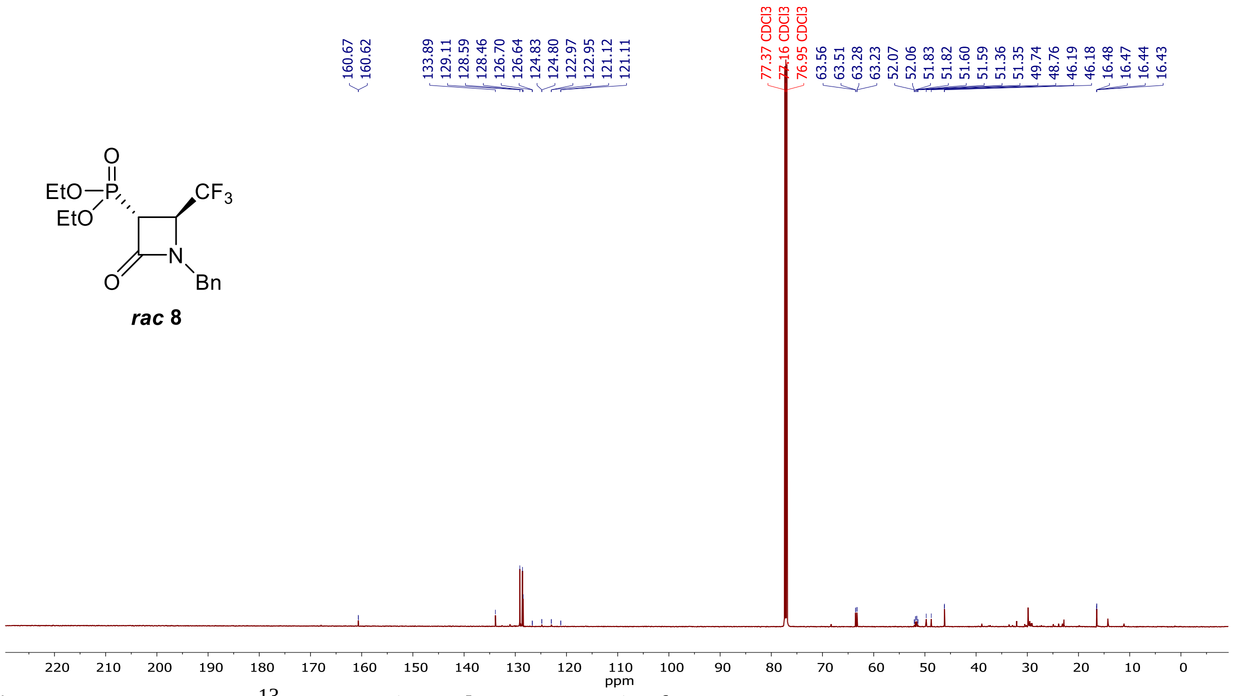

Figure S7. Spectrum ^{13}C NMR ($CDCl_3$, 151 MHz) of *rac* **8**

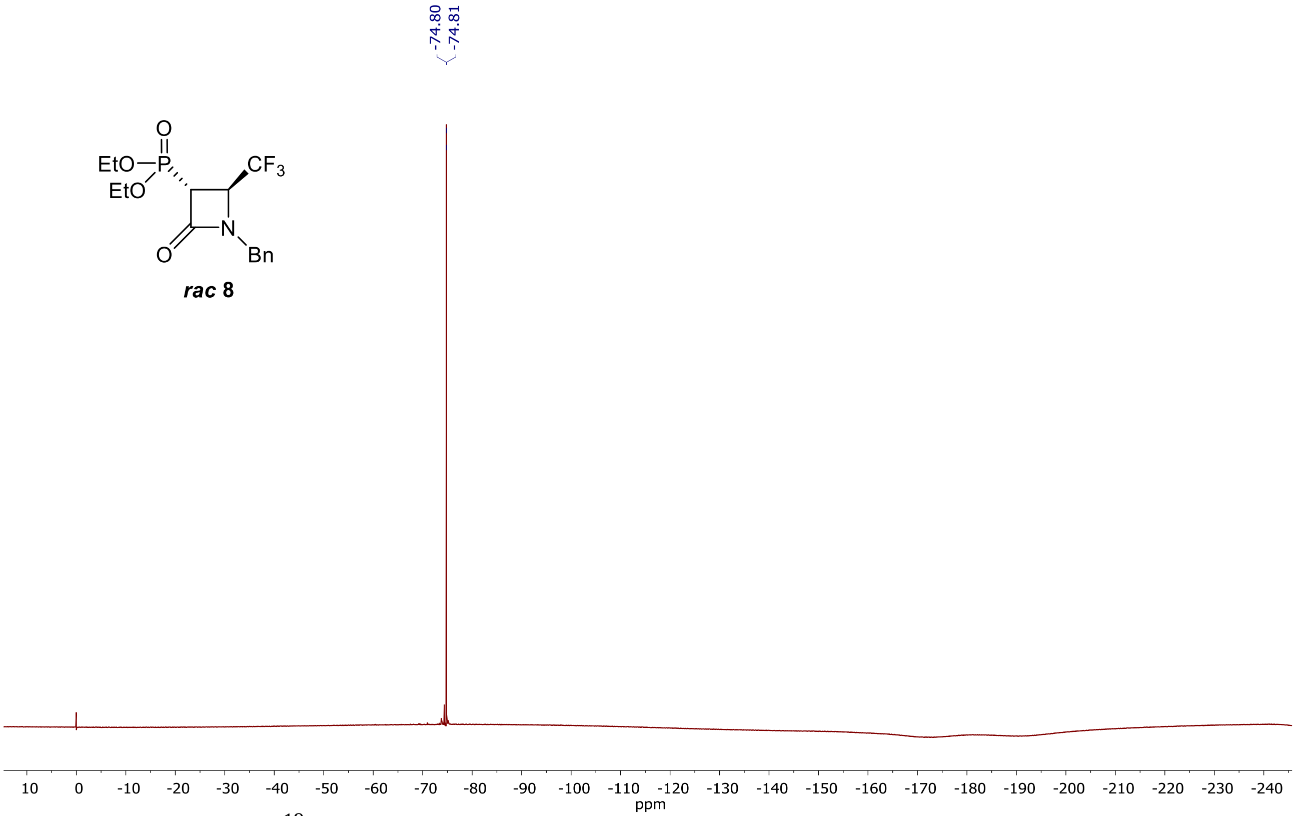

Figure S8. Spectrum ^{19}F NMR ($CDCl_3$, 565 MHz) of *rac* **8**

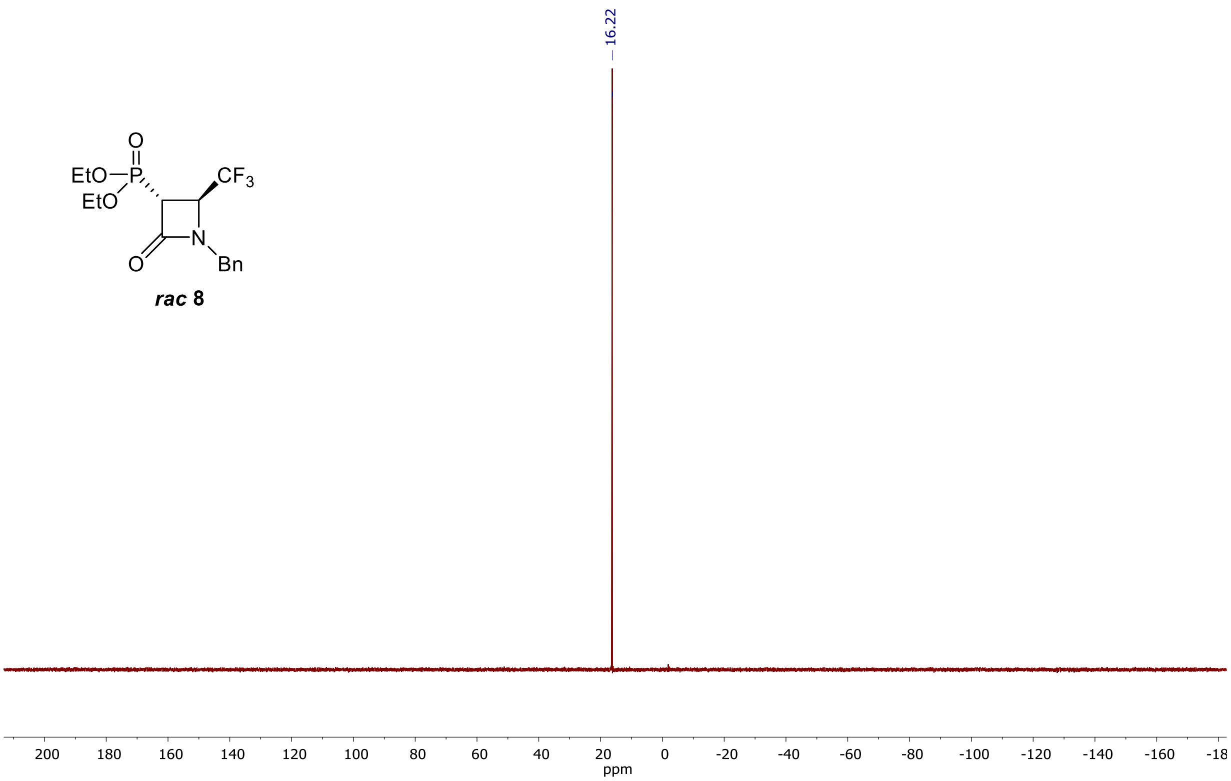

Figure S9. Spectrum ^{31}P NMR ($CDCl_3$, 243 MHz) of *rac* **8**

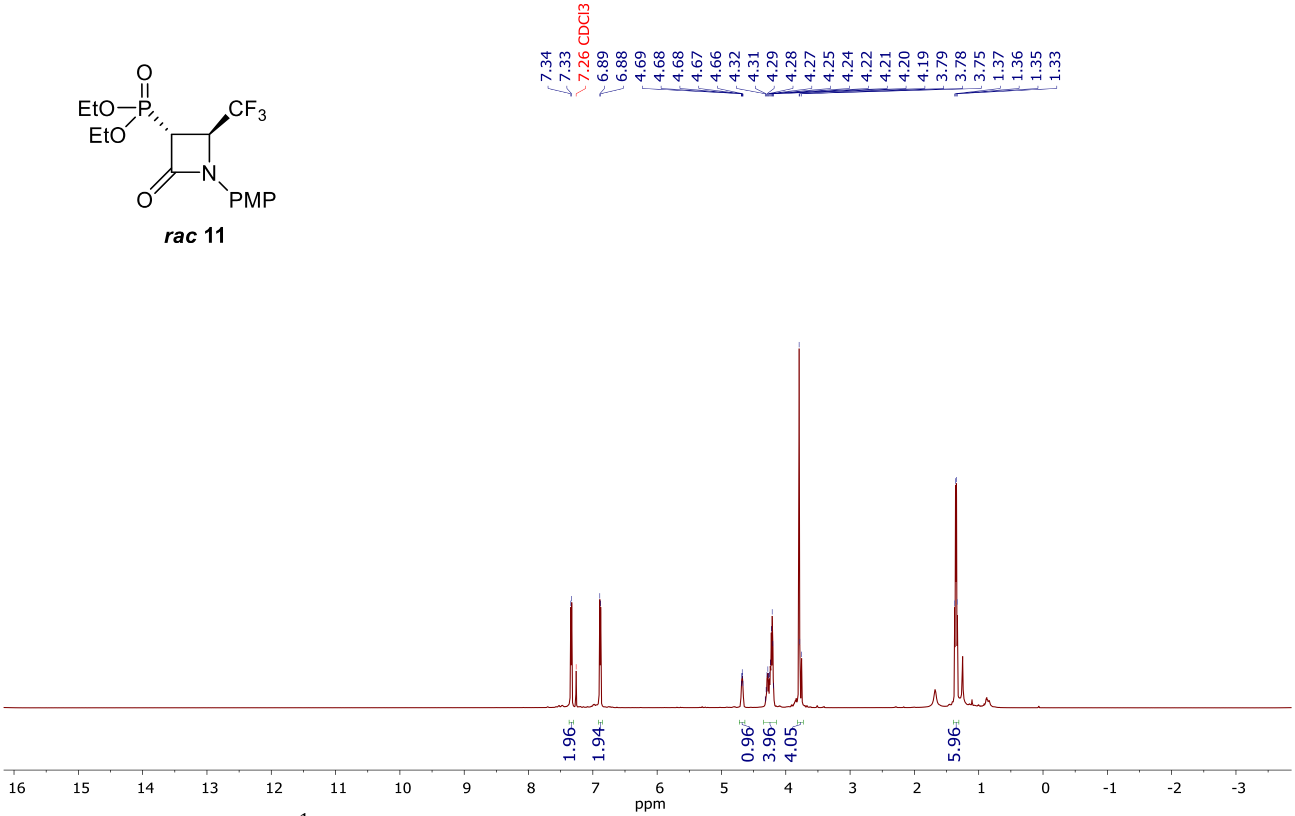

Figure S10. Spectrum ^{1}H NMR ($CDCl_3$, 600 MHz) of *rac* **11**

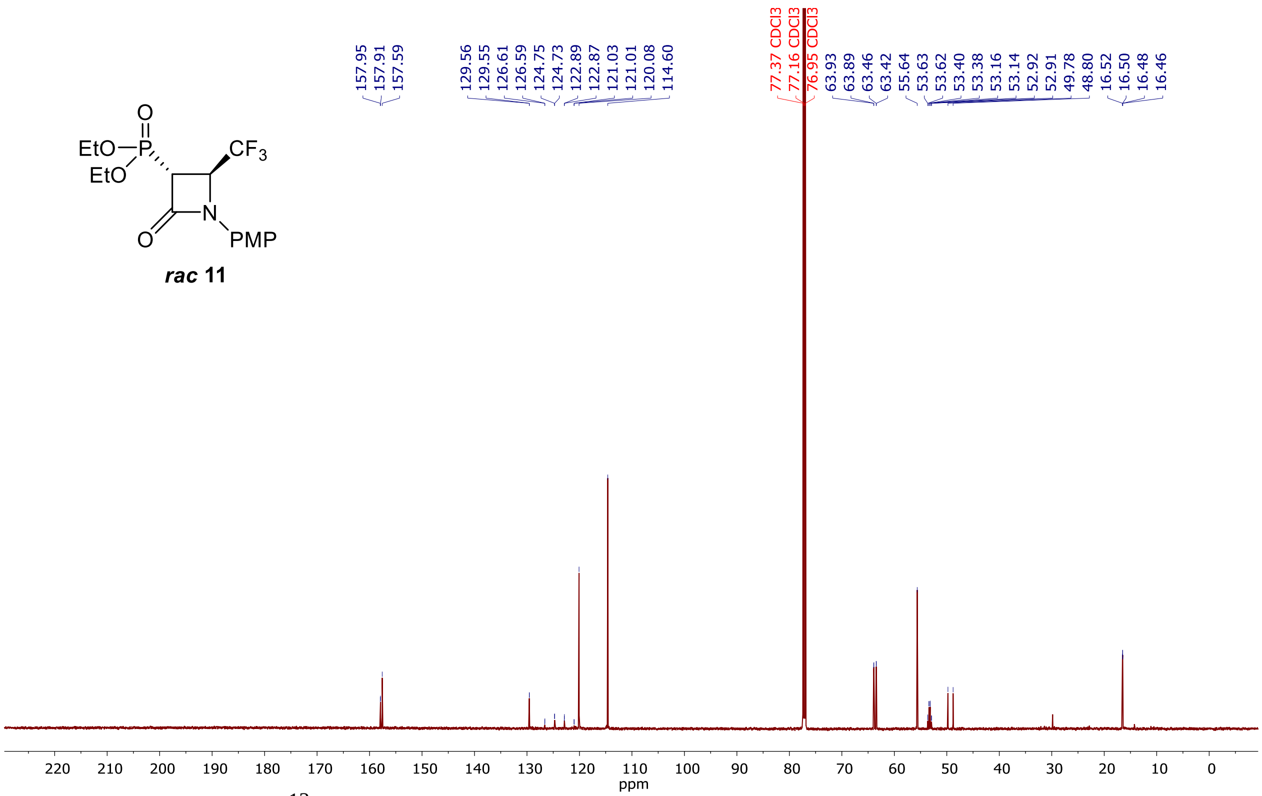

Figure S11. Spectrum ^{13}C NMR ($CDCl_3$, 151 MHz) of *rac* **11**

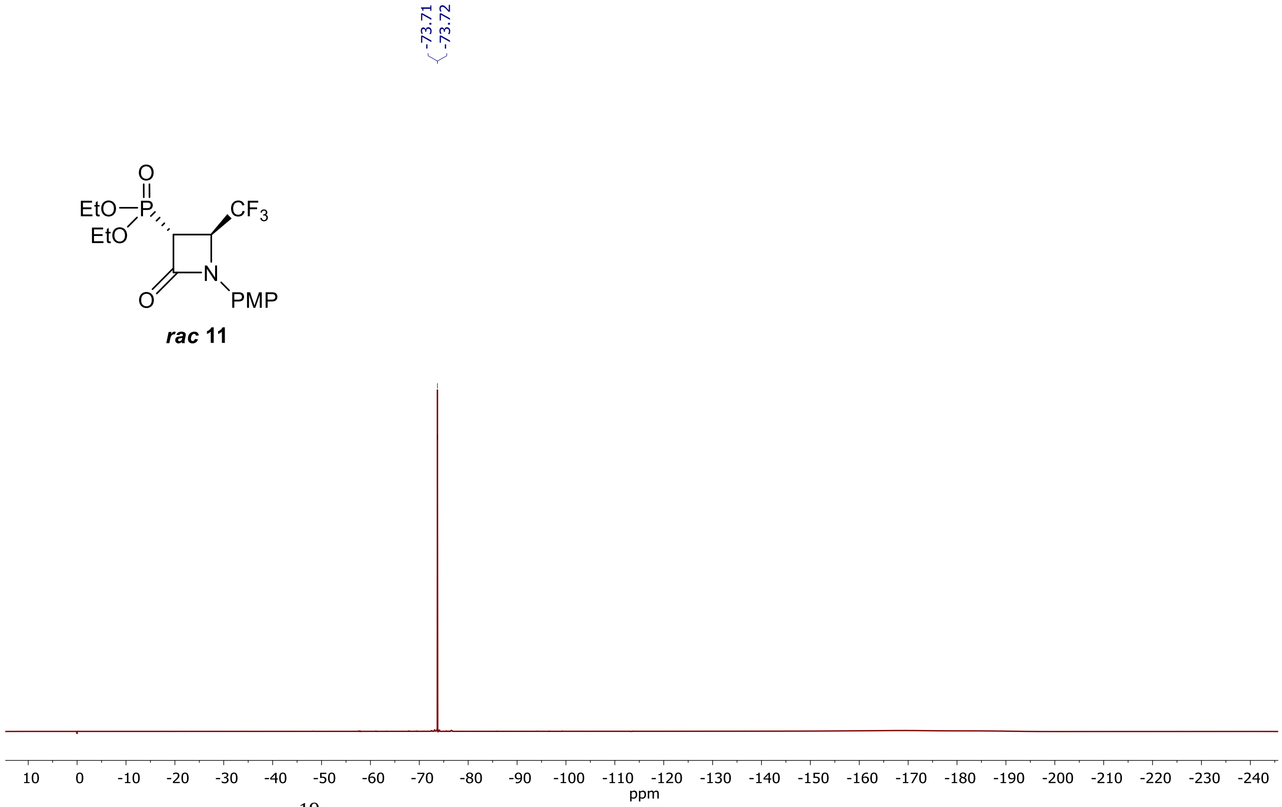

Figure S12. Spectrum ^{19}F NMR ($CDCl_3$, 565 MHz) of *rac* **11**

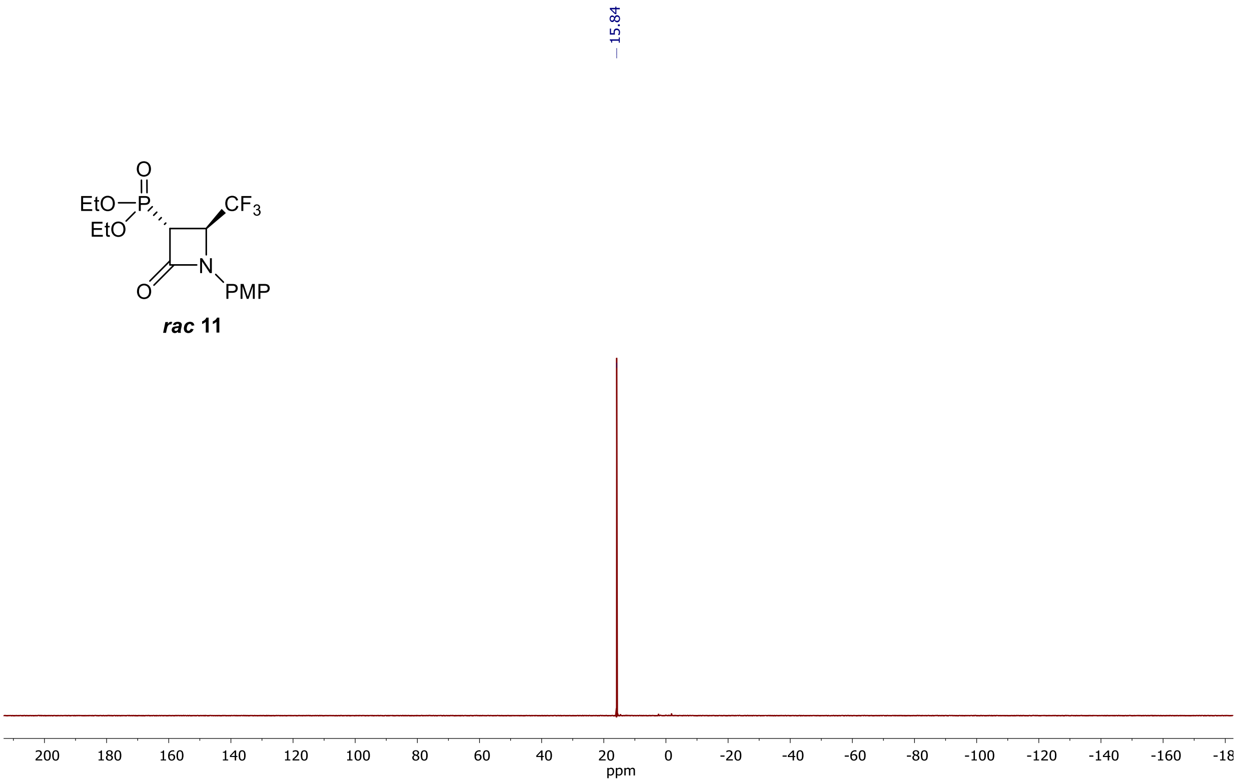

Figure S13. Spectrum ^{31}P NMR ($CDCl_3$, 243 MHz) of *rac* **11**

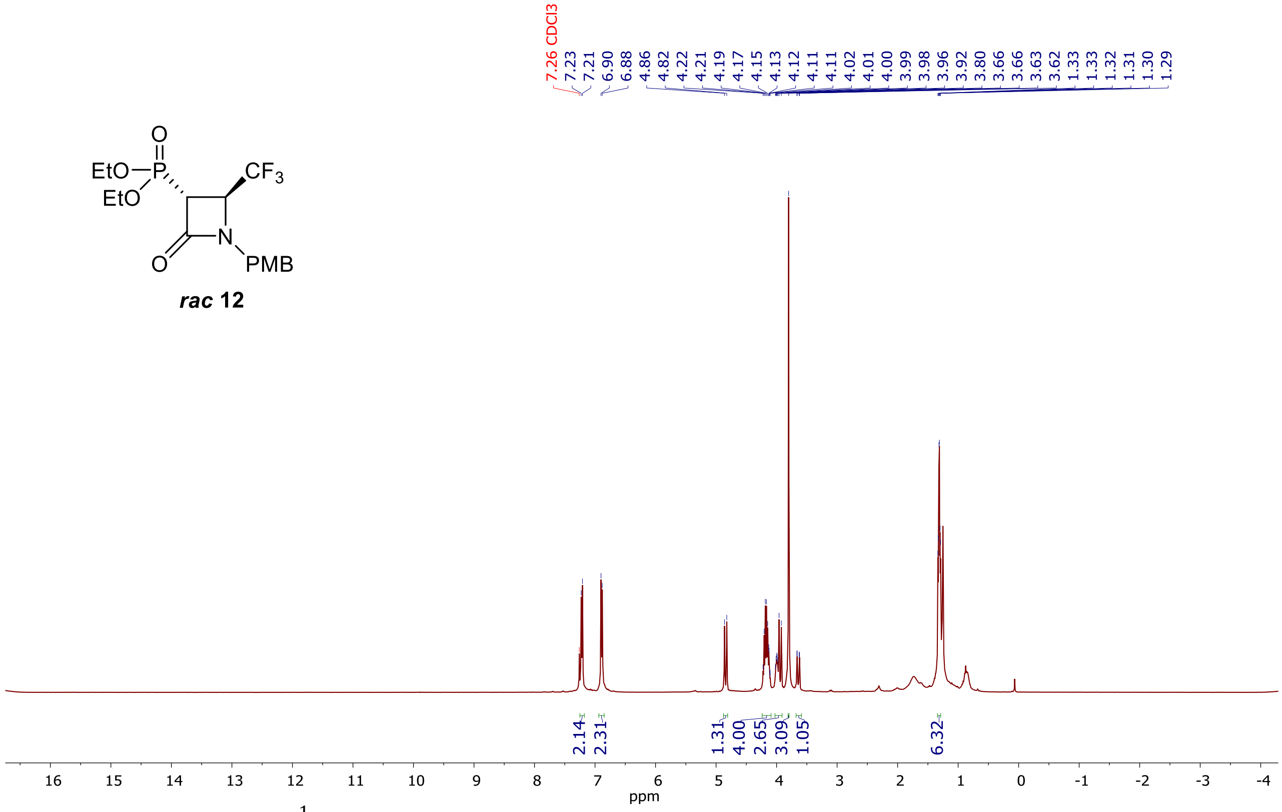

Figure S14. Spectrum ^{1}H NMR ($CDCl_3$, 400 MHz) of *rac* **12**

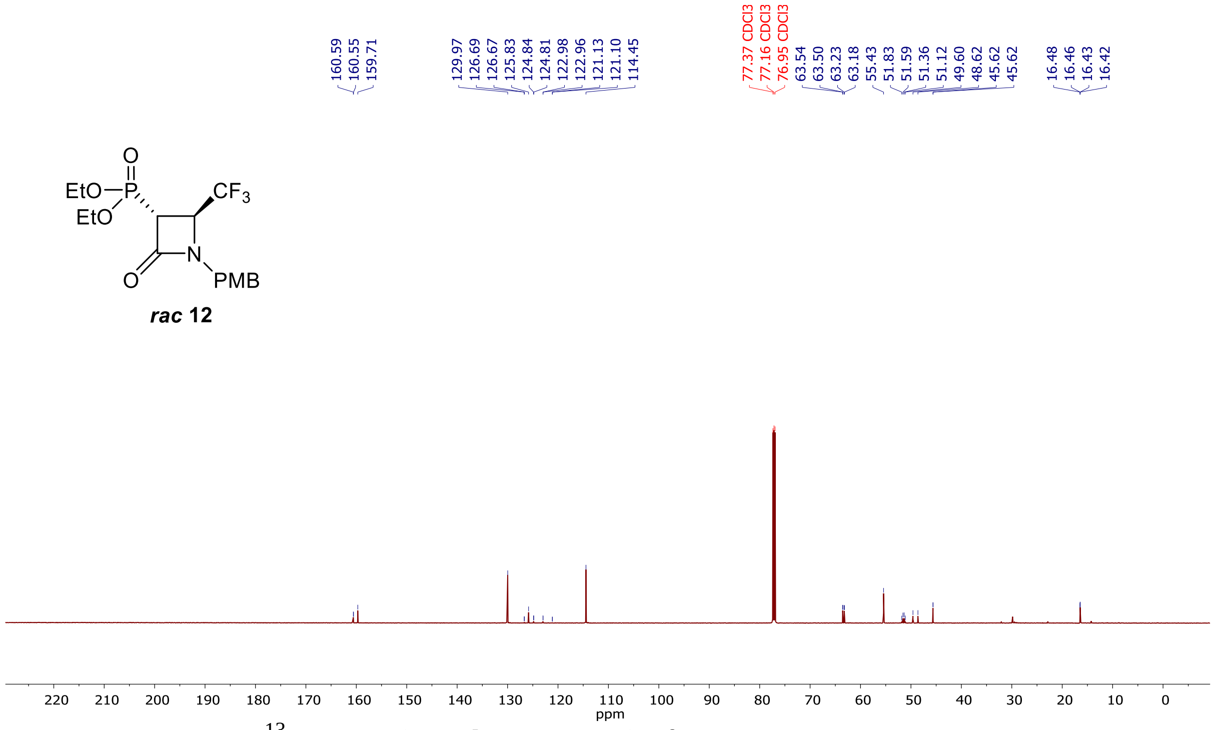

Figure S15. Spectrum ^{13}C NMR ($CDCl_3$, 151 MHz) of *rac* **12**

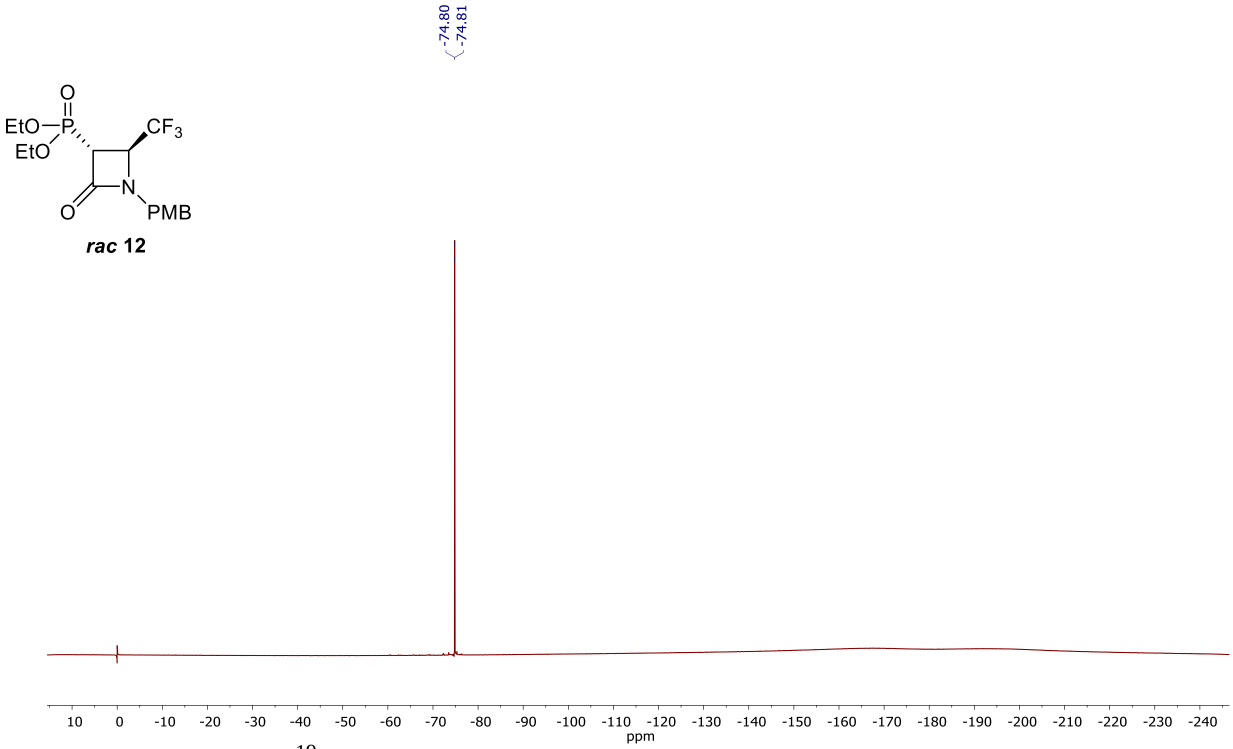

Figure S16. Spectrum ^{19}F NMR ($CDCl_3$, 376 MHz) of *rac* **12**

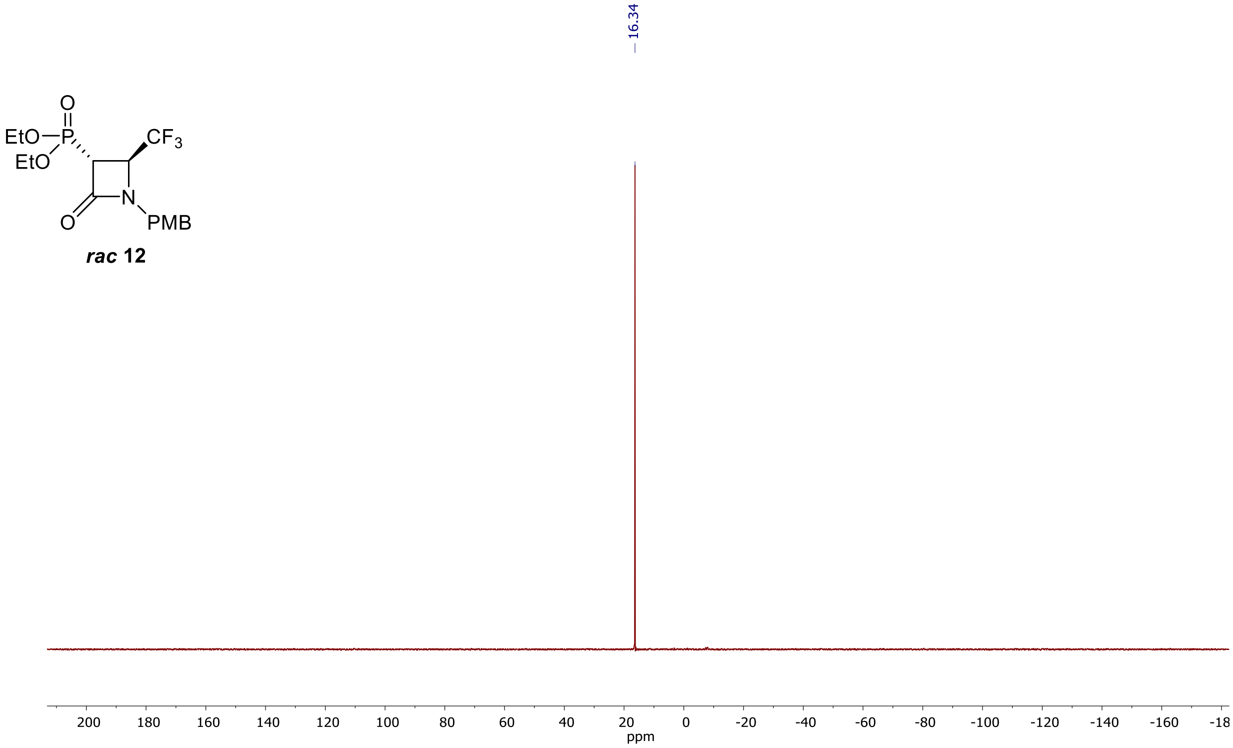

Figure S17. Spectrum ^{31}P NMR ($CDCl_3$, 243 MHz) of *rac* **12**

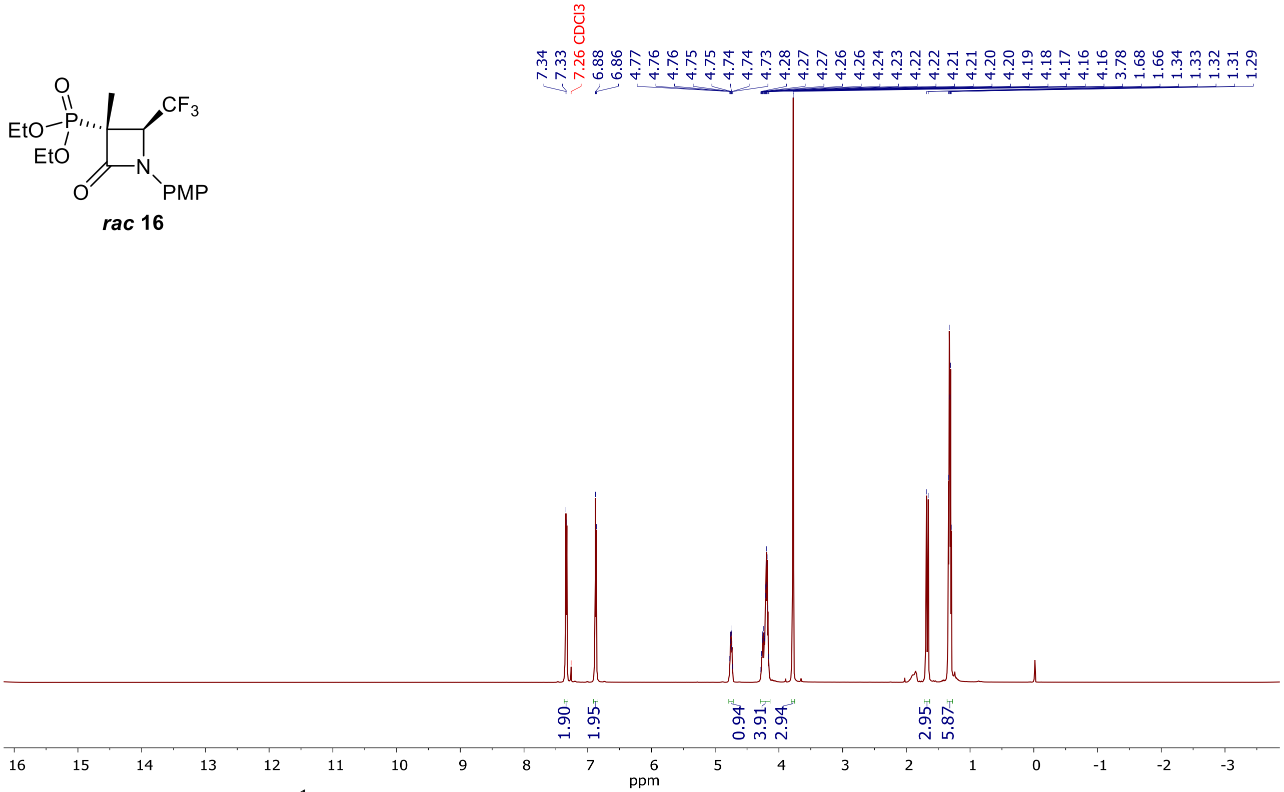

Figure S18. Spectrum ^{1}H NMR ($CDCl_3$, 600 MHz) of *rac* **16**

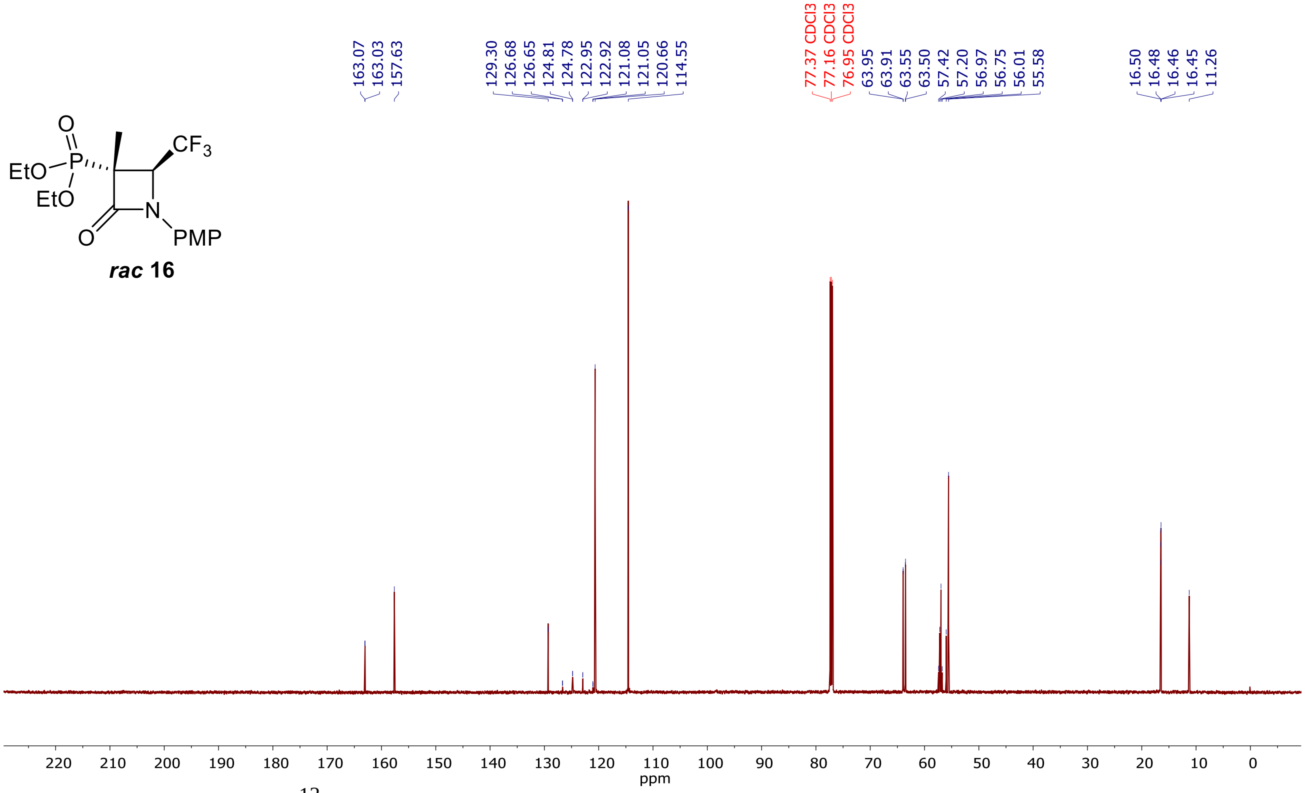

Figure S19. Spectrum ^{13}C NMR ($CDCl_3$, 151 MHz) of *rac* **16**

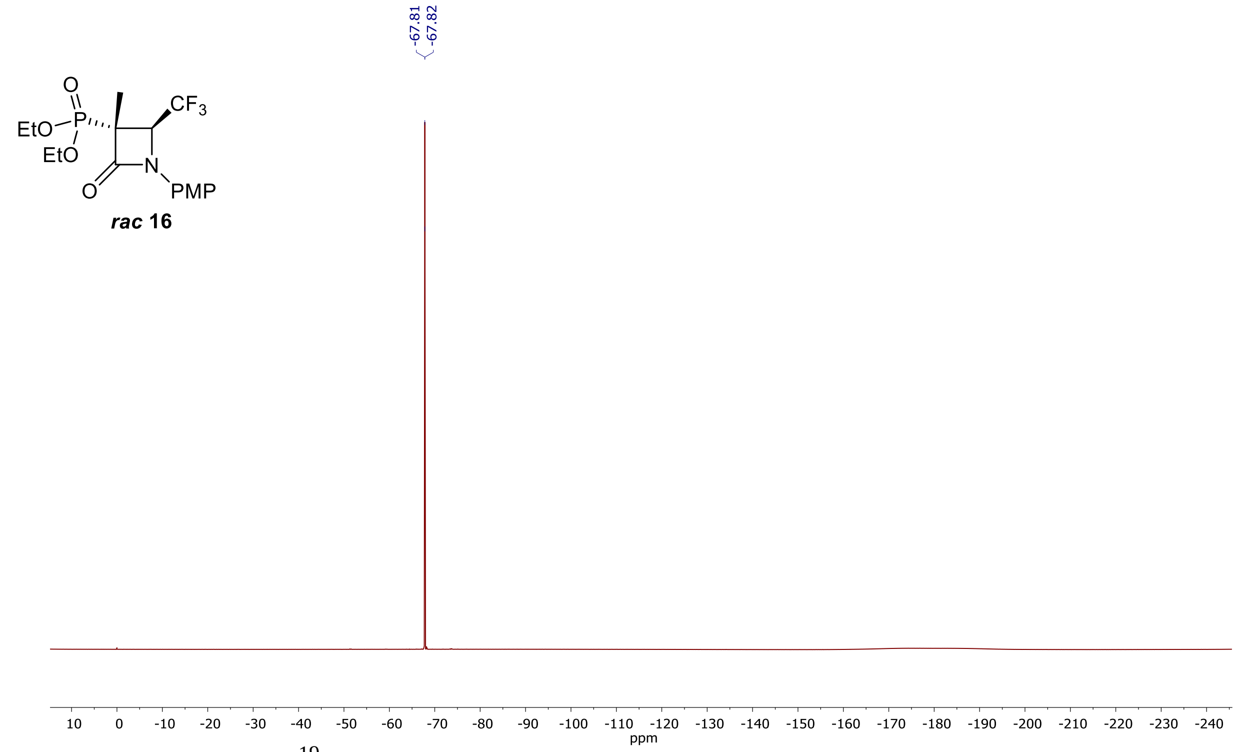

Figure S20. Spectrum ^{19}F NMR ($CDCl_3$, 565 MHz) of *rac* **16**

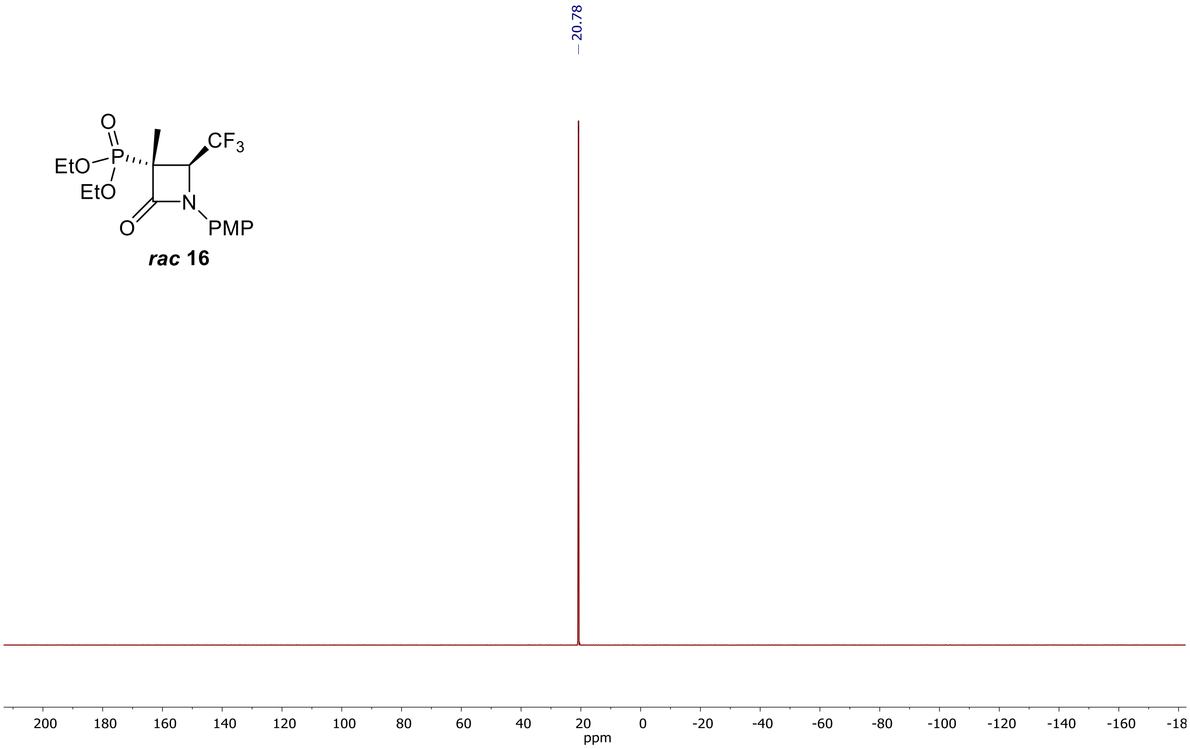

Figure S21. Spectrum ^{31}P NMR ($CDCl_3$, 243 MHz) of *rac* **16**

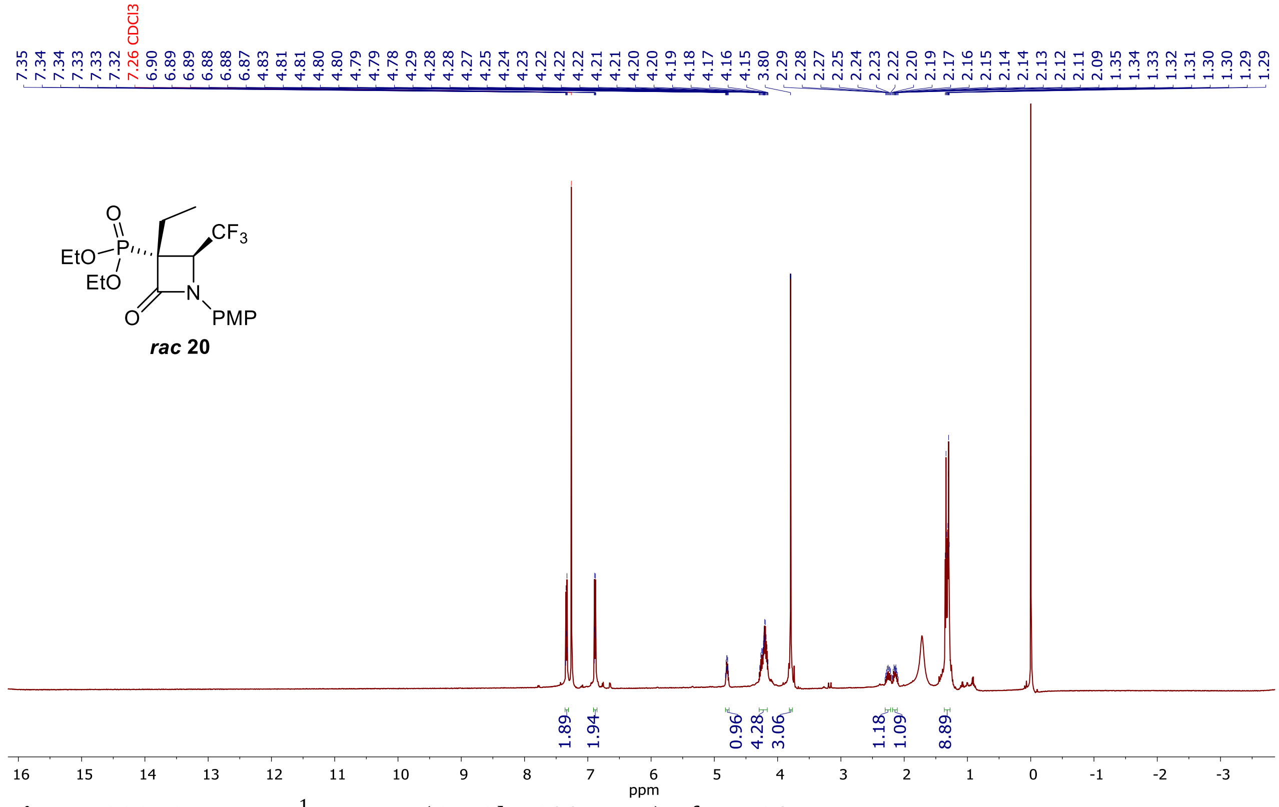

Figure S22. Spectrum ^{1}H NMR ($CDCl_3$, 600 MHz) of *rac* **20**

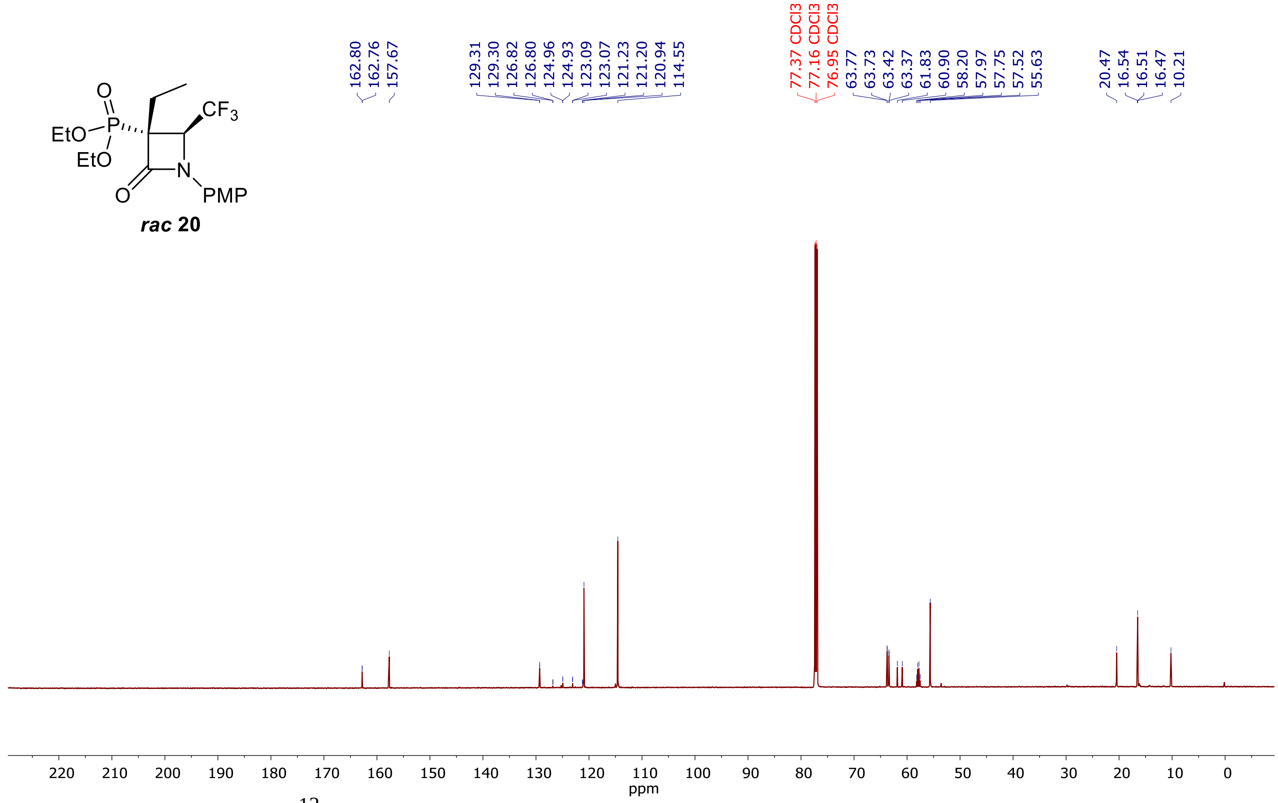

Figure S23. Spectrum ^{13}C NMR ($CDCl_3$, 151 MHz) of *rac* **20**

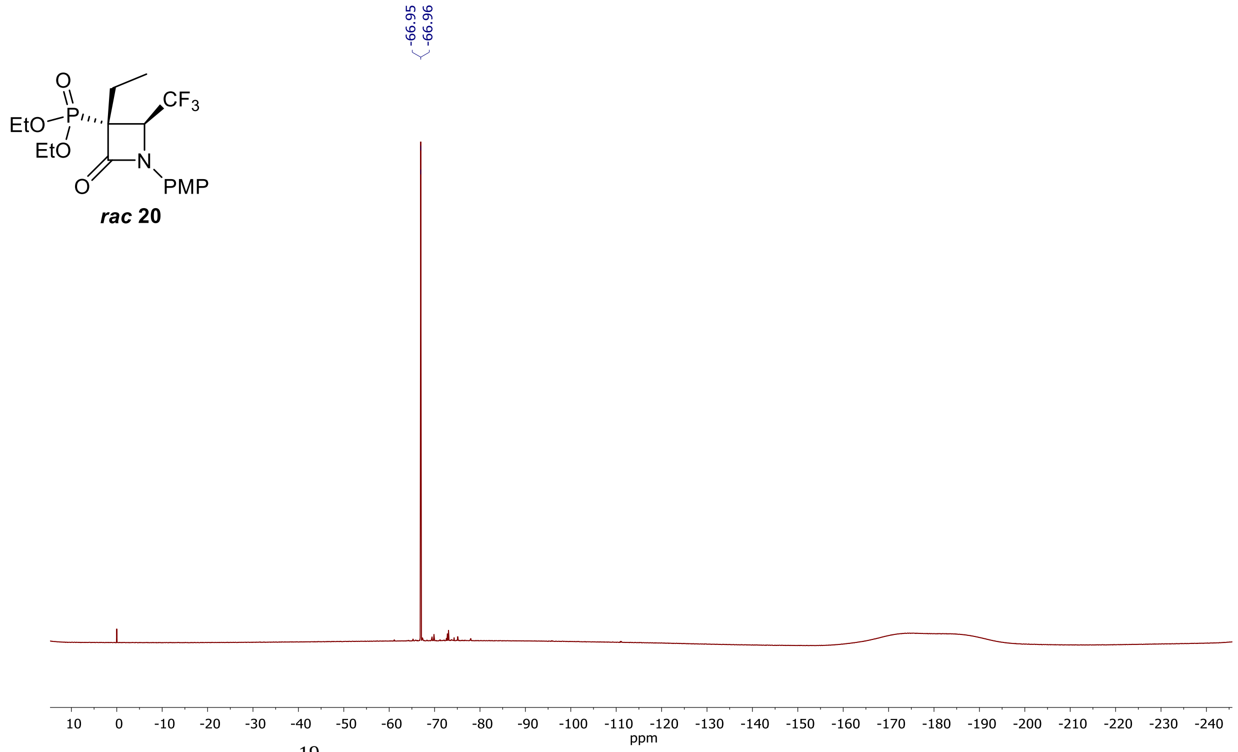

Figure S24. Spectrum ^{19}F NMR ($CDCl_3$, 565 MHz) of *rac* **20**

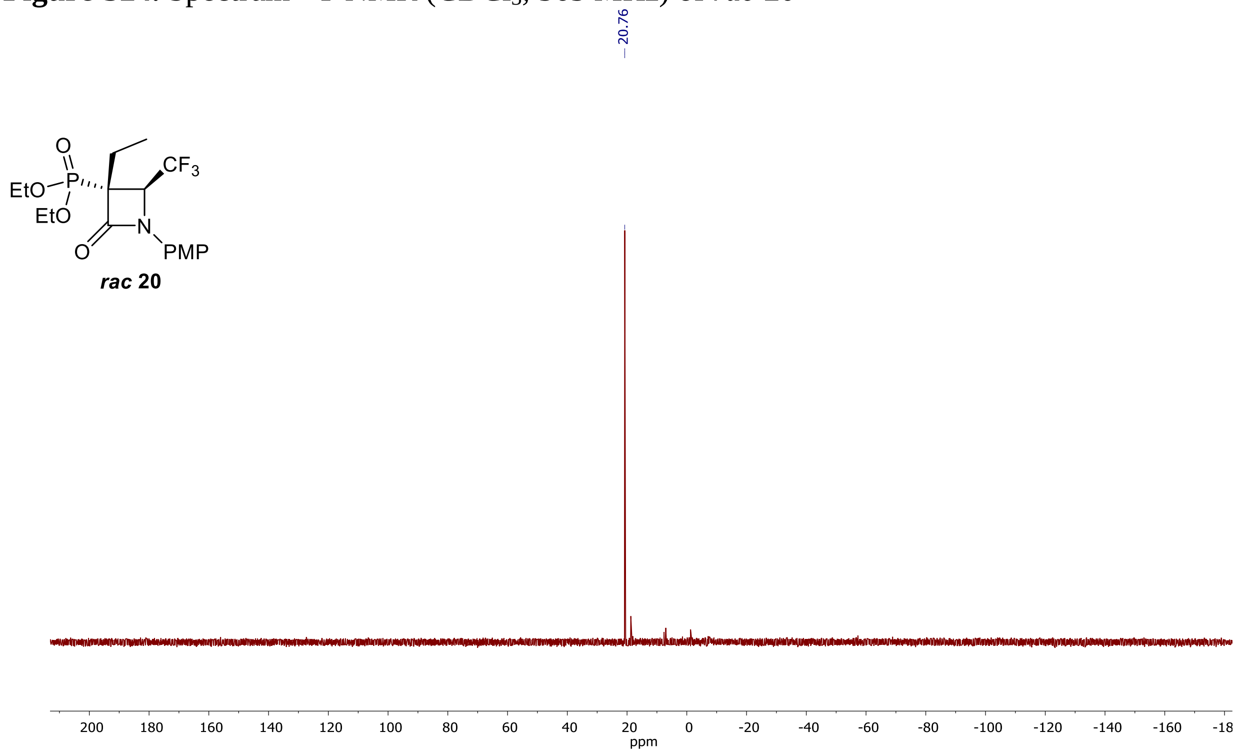

Figure S25. Spectrum ^{31}P NMR ($CDCl_3$, 243 MHz) of *rac* **20**

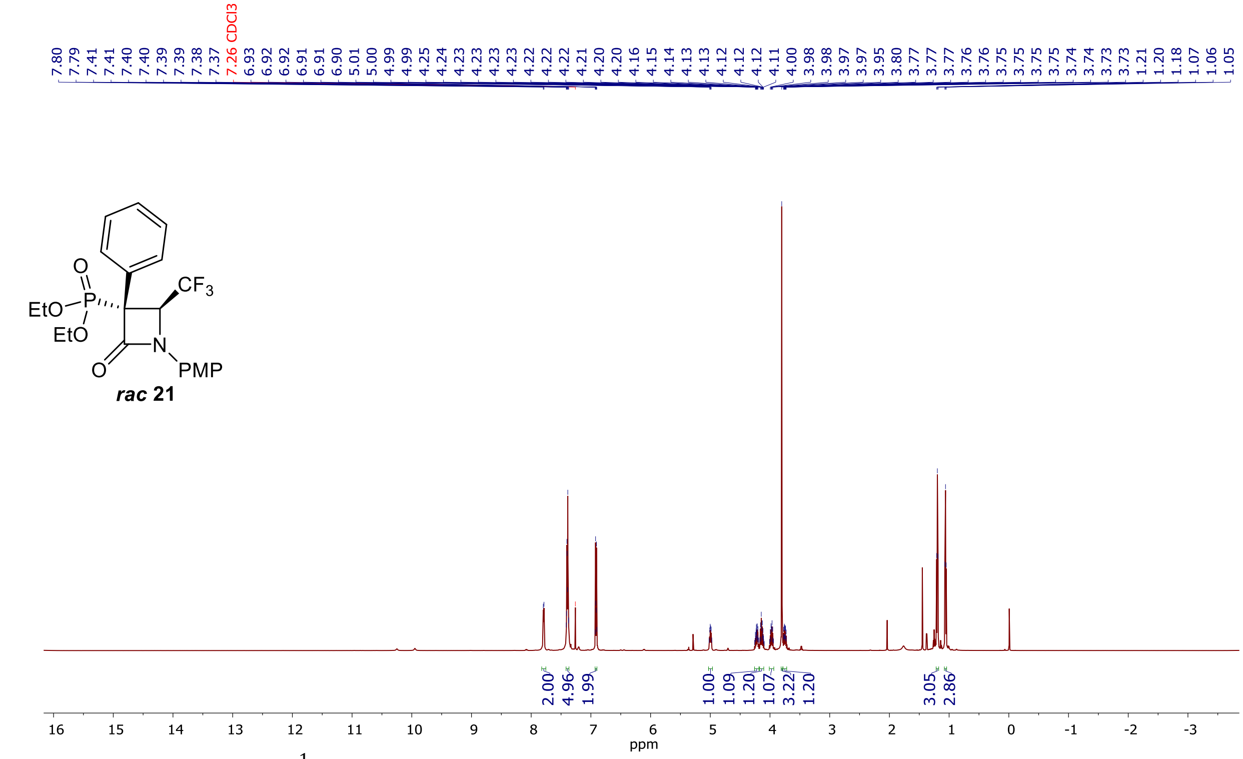

Figure S26. Spectrum ^{1}H NMR ($CDCl_3$, 600 MHz) of *rac* **21**

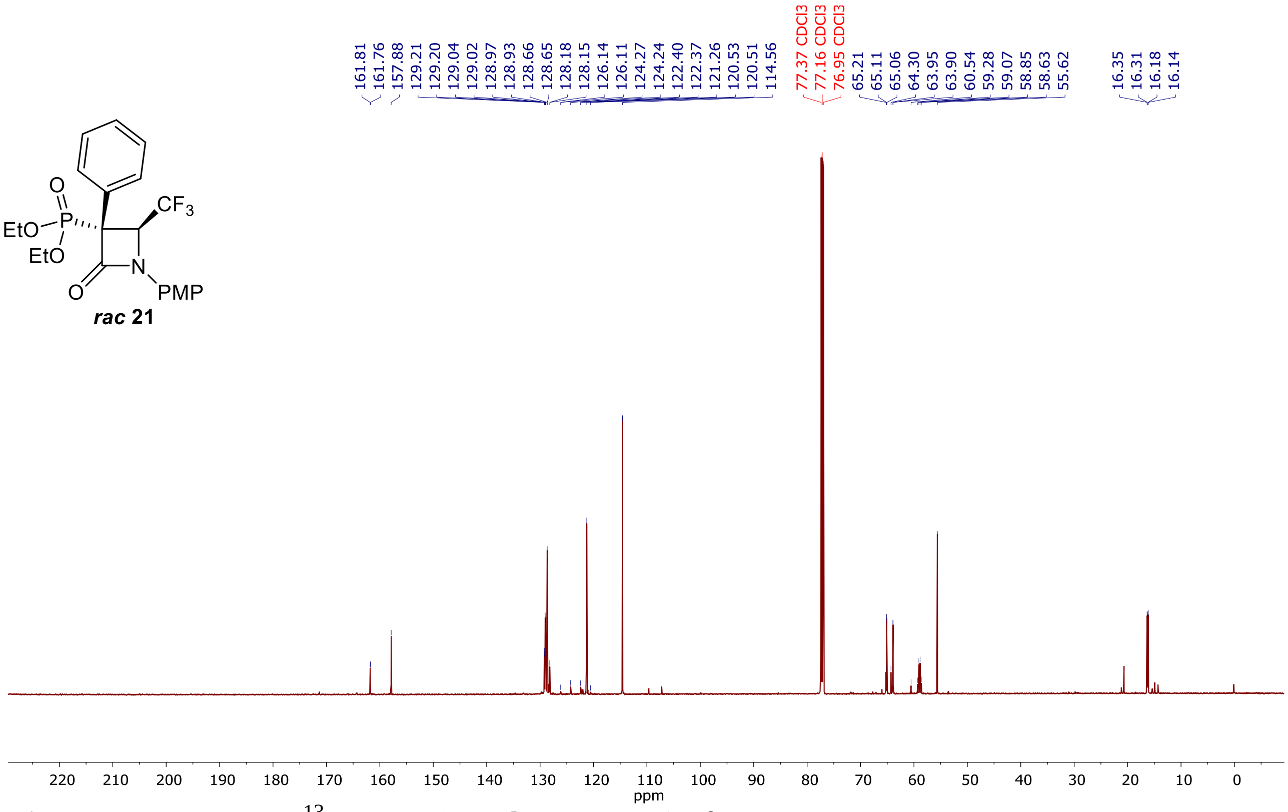

Figure S27. Spectrum ^{13}C NMR ($CDCl_3$, 151 MHz) of *rac* **21**

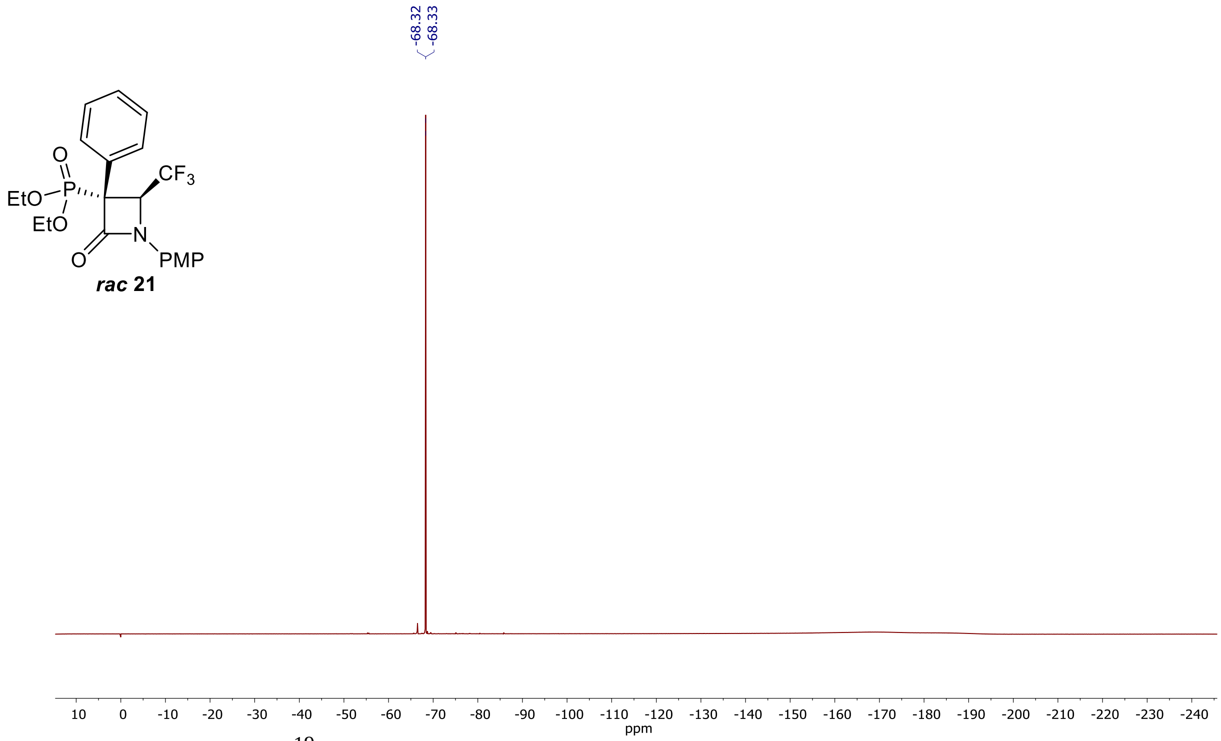

Figure S28. Spectrum ^{19}F NMR ($CDCl_3$, 565 MHz) of *rac* **21**

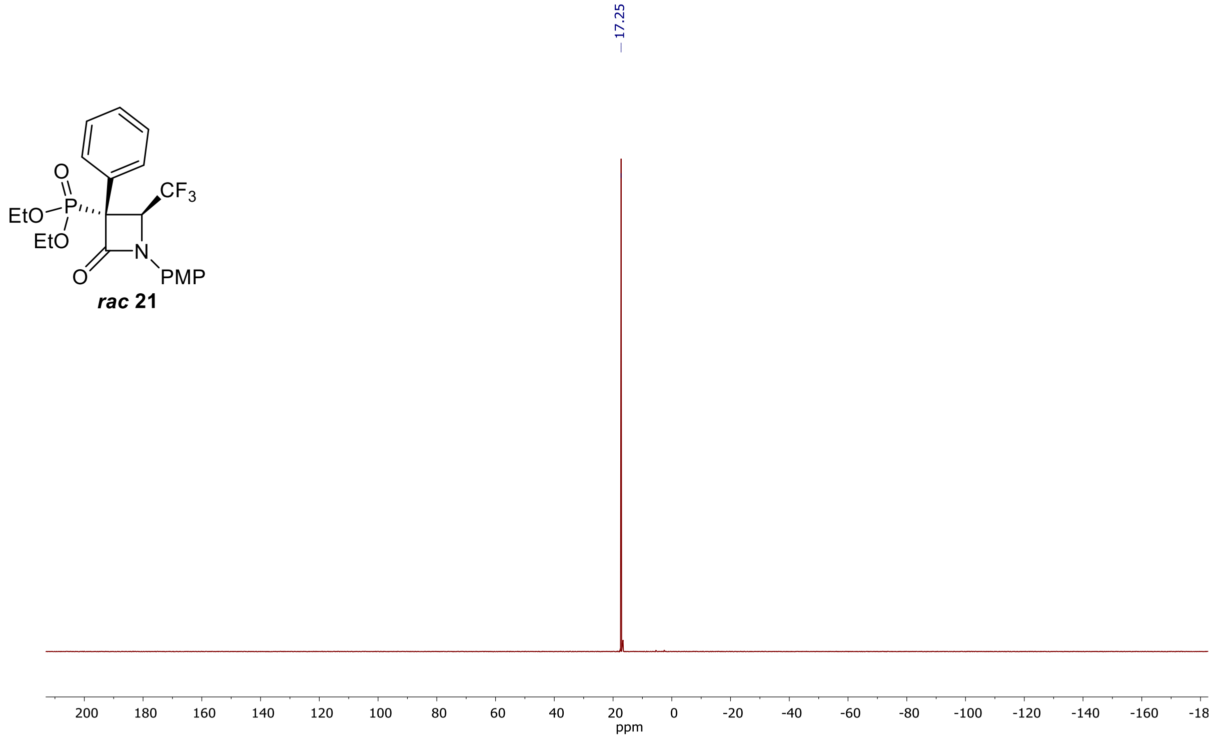

Figure S29. Spectrum ^{31}P NMR ($CDCl_3$, 243 MHz) of *rac* **21**

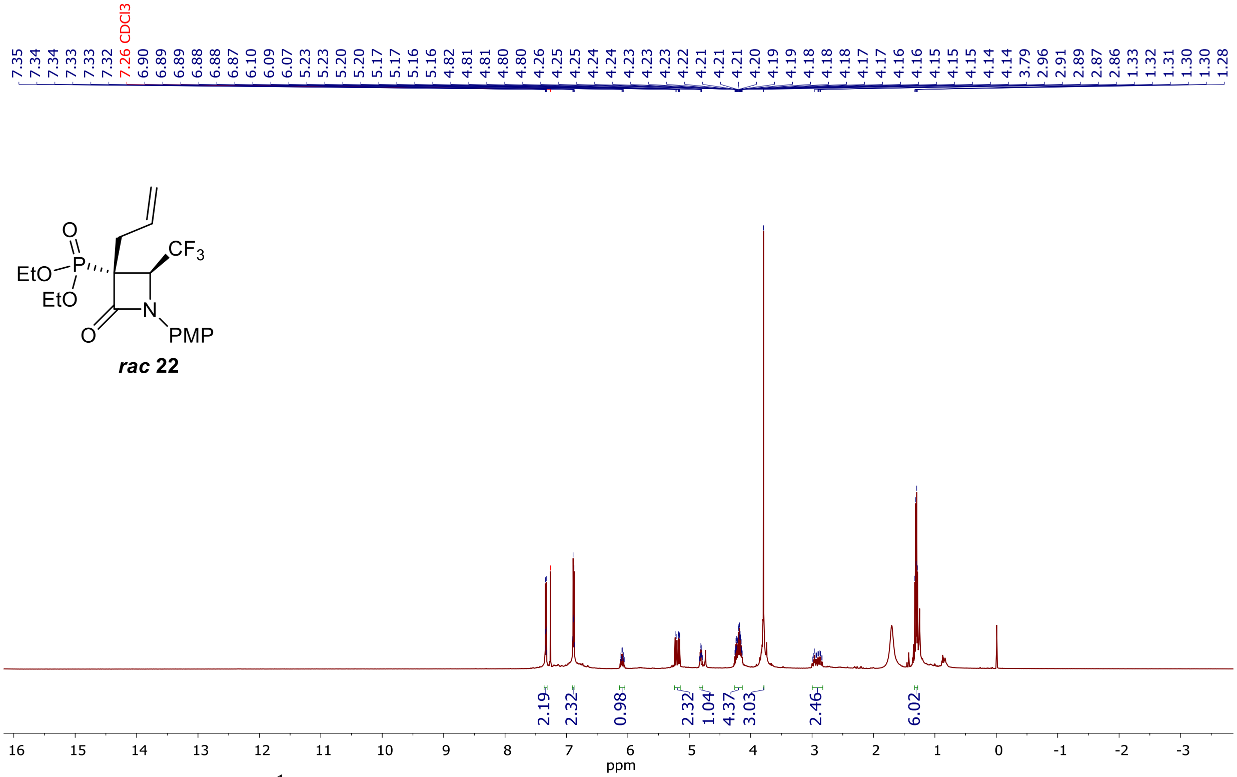

Figure S30. Spectrum ^{1}H NMR ($CDCl_3$, 600 MHz) of *rac* **22**

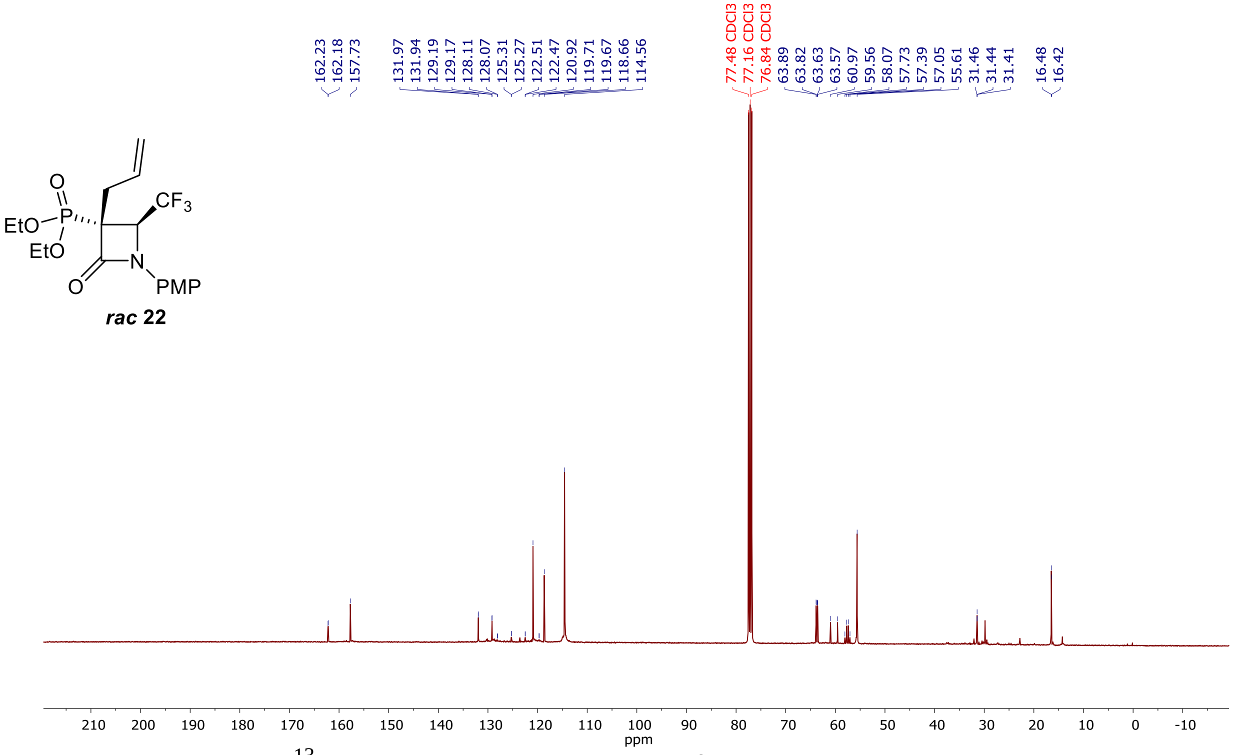

Figure S31. Spectrum ^{13}C NMR ($CDCl_3$, 101 MHz) of *rac* **22**

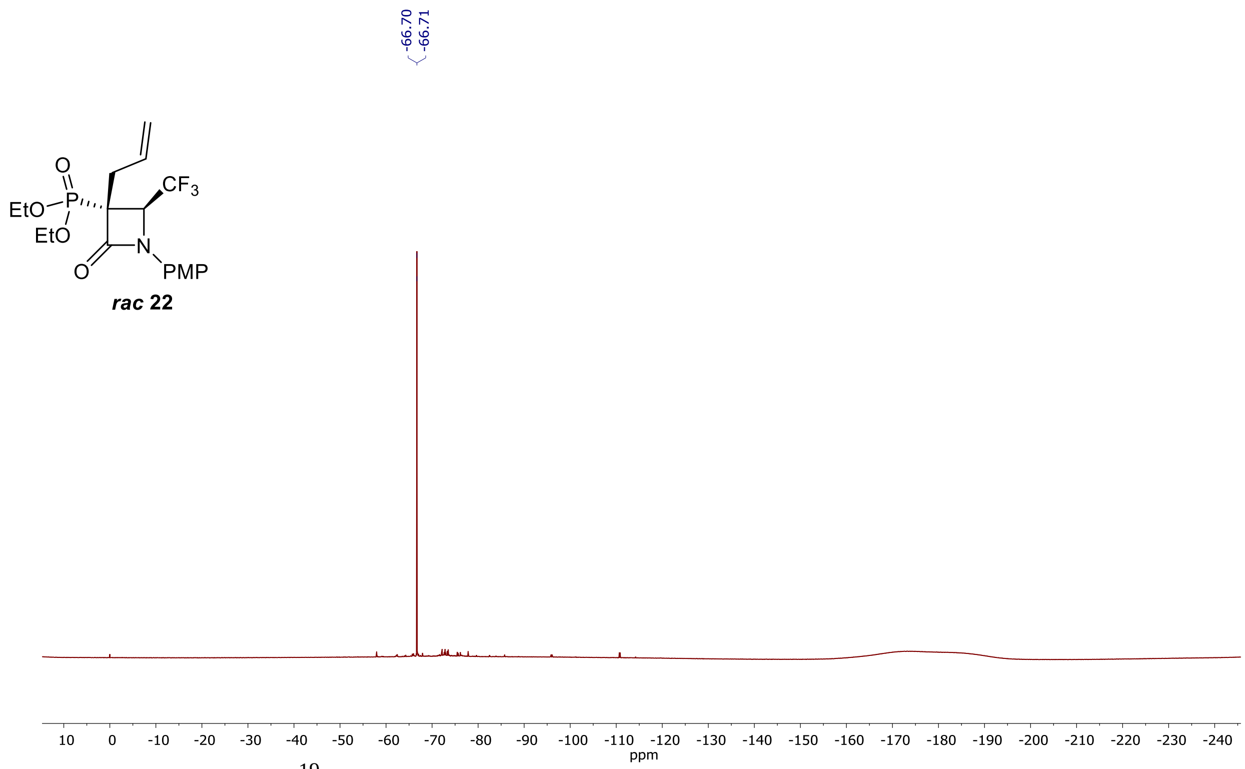

Figure S32. Spectrum ^{19}F NMR ($CDCl_3$, 565 MHz) of *rac* **22**

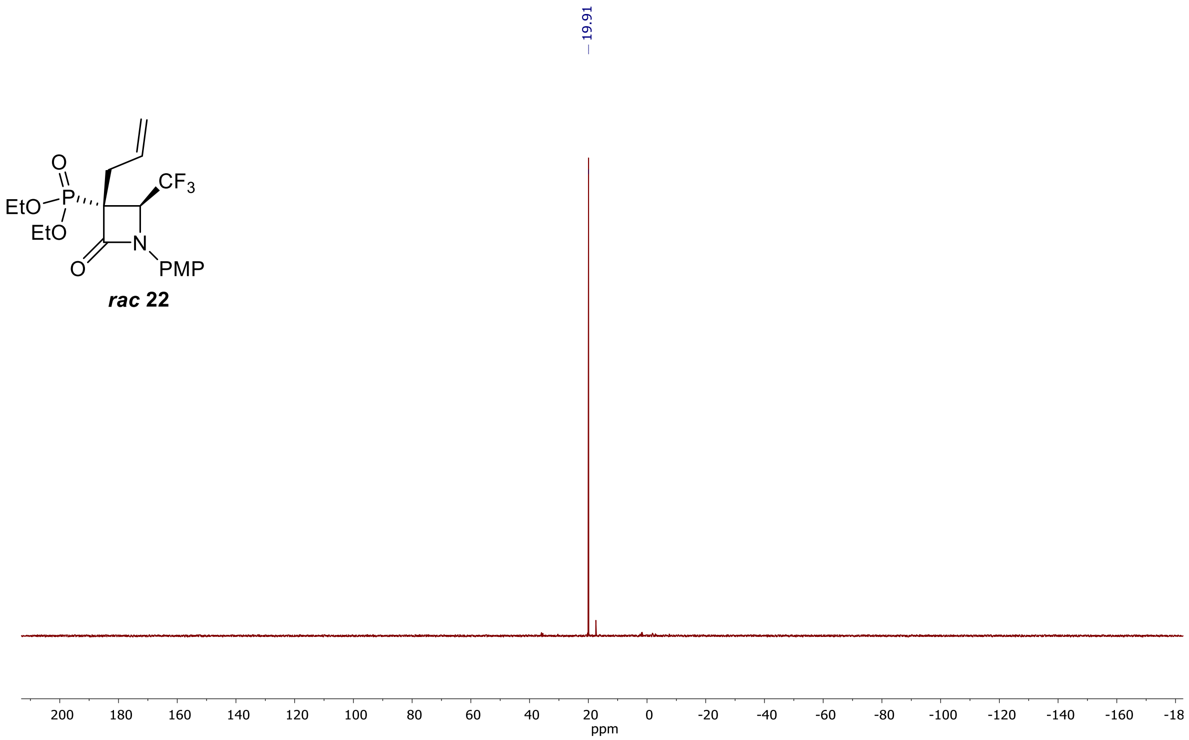

Figure S33. Spectrum ^{31}P NMR ($CDCl_3$, 243 MHz) of *rac* **22**

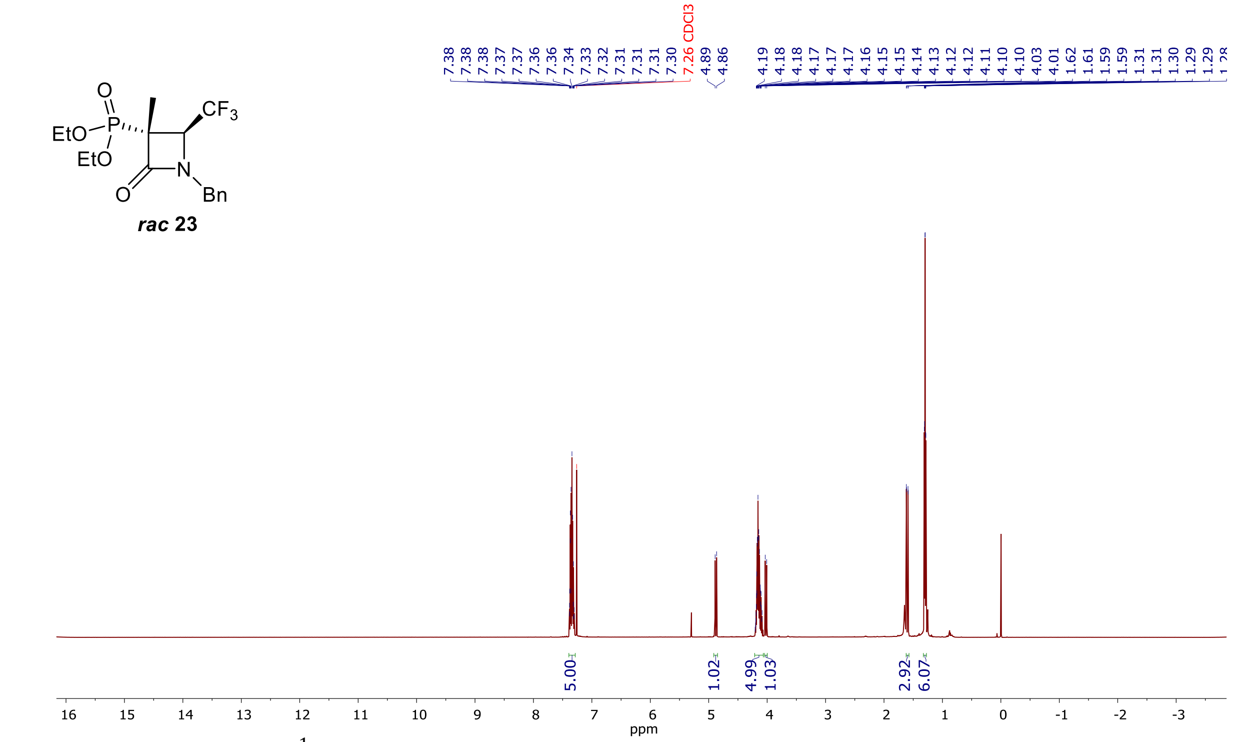

Figure S34. Spectrum ^{1}H NMR ($CDCl_3$, 600 MHz) of *rac* **23**

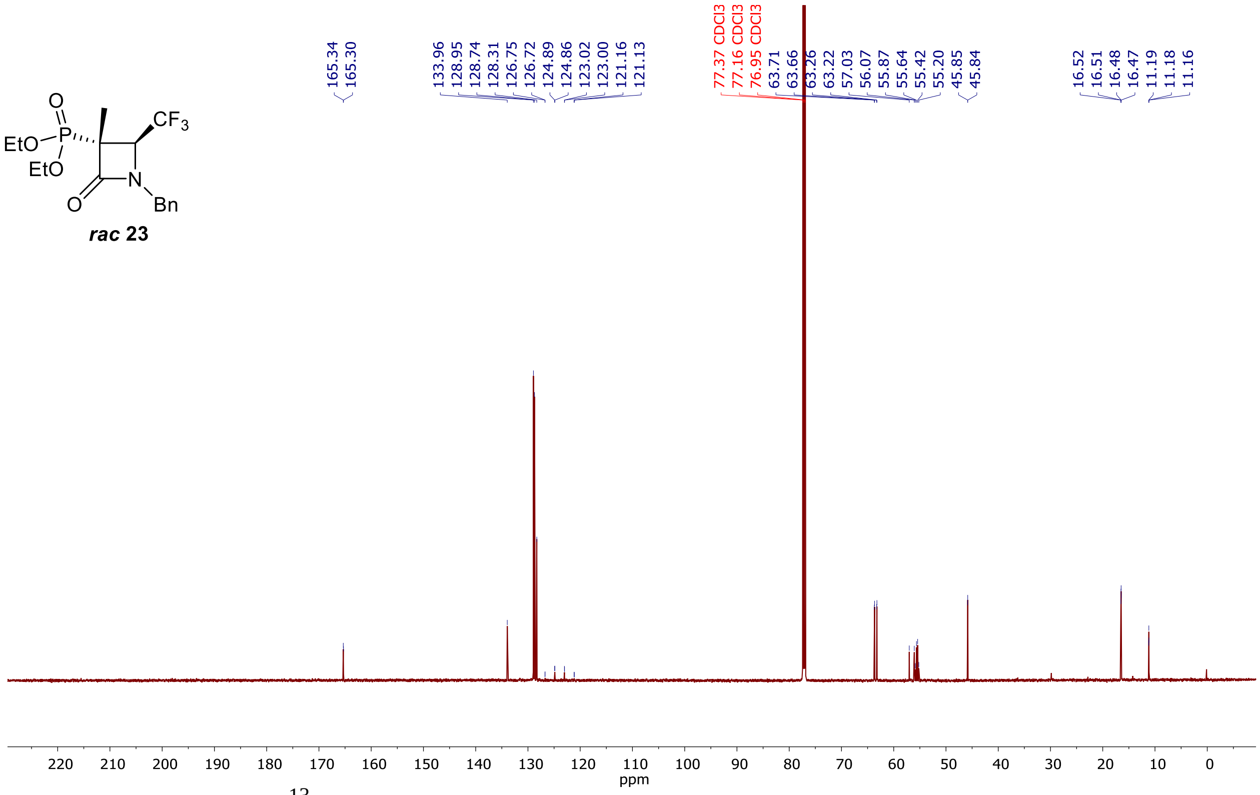

Figure S35. Spectrum ^{13}C NMR ($CDCl_3$, 151 MHz) of *rac* **23**

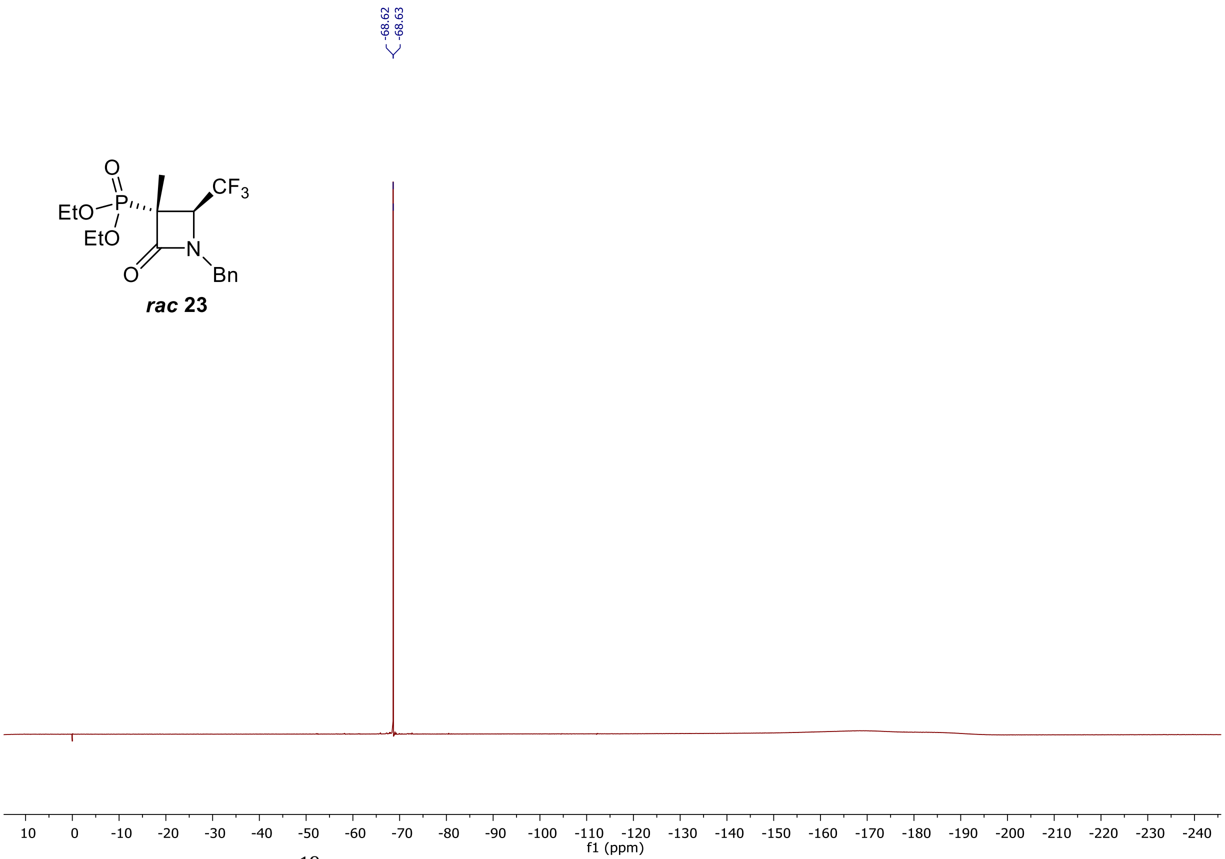

Figure S36. Spectrum ^{19}F NMR ($CDCl_3$, 565 MHz) of *rac* **23**

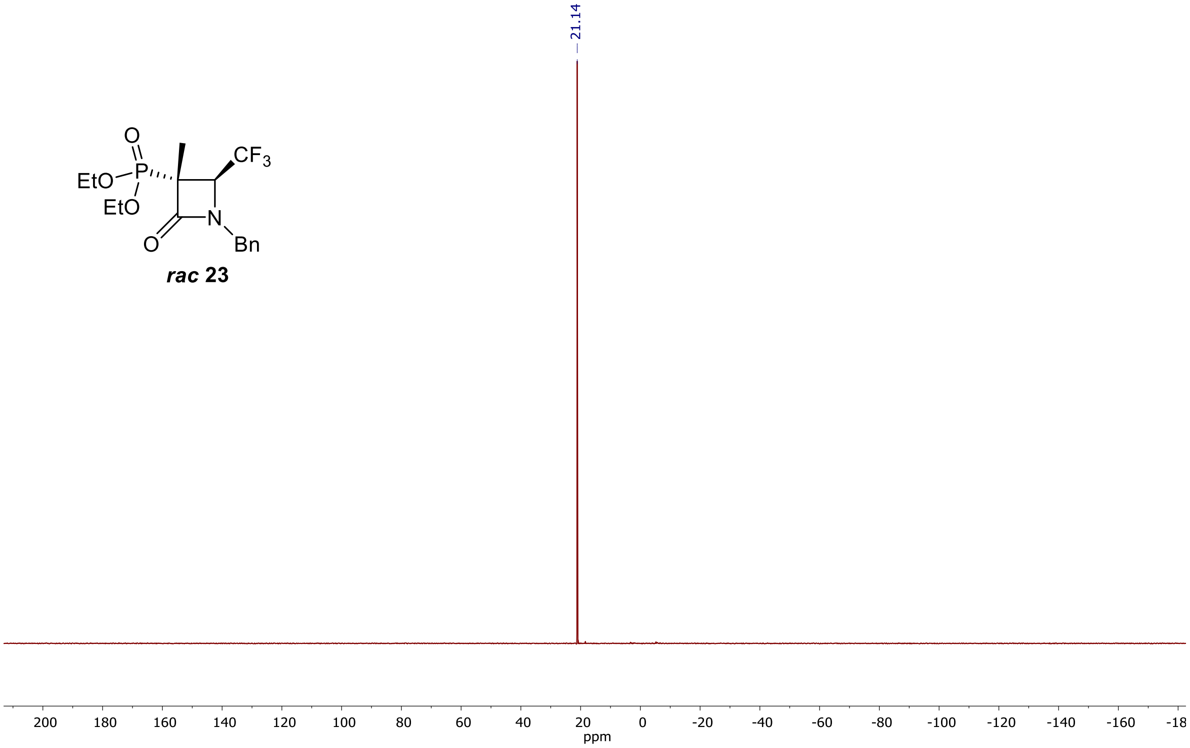

Figure S37. Spectrum ^{31}P NMR ($CDCl_3$, 243 MHz) of *rac* **23**

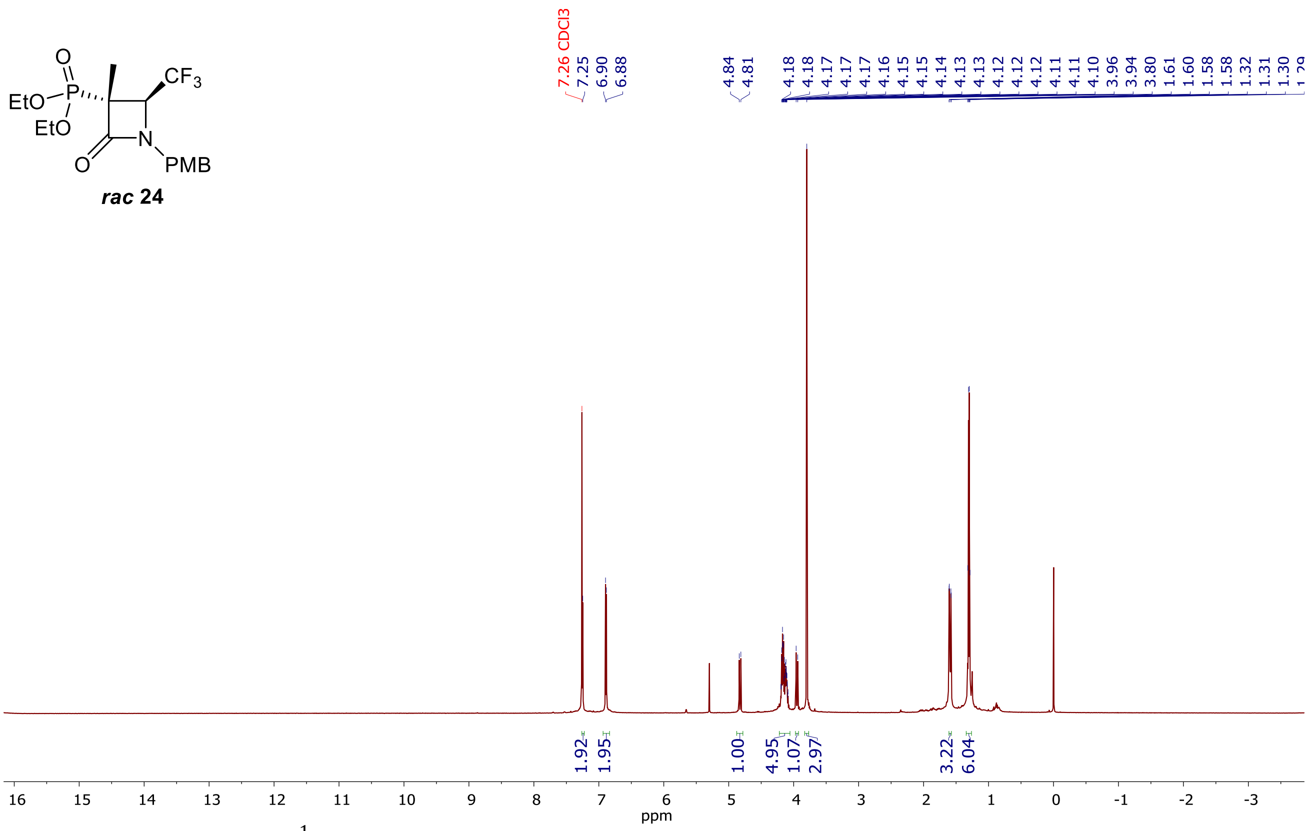

Figure S38. Spectrum ^{1}H NMR ($CDCl_3$, 600 MHz) of *rac* **24**

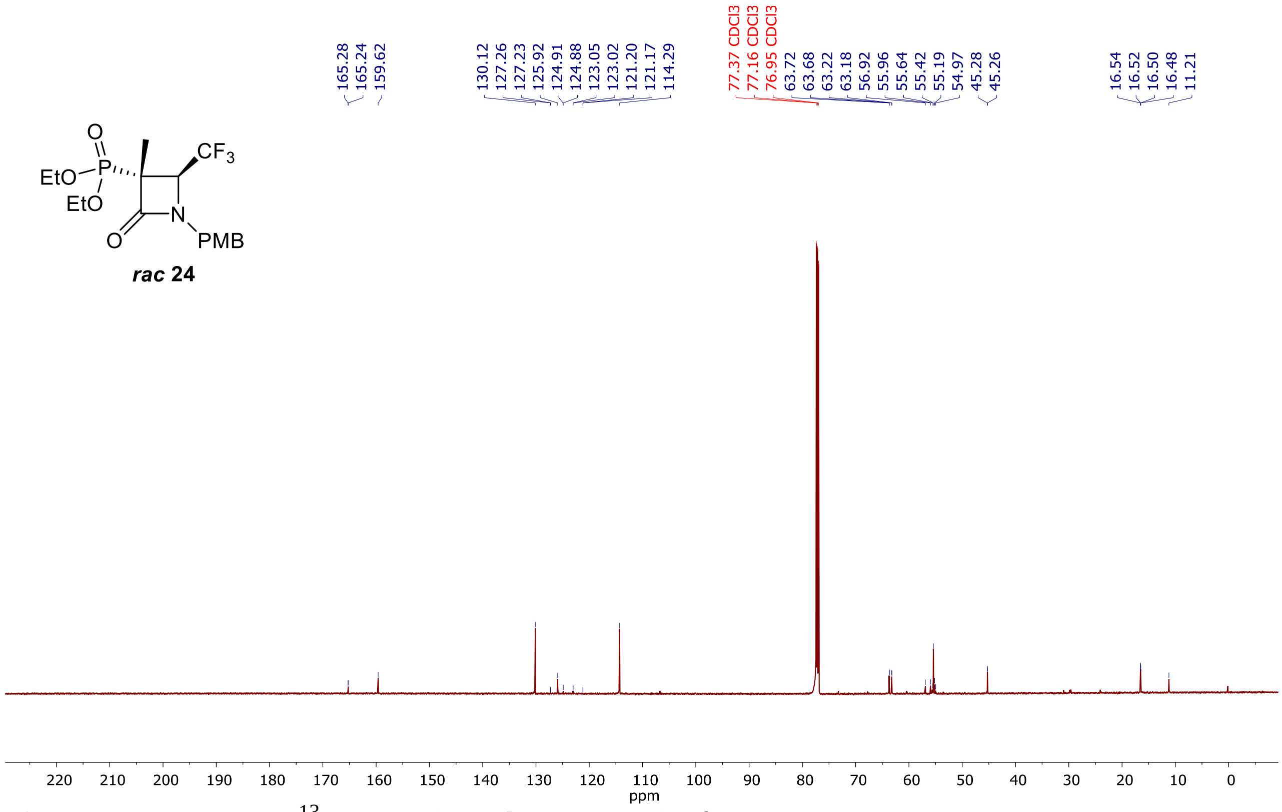

Figure S39. Spectrum ^{13}C NMR ($CDCl_3$, 151 MHz) of *rac* **24**

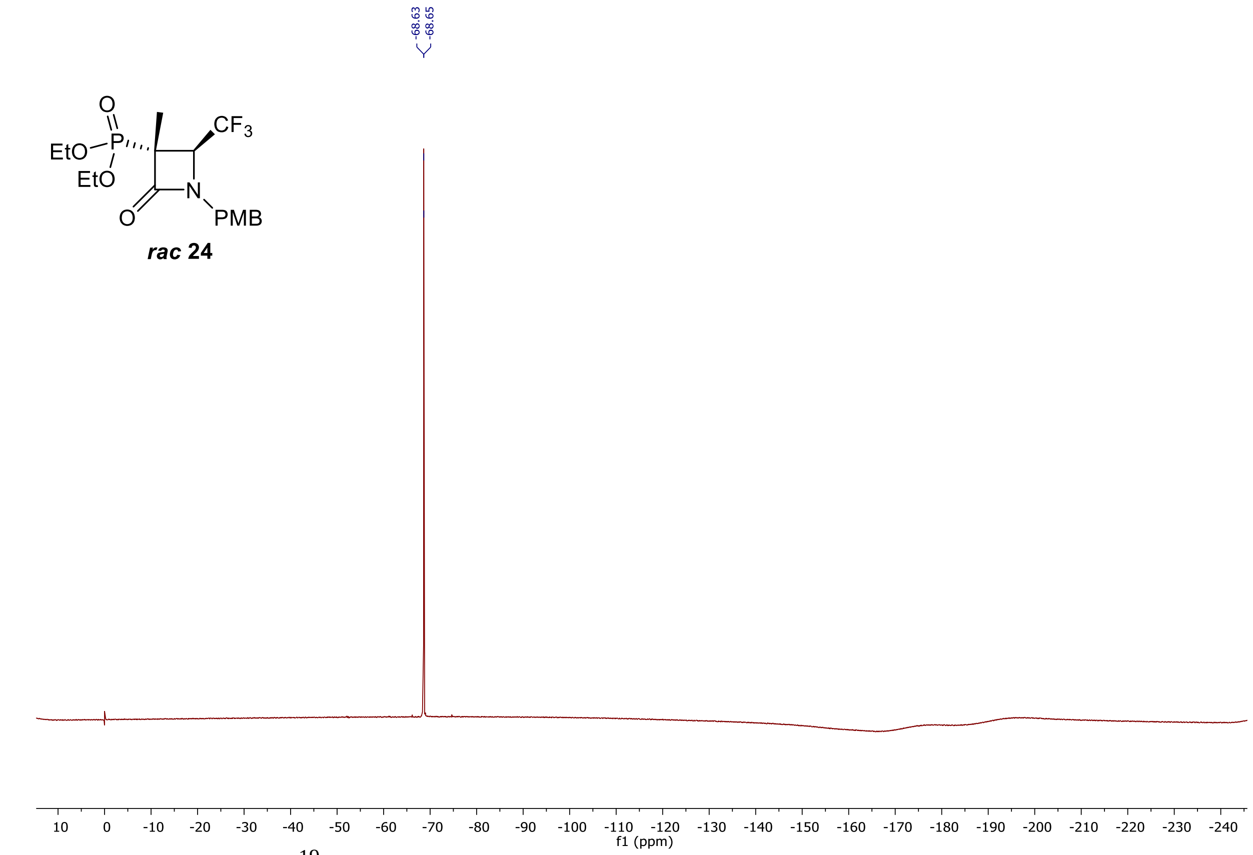

Figure S40. Spectrum ^{19}F NMR ($CDCl_3$, 565 MHz) of *rac* **24**

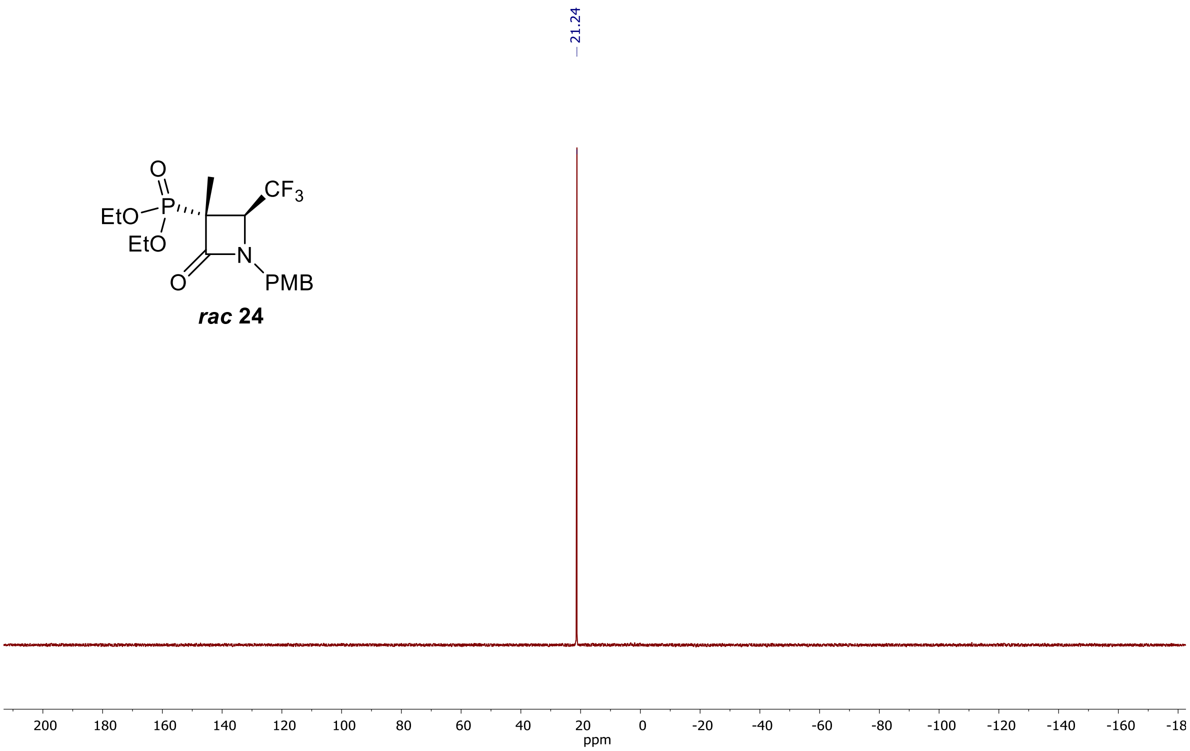

Figure S41. Spectrum ^{31}P NMR ($CDCl_3$, 243 MHz) of *rac* **24**

5. ^{1}H, ^{13}C, ^{19}F Spectra of new 4-CF_3-*β*-lactams

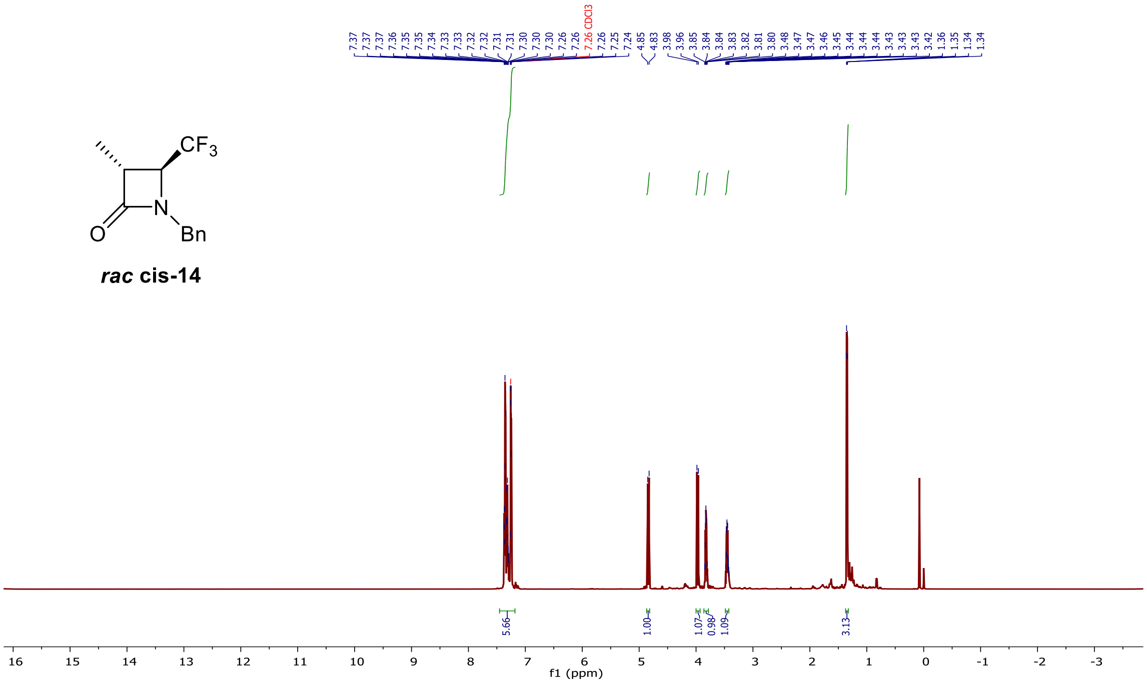

Figure S42. Spectrum ^{1}H NMR ($CDCl_3$, 600 MHz) of *rac* cis-**14**

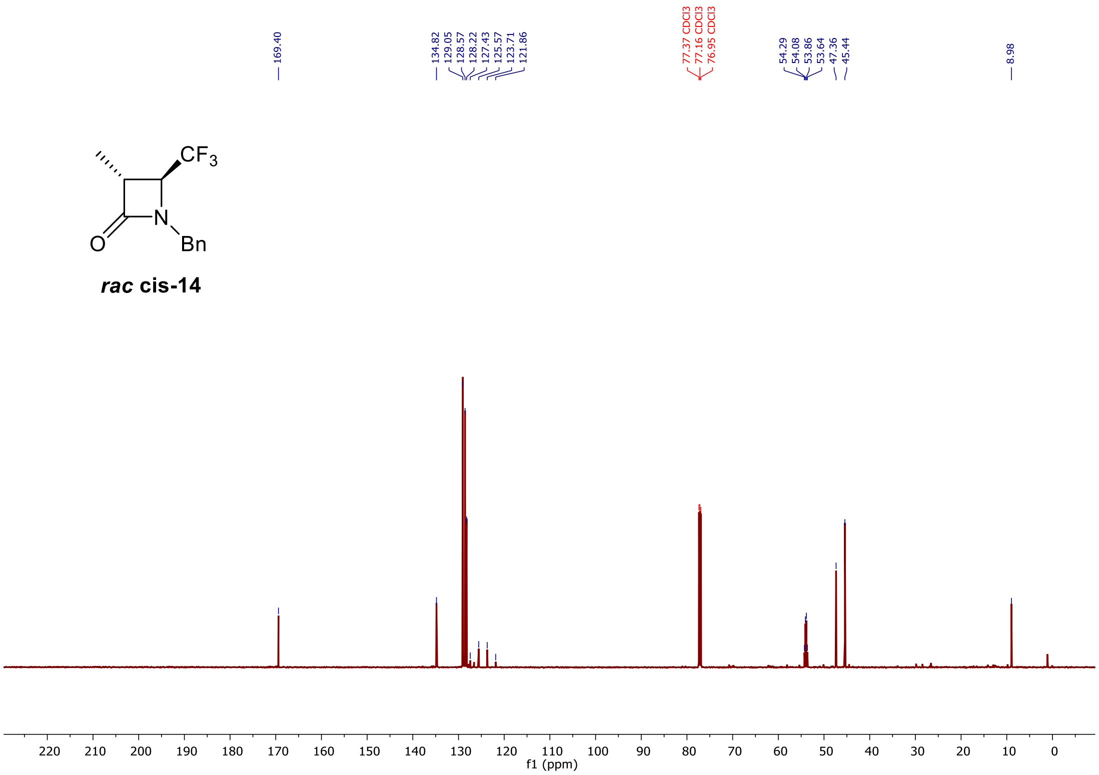

Figure S43. Spectrum ^{13}C NMR ($CDCl_3$, 151 MHz) of *rac* cis-**14**

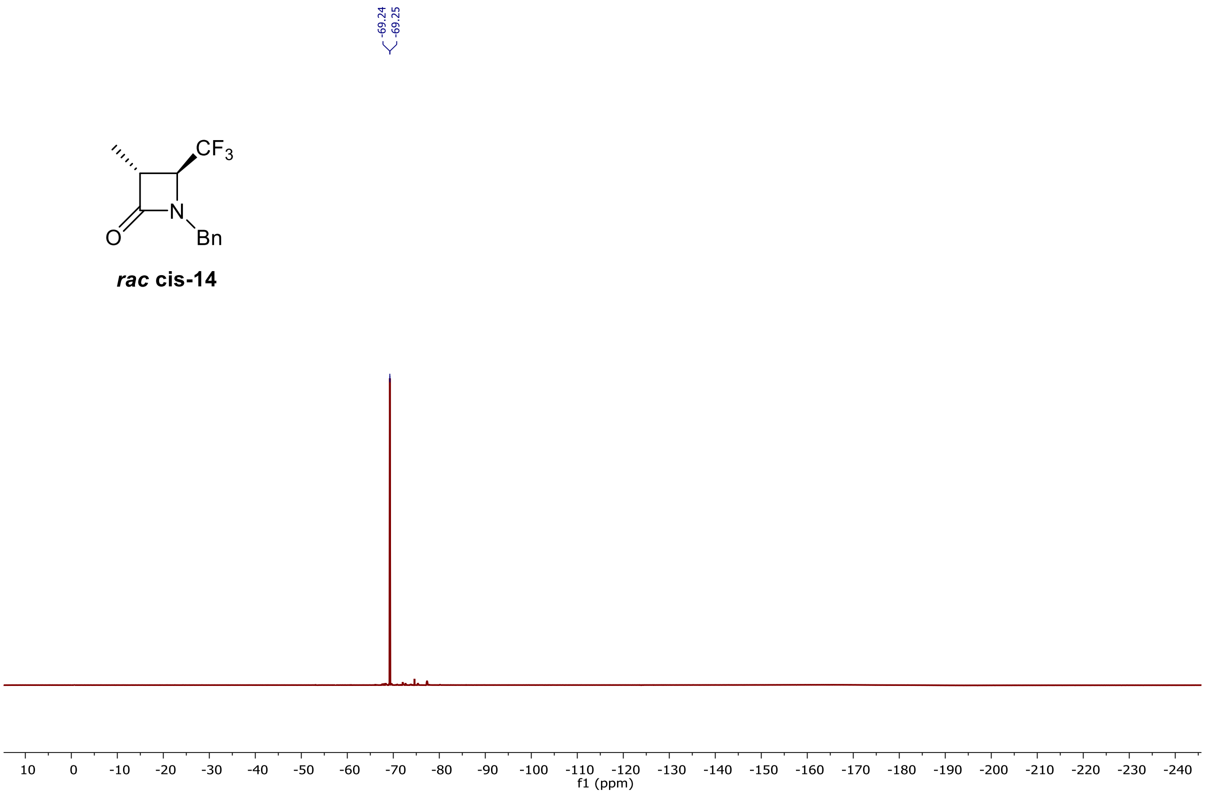

Figure S44. Spectrum ^{19}F NMR ($CDCl_3$, 565 MHz) of *rac* cis-**14**

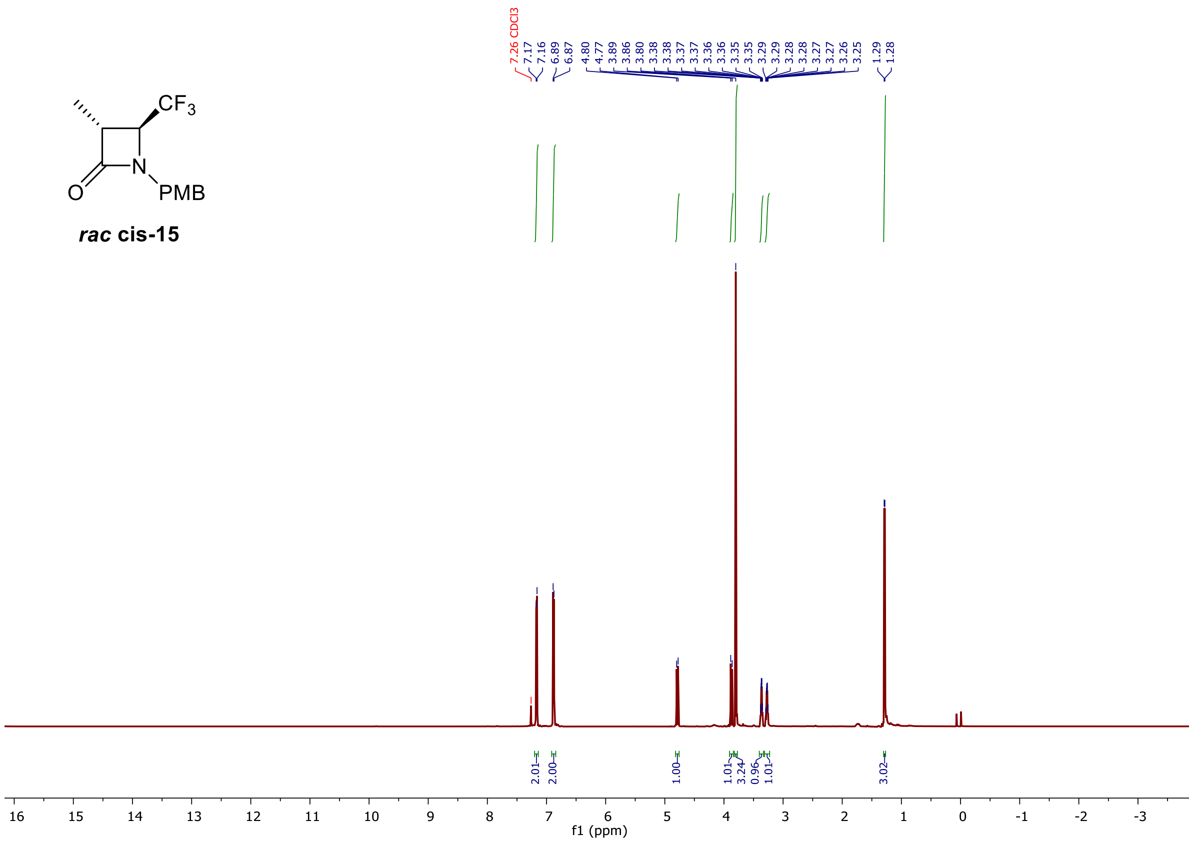

Figure S45. Spectrum ^{1}H NMR ($CDCl_3$, 600 MHz) of *rac* cis-**15**

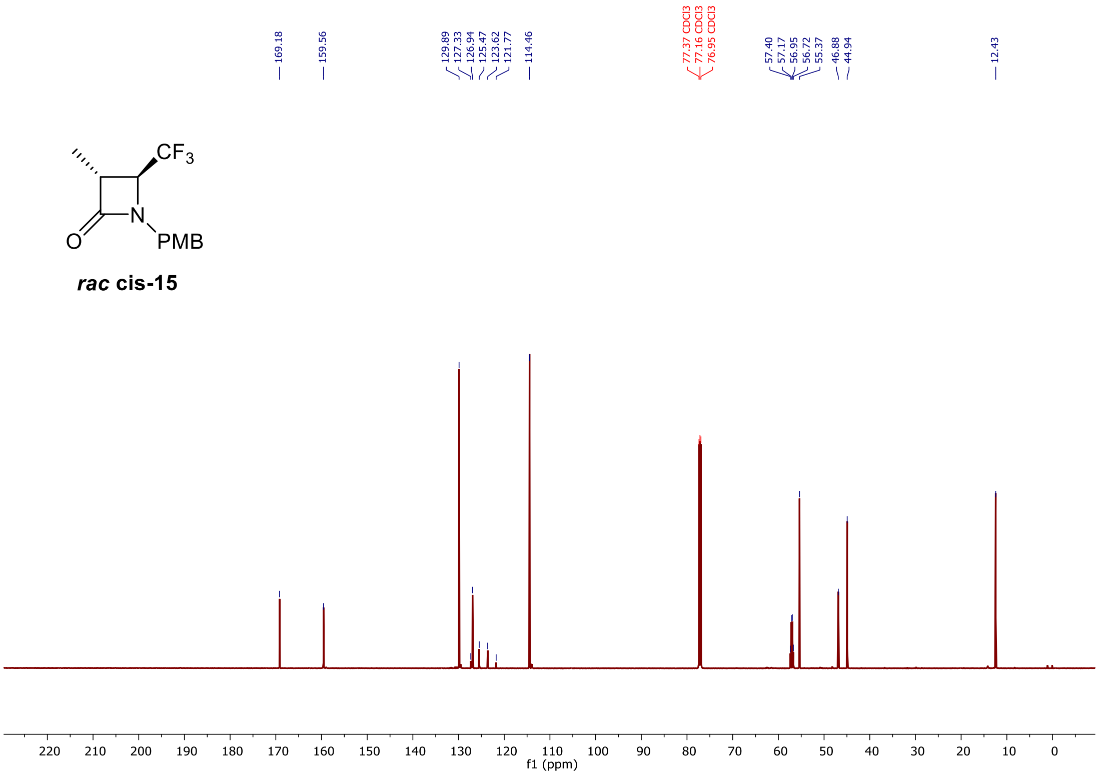

Figure S46. Spectrum ^{13}C NMR ($CDCl_3$, 151 MHz) of *rac* cis-**15**

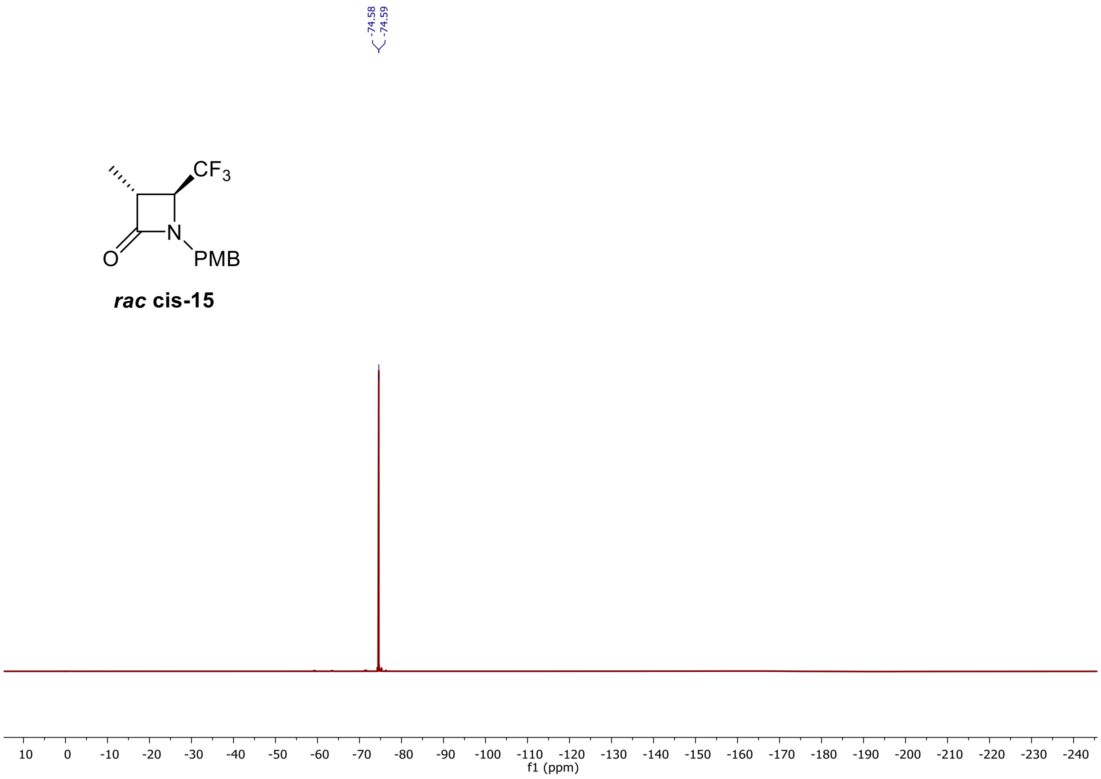

Figure S47. Spectrum ^{19}F NMR ($CDCl_3$, 565 MHz) of *rac* cis-**15**

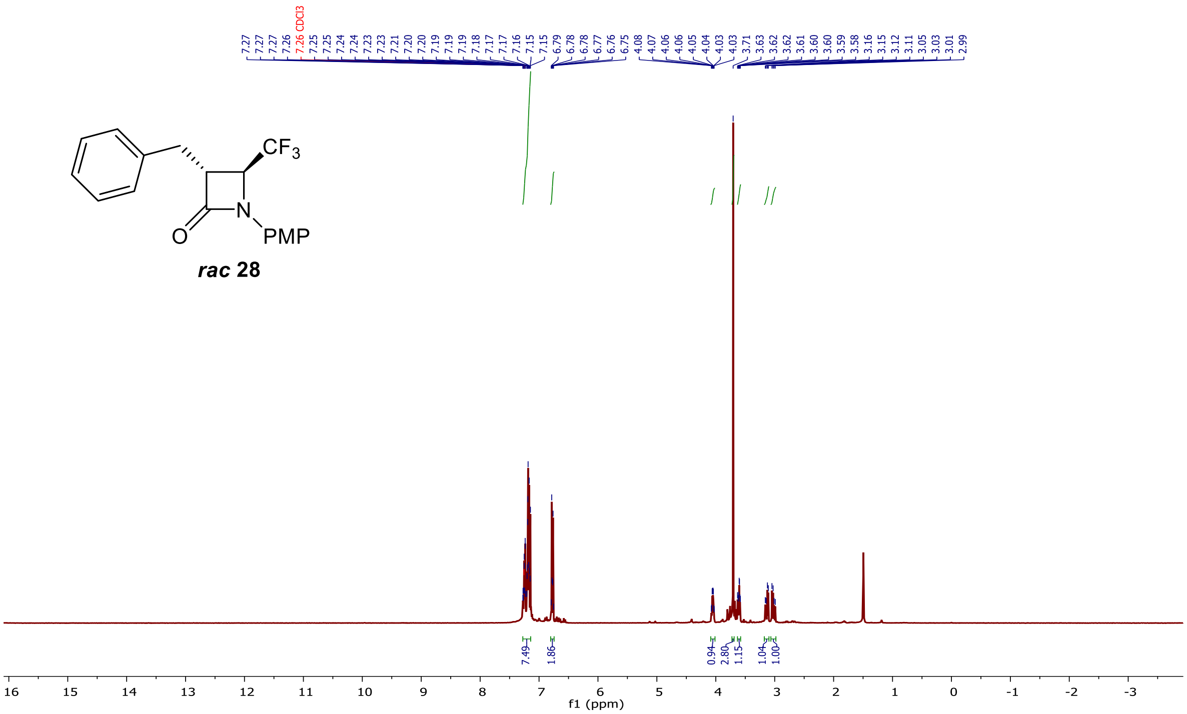

Figure S48. Spectrum ^{1}H NMR ($CDCl_3$, 400 MHz) of *rac* **28**

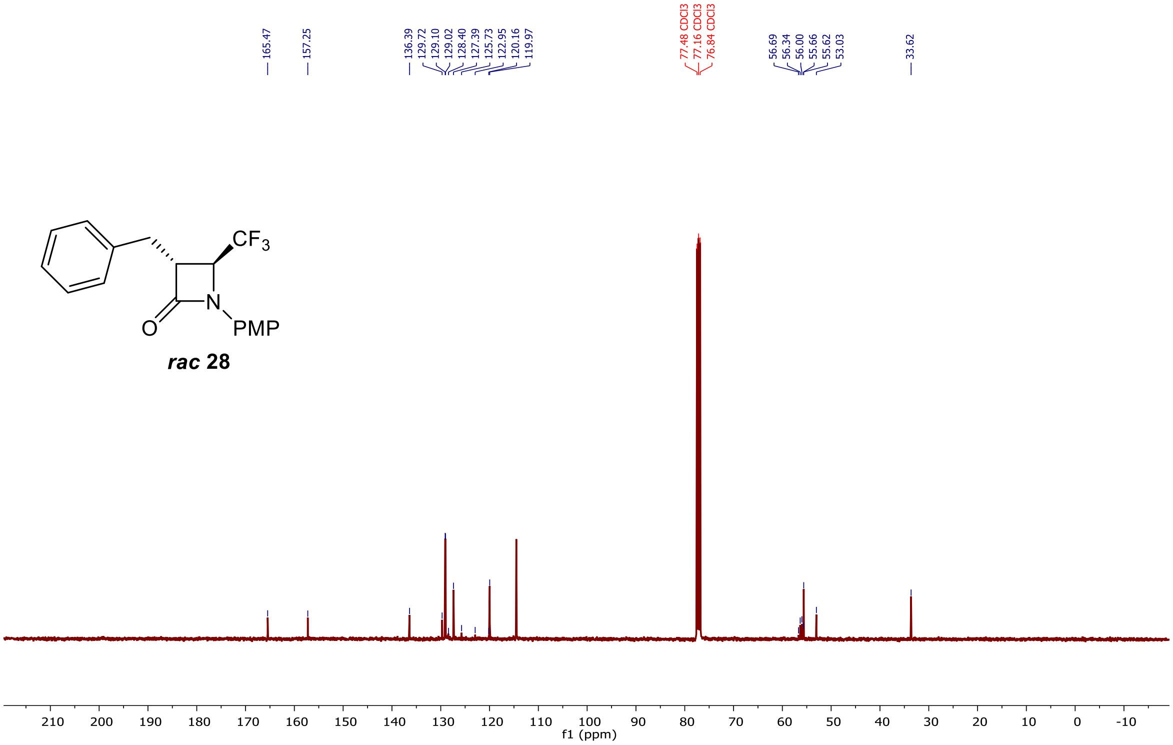

Figure S49. Spectrum ^{13}C NMR ($CDCl_3$, 101 MHz) of *rac* **28**

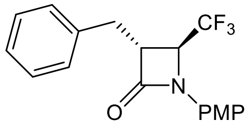

***rac* 28**

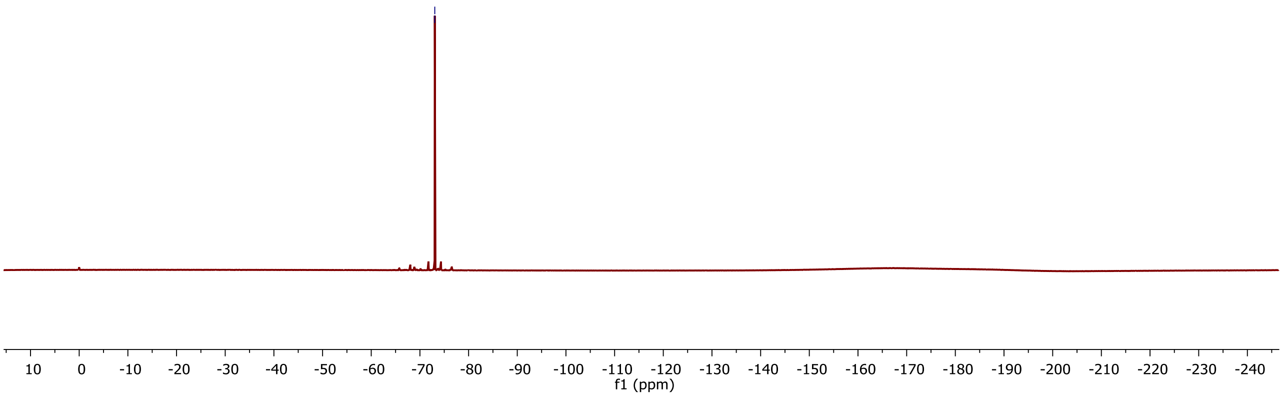

Figure S50. Spectrum ^{19}F NMR ($CDCl_3$, 376 MHz) of *rac* **28**

6. Examples of 2D NOESY ^{1}H-^{1}H and HOESY ^{1}H-^{19}F NMR spectra

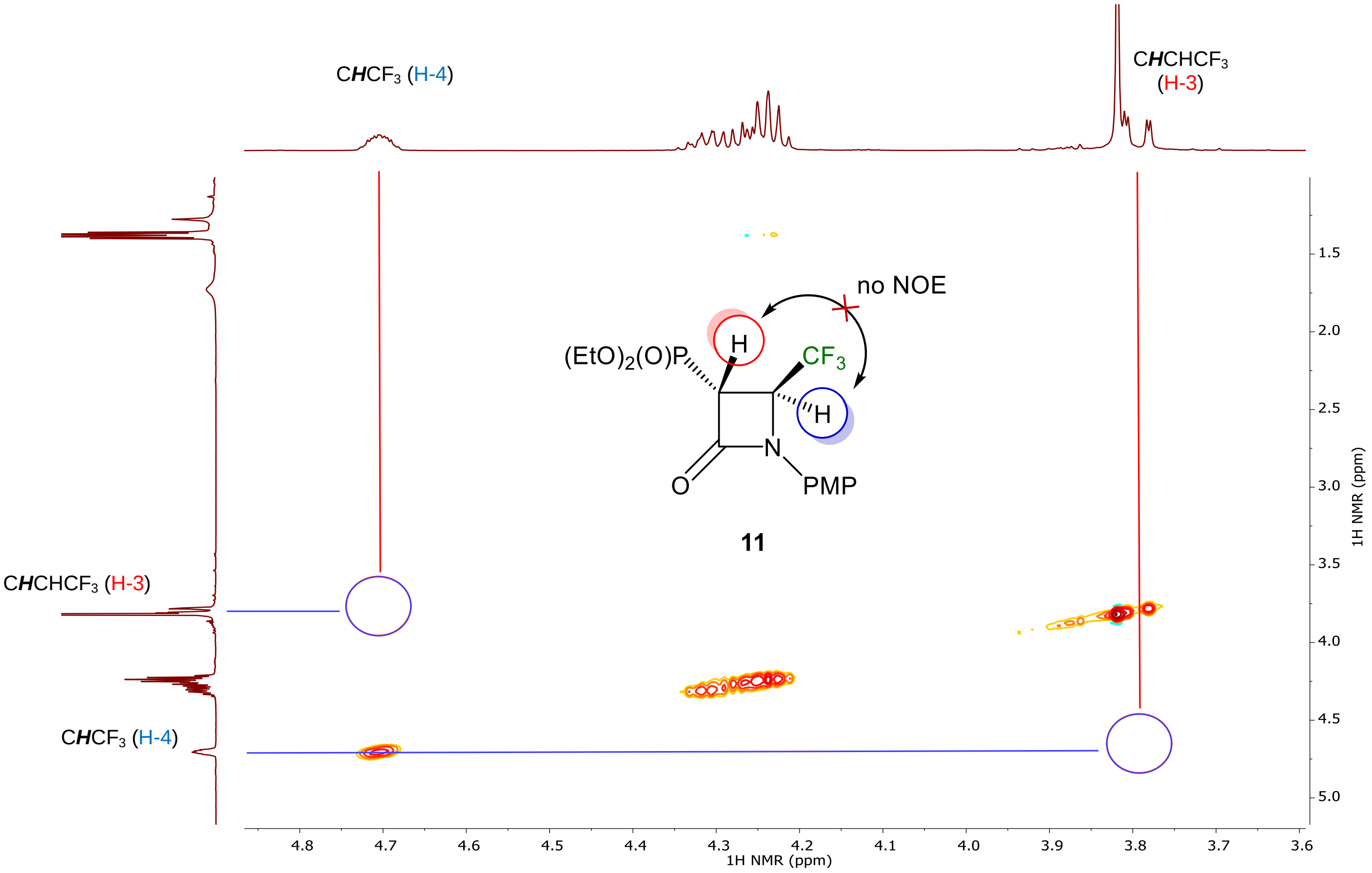

Figure S51. The *trans* configuration of *N*-PMP 3-phosphono-4-CF3-*β*-lactam **11** proved by 2D ^{1}H-^{1}H NOESY NMR, due to absence of NOE interactions in **11** between H-3 and H-4

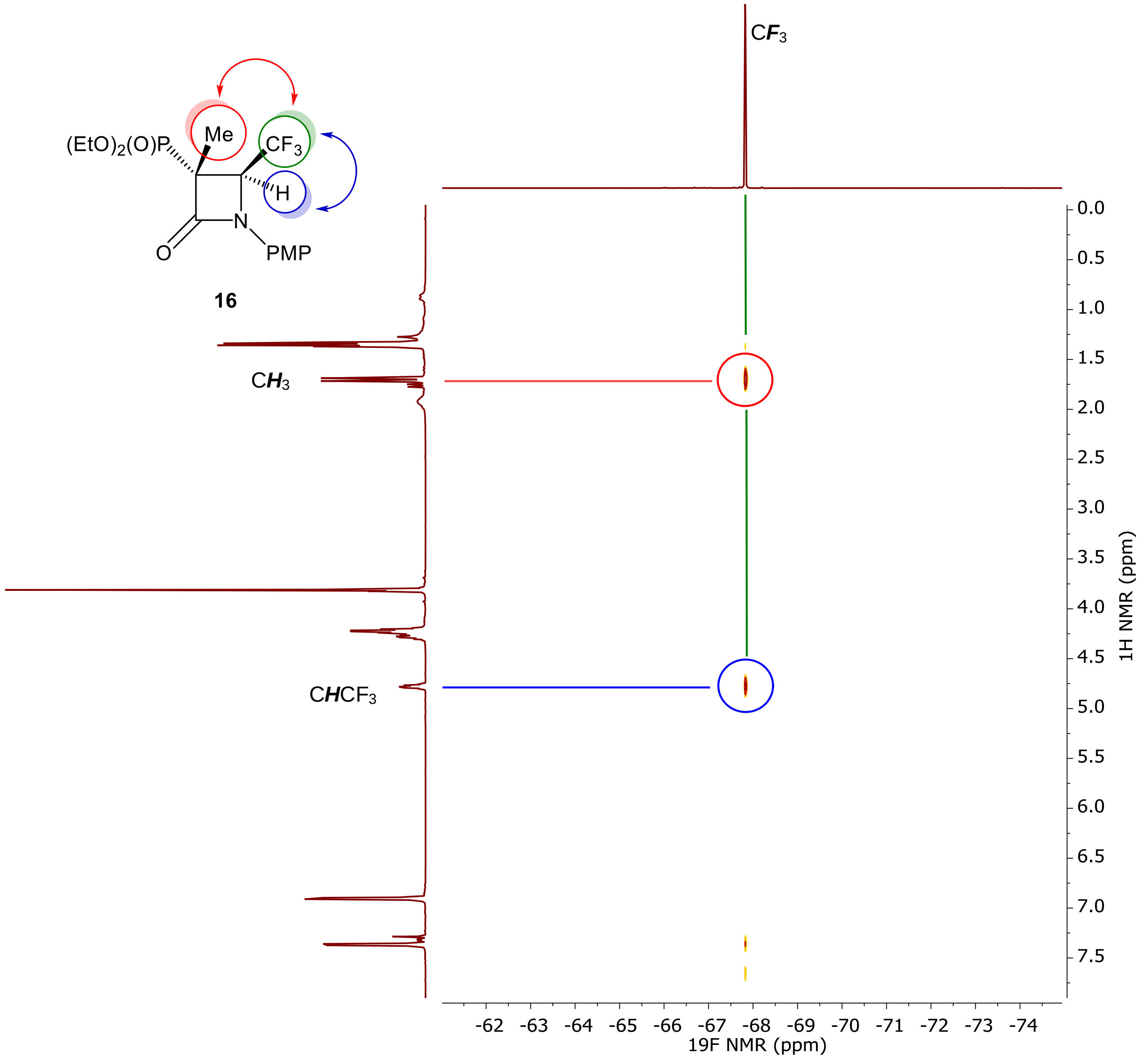

Figure S52. The 2D ^{1}H-^{19}F HOESY NMR spectrum of phosphonate derivative of 3-Me-4-CF_3-β-lactam **16** shows the correlation between CF_3 group and Me moiety

7. Reactions of 3-Allyl, 3-Bn, 3-CO_2Et-4-CF_3-PMP (27-29) lactams with ClP(O)(OEt)$_2$

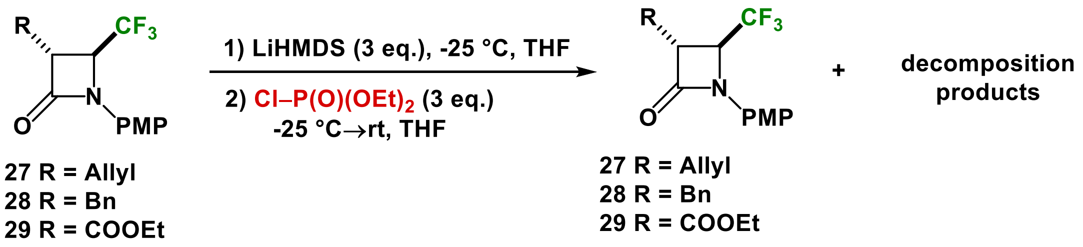

Scheme S1. The synthesis attempts to other of *N*-PMP phosphonated 3-monosubstituted-4-CF_3-*β*-lactams.

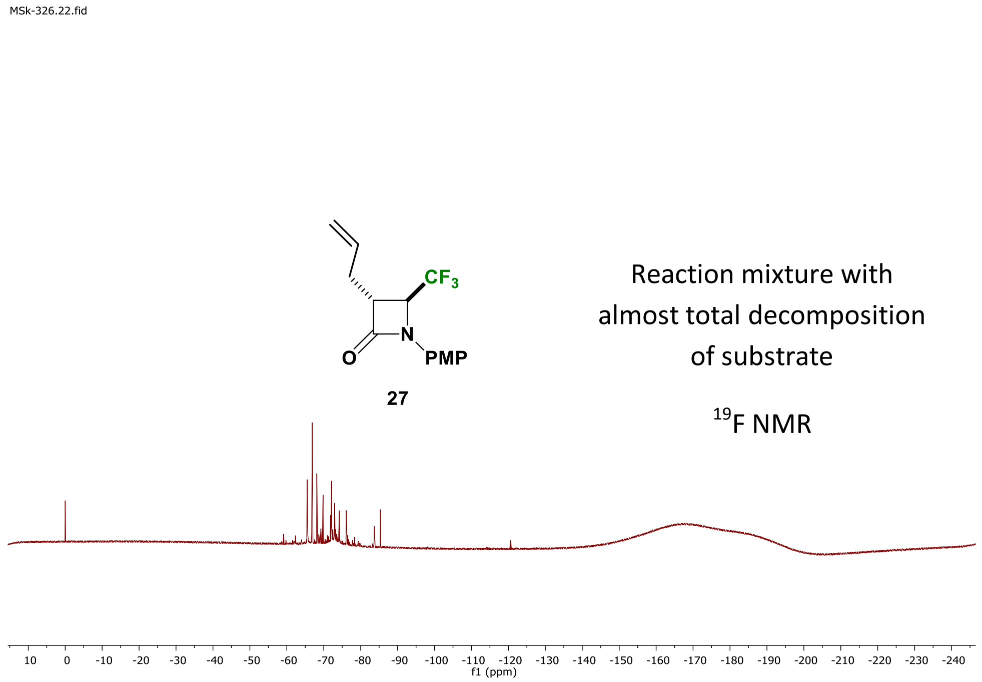

Figure S53. Spectrum ^{19}F NMR ($CDCl_3$, 376 MHz) from the reaction of *rac* **27** with ClP(O)(Et)$_2$

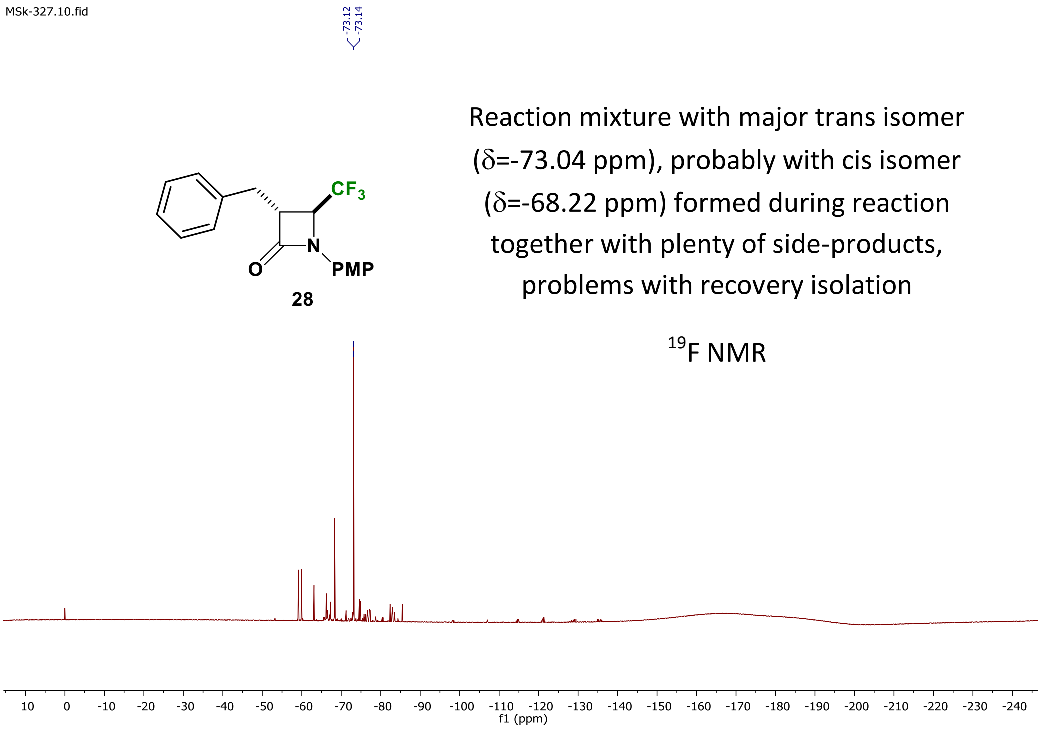

Figure S54. Spectrum ^{19}F NMR ($CDCl_3$, 376 MHz) from the reaction of *rac* **28** with ClP(O)(Et)

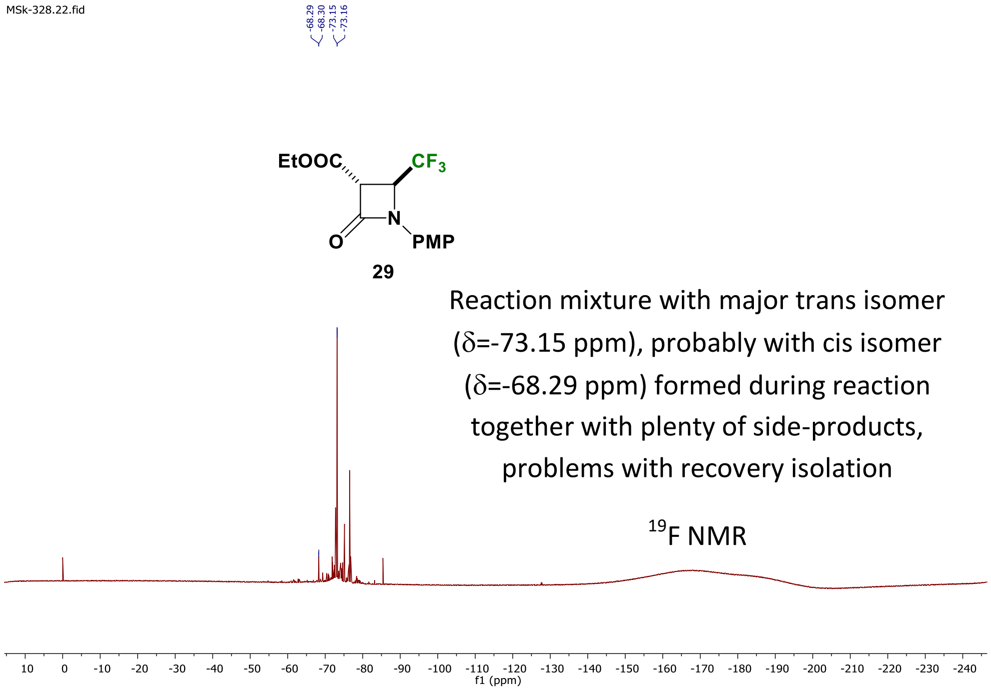

Figure S55. Spectrum ^{19}F NMR ($CDCl_3$, 376 MHz) from the reaction of *rac* **29** with ClP(O)(Et)

8. **Cis/Trans isomerization process based on NMR and GCMS investigation of the crude reaction mixtures**

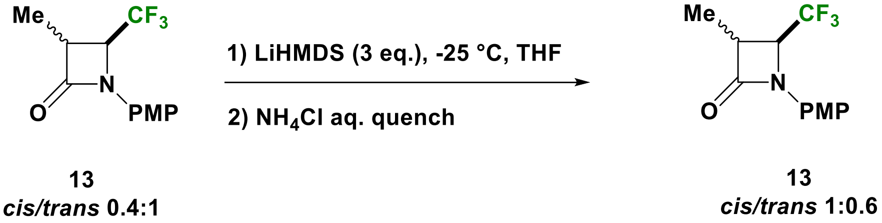

Scheme S2. Test reaction of *cis*/*trans* mixture of 3-Me-4-CF_3-PMP lactam **13** under substitution conditions, without the presence of any electrophile, quenched with NH_4Cl

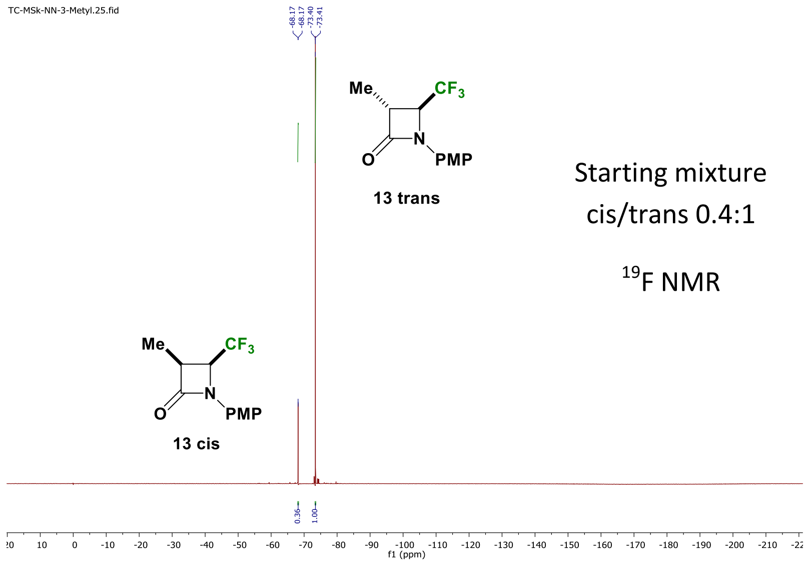

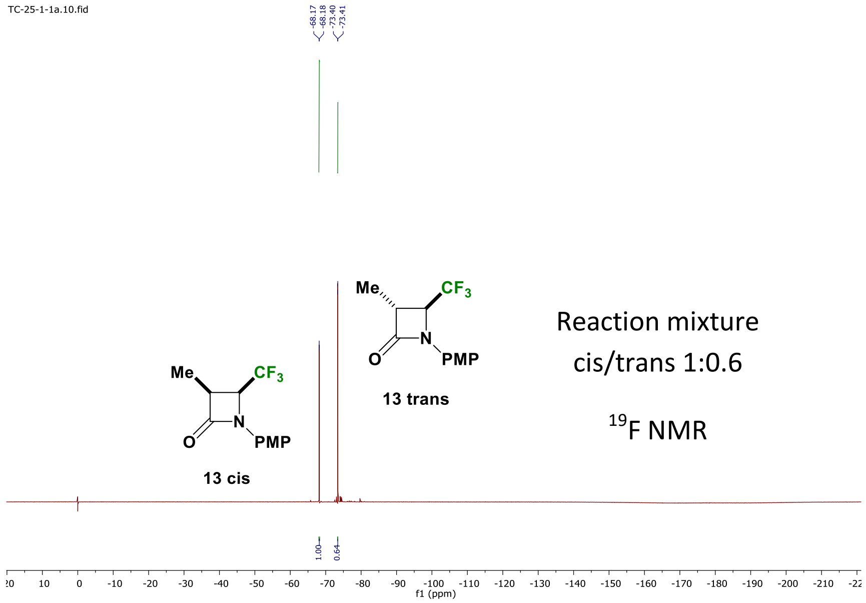

Figure S56. Spectra ^{19}F NMR ($CDCl_3$, 376 MHz) before (top) and after (bottom) reaction of *cis/trans* mixture of *rac* **13** with LiHMDS, followed by quenching with NH_4Cl

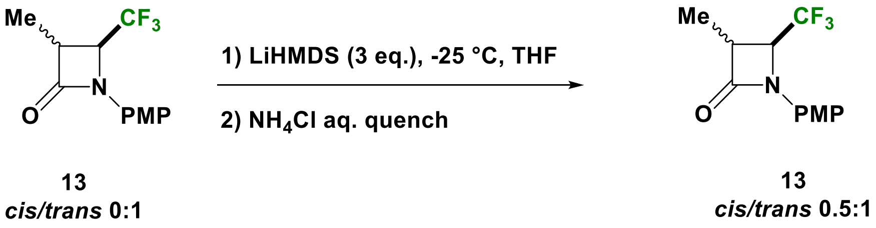

Scheme S3. Test reaction of pure *trans* isomer of 3-Me-4-CF_3-PMP **13** lactam under substitution conditions, without the presence of any electrophile, quenched with NH_4Cl

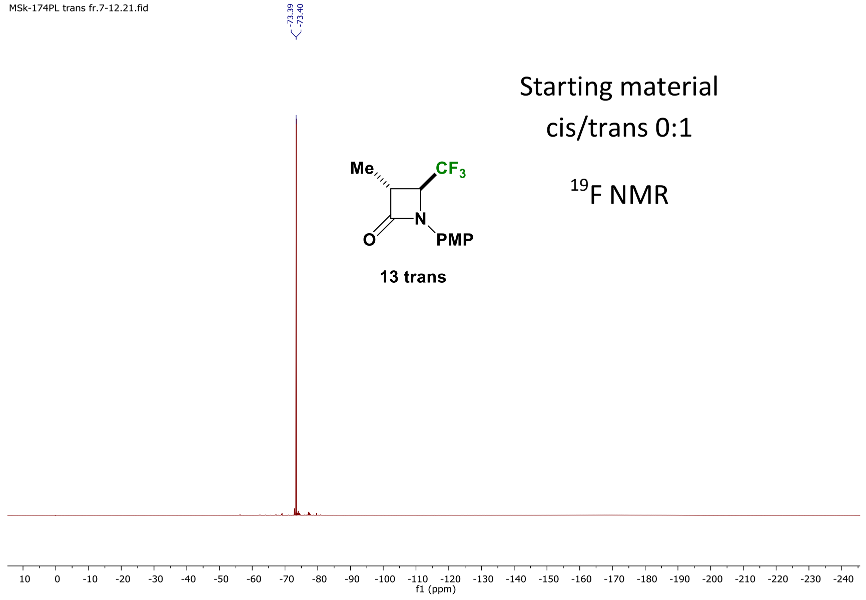

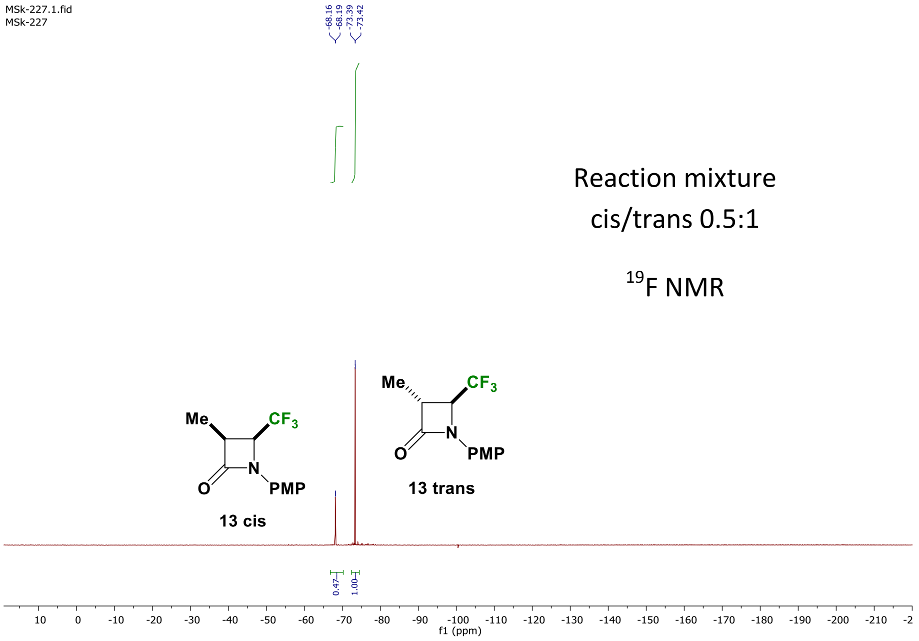

Figure S57. Spectra ^{19}F NMR ($CDCl_3$, 376 MHz) before (top) and after (bottom) reaction of *trans rac* **13** with LiHMDS, followed by quenching with NH_4Cl

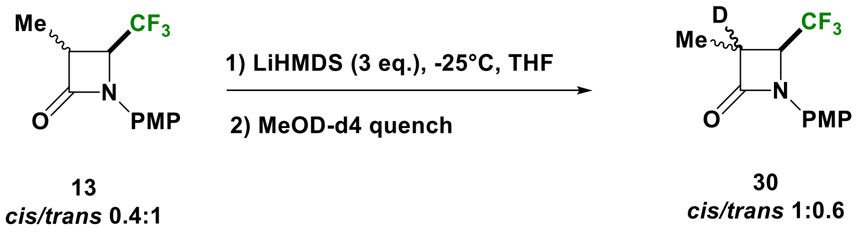

Scheme S4. Reaction of 3-Me-4-CF_3-PMP **13** lactam with different source of proton (quenching with MeOD-d4)

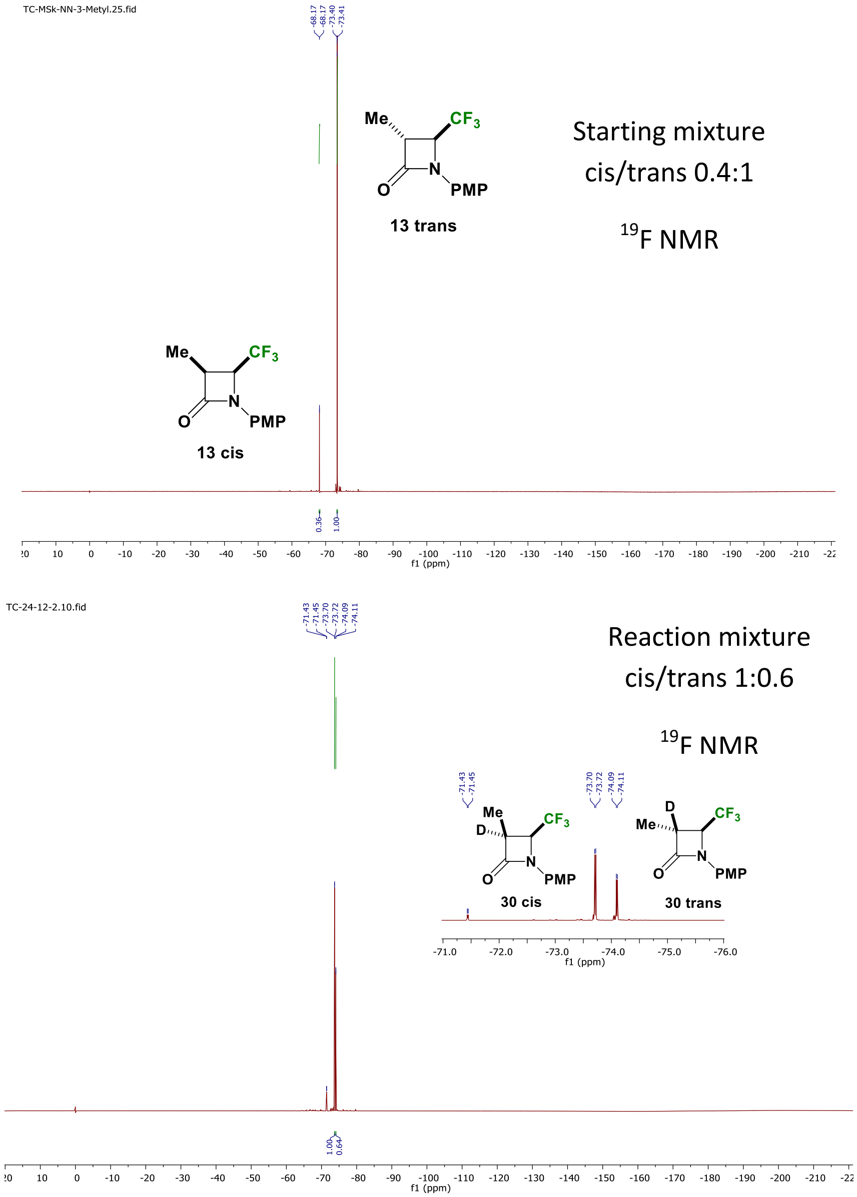

Figure S58. Spectra ^{19}F NMR ($CDCl_3$, 376 MHz) before (top) and after (bottom) reaction of *cis/trans* mixture of *rac* **13** with LiHMDS, followed by quenching with MeOD-d4

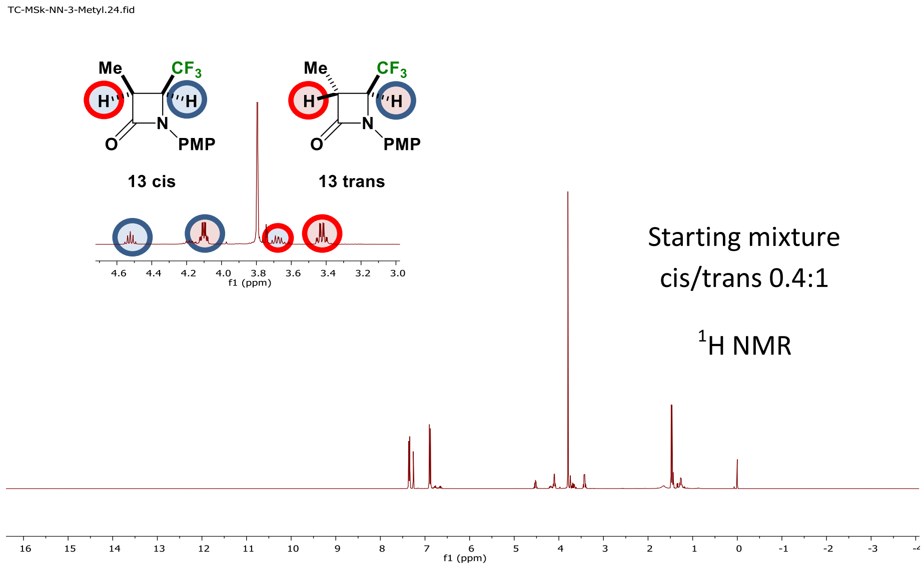

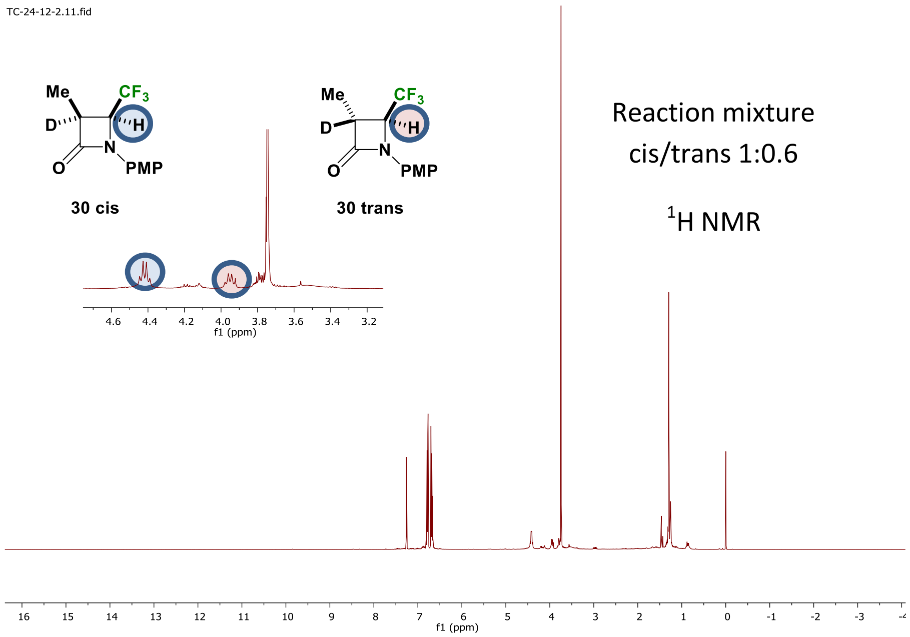

Figure S59. Spectra ^{1}H NMR ($CDCl_3$, 101 MHz) before (top) and after (bottom) reaction of *cis/trans* mixture of *rac* **13** with LiHMDS, followed by quenching with MeOD-d4

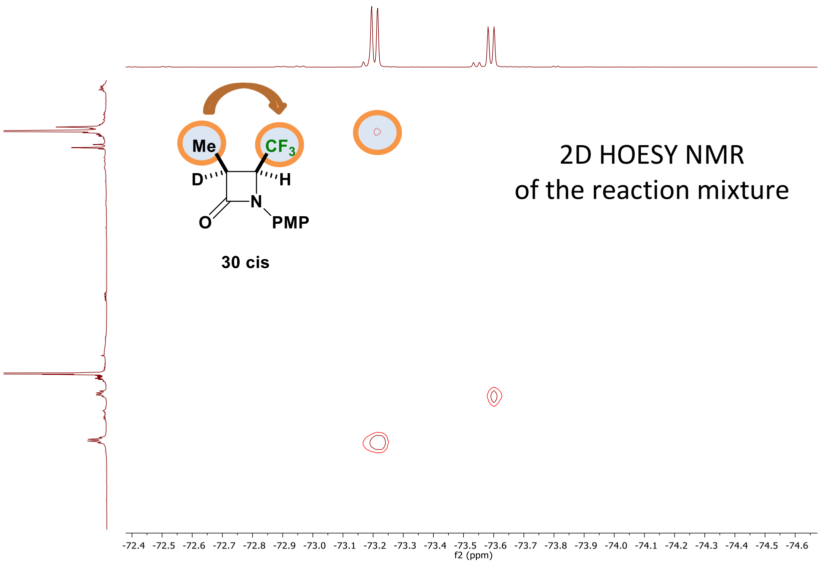

Figure S60. The 2D ^{1}H-^{19}F HOESY NMR spectrum after the reaction of *cis/trans* mixture of *rac* **13** with LiHMDS, followed by quenching with MeOD-d4

Analysis Report

Agilent Trusted Answers

Sample Information

**Name**	CT_24_12_2	**Data File Path**	F:\Nowe wyniki ms\Krzysztof\CT_24_12_2.D
**Sample ID**		**Acq. Time (Local)**	1/9/2025 9:17:38 AM (UTC+01:00)
**Instrument**	GCMS	**Method Path (Acq)**	D:\MassHunter\GCMS\1\methods\STANDARD-toluen.M
**MS Type**	Q	**Version (Acq SW)**	MassHunter GC/MS Acquisition 10.0.384.1 14-Feb-2019 Copyright © 1989-2018 Agilent Technologies, Inc.
**Inj. Vol. (ul)**	1	**IRM Status**	
**Position**	2	**Method Path (DA)**	F:\Nowe wyniki ms\Krzysztof\CT_24_12_2.D\Results\Qual\Version4\default.m
**Plate Pos.**		**Target Source Path**	
**Operator**		**Result Summary**	

Sample Chromatograms

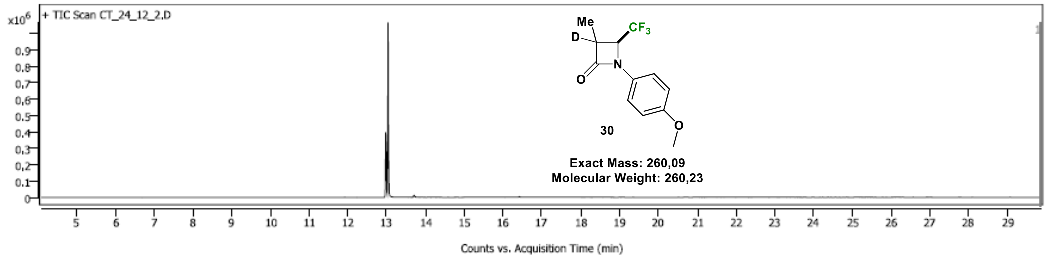

Sample Spectra

+ Scan (rt: 12.972 min)

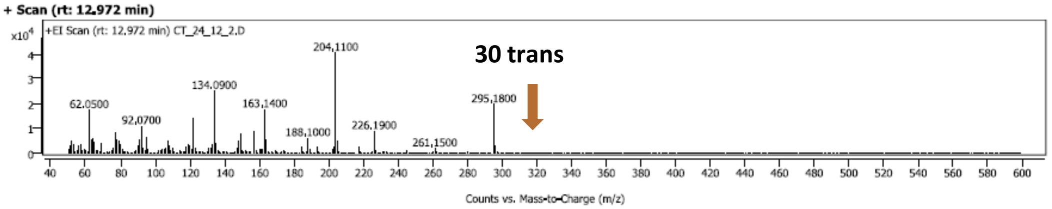

Analysis Report

Agilent | Trusted Answers

Spectrum Peaks

m/z	Z	Abund	Abund %	m/z (Calc)	Diff (ppm)	Ion Species	Formula	Ion Type
202.0500		1519	3.74					
203,1400		2435	6,00					
204,1100	1	40585	100,00					
205.0900	1	4735	11.67					
217.1300		2372	5.84					
226,1900	1	8868	21,85					
227,1700	1	1158	2,85					
231.1900		695	1.71					
233.0900		706	1.74					
245.0600		986	2.43					
261,1500		1854	4,57					
295,1800	1	19710	48,56					
296.1600	1	2778	6.85					

+ Scan (rt: 13.032 min)

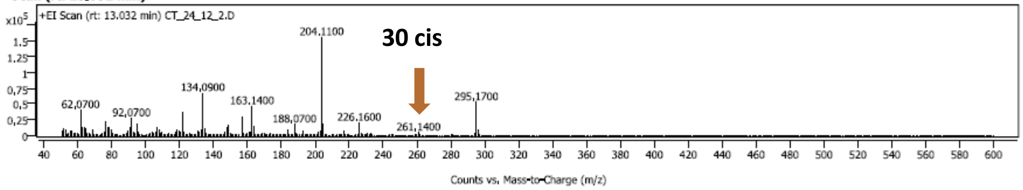

Figure S61. The GC-MS analysis after the reaction of *cis/trans* mixture of *rac* **13** with LiHMDS, followed by quenching with MeOD-d4

9. Attempt to C-3 methylation of 3-phosphonated 4-CF$_3$-*β*-lactam

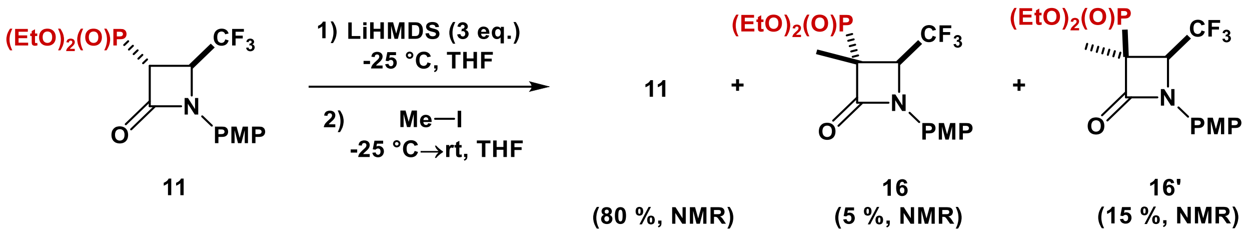

Scheme S5. Attempt to C-3 methylation of 3-phosphonated 4-CF$_3$-*β*-lactam.

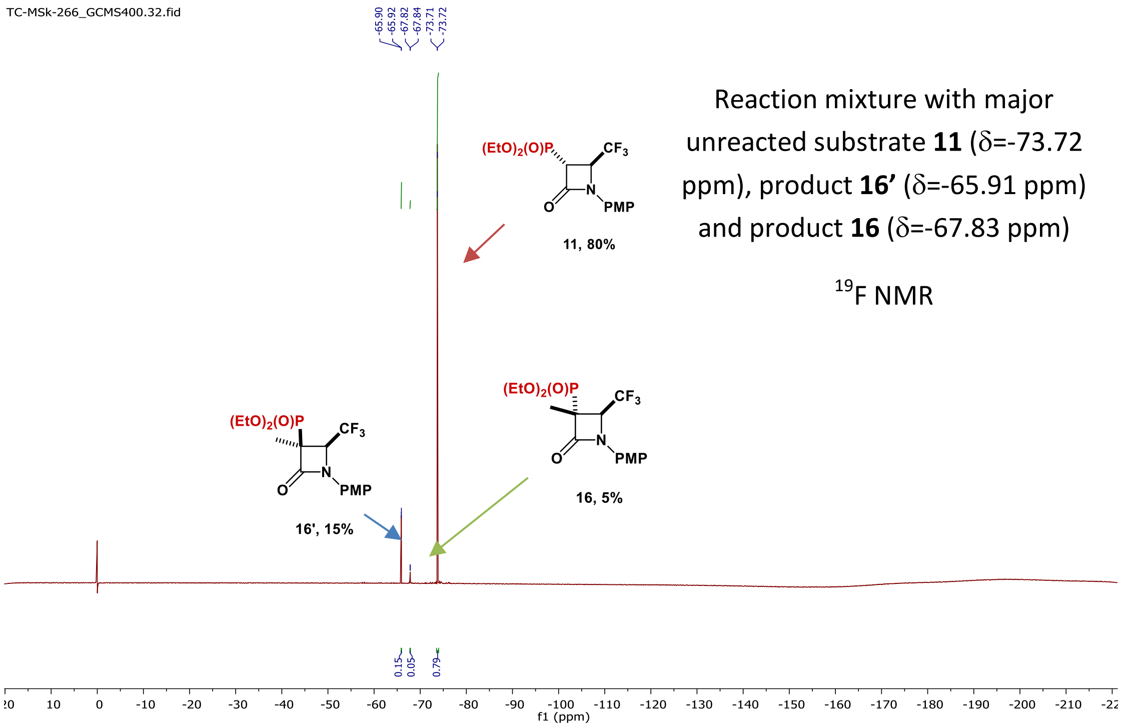

Figure S62. Spectrum ^{19}F NMR (CDCl$_3$, 376 MHz) after the methylation reaction of *rac* **11** with MeI

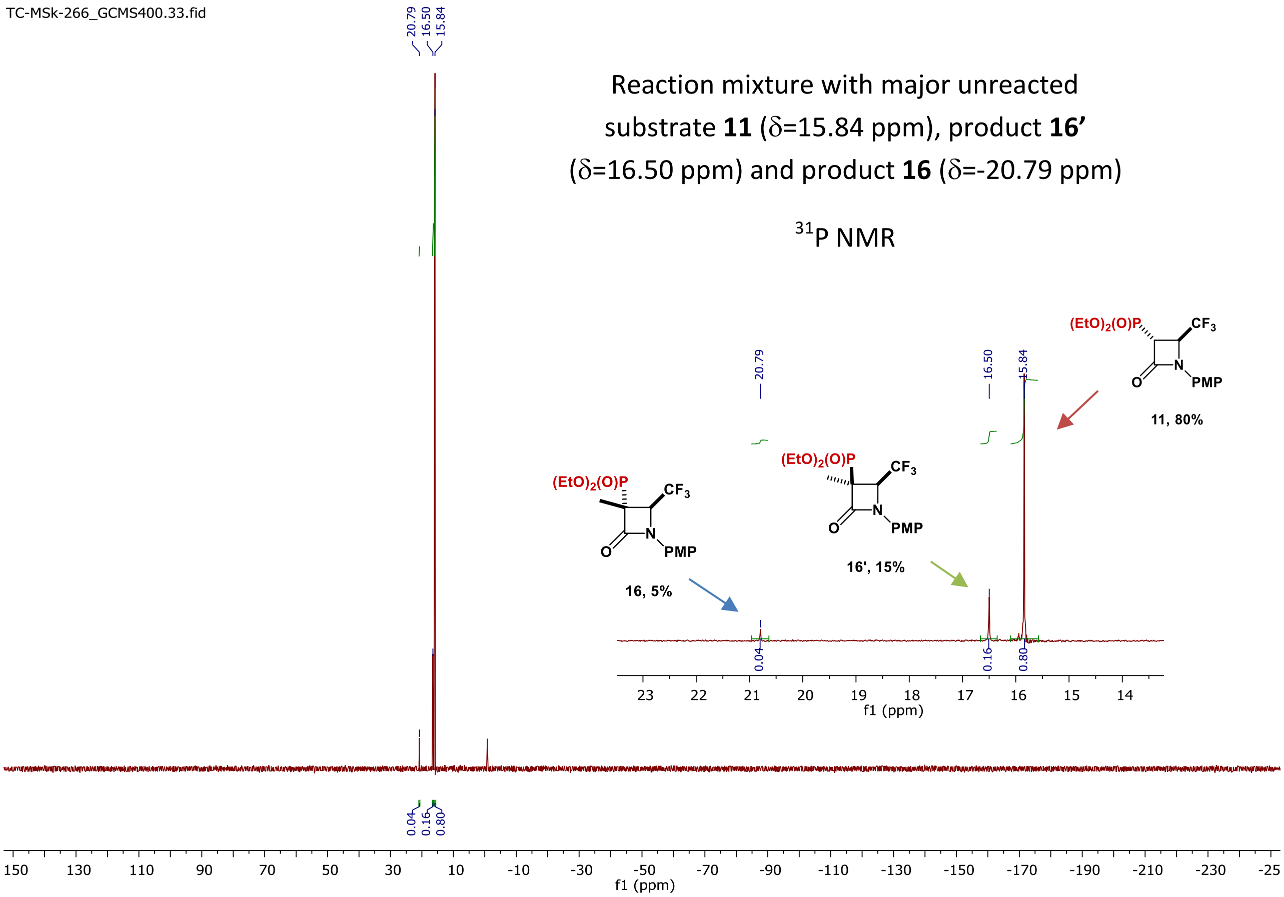

Figure S63. Spectrum ^{31}P NMR ($CDCl_3$, 243 MHz) after the methylation reaction of *rac* **11** with MeI

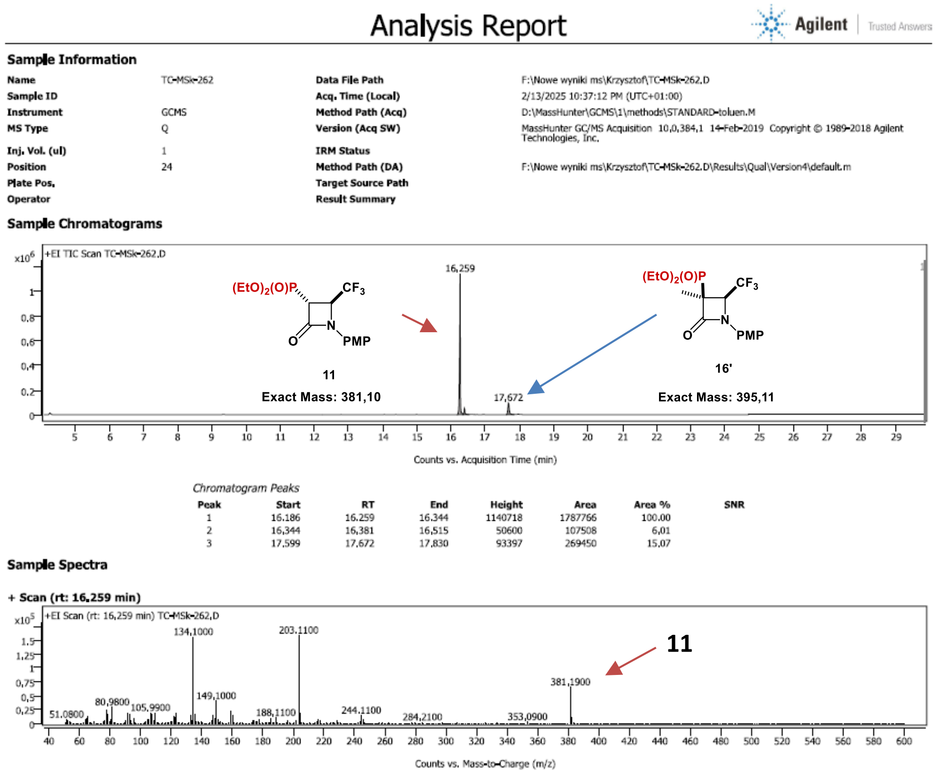

Analysis Report

Sample Information

**Name**	TC-MSk-262	**Data File Path**	F:\Nowe wyniki ms\Krzysztof\TC-MSk-262.D
**Sample ID**		**Acq. Time (Local)**	2/13/2025 10:37:12 PM (UTC+01:00)
**Instrument**	GCMS	**Method Path (Acq)**	D:\MassHunter\GCMS\1\methods\STANDARD-toluen.M
**MS Type**	Q	**Version (Acq SW)**	MassHunter GC/MS Acquisition 10.0.384.1 14-Feb-2019 Copyright © 1989-2018 Agilent Technologies, Inc.
**Inj. Vol. (ul)**	1	**IRM Status**	
**Position**	24	**Method Path (DA)**	F:\Nowe wyniki ms\Krzysztof\TC-MSk-262.D\Results\Qual\Version4\default.m
**Plate Pos.**		**Target Source Path**	
**Operator**		**Result Summary**	

Sample Chromatograms

Chromatogram Peaks

Peak	Start	RT	End	Height	Area	Area %	SNR
1	16.186	16.259	16.344	1140718	1787766	100.00	
2	16,344	16,381	16,515	50600	107508	6,01	
3	17,599	17,672	17,830	93397	269450	15,07	

Sample Spectra

+ Scan (rt: 16,259 min)

Analysis Report

Agilent | Trusted Answers

Spectrum Peaks

m/z	Z	Abund	Abund %	m/z (Calc)	Diff (ppm)	Ion Species	Formula	Ion Type
244.1100		14604	9.20					
245,1100		7939	5,00					
256,1100		2257	1,42					
258.1100		1592	1.00					
284.2100		2451	1.54					
325,1000		1589	1,00					
333,1000		1814	1,14					
353.0900		3032	1.91					
381.1900	1	65460	41.23					
382.0800	1	10596	6.67					
383,1800	1	1713	1,08					

+ Scan (rt: 16.393 min)

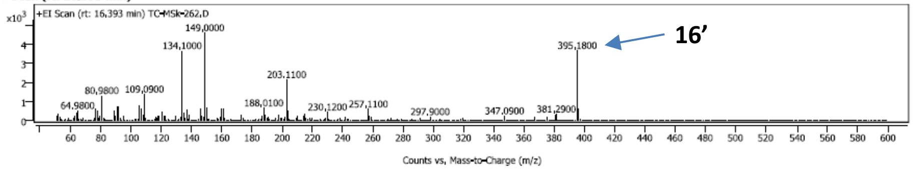

Figure S64. The GC-MS analysis after the methylation reaction of *rac* **11** with MeI

10.References

1. Trulli, L.; Raglione, V.; Fioravanti, S., Selective Synthesis of Trifluoromethyl β-Lactams by a Zn-Promoted 2-Bromo Ester Addition on C-CF3-Substituted Aldimines. *Eur. J. Org. Chem.* **2018,** *2018* (27-28), 3743-3749.
2. Liu, Y.; Chen, J.-L.; Wang, G.-H.; Sun, P.; Huang, H.; Qing, F.-L., 4-CF3-ezetimibe analogs: design, synthesis, and biological evaluation of cholesterol absorption inhibitions. *Tetrahedron Lett.* **2013,** *54* (40), 5541-5543.
3. Decamps, S.; Sevaille, L.; Ongeri, S.; Crousse, B., Access to novel functionalized trifluoromethyl β-lactams by ring expansion of aziridines. *Org. Biomol. Chem.* **2014,** *12* (33), 6345-6348.
4. Dao Thi, H.; Danneels, B.; Desmet, T.; Van Hecke, K.; Van Nguyen, T.; D'hooghe, M., Synthesis and Applications of 3-Methylene-4-(trifluoromethyl)azetidin-2-ones as Building Blocks for the Preparation of Mono- and Spirocyclic 4-CF3-β-Lactams. *Asian J. Org. Chem.* **2016,** *5* (12), 1480-1491.
5. Gong, Y.; Kato, K., Nucleophilic reactions with α-trifluoromethyl imine and N,O-disubstituted aminal: synthesis of β-trifluoromethyl β-anilino esters. *J. Fluor. Chem.* **2001,** *111* (1), 77-80.
6. Skibińska, M.; Kaźmierczak, M.; Milcent, T.; Cytlak, T.; Koroniak, H.; Crousse, B., Direct Access to Substituted 4-CF3 β-Lactams at the C-3 Position. *Front. Chem.* **2019,** *7*, Article 526.
